# Effects of Blueberry Supplementation on Depression and Anxiety Symptoms in a Rural Louisiana Population

**DOI:** 10.3390/nu17233720

**Published:** 2025-11-27

**Authors:** Katy E. Venable, D. Parker Kelley, Alec Jeansonne, Robbie Beyl, Samia O’Bryan, Venugopal Vatsavayi, Samuel Jones, Charles C. Lee, Joseph Francis

**Affiliations:** 1 Department of Comparative Biomedical Sciences, School of Veterinary Medicine, Louisiana State University, Baton Rouge, LA 70803, USA; kvenab3@lsu.edu (K.E.V.); parker.kelley@ucsf.edu (D.P.K.); samia.obryan@gmail.com (S.O.); jfrancis@lsu.edu (J.F.); 2 Louisiana Health Care Practitioners, LLC, Cottonport, LA 71327, USA; alec@collectivehcs.com; 3 Pennington Biomedical Research Center, Louisiana State University, Baton Rouge, LA 70808, USA; robbie.beyl@pbrc.edu; 4 Baton Rouge Clinic, Baton Rouge, LA 70808, USA; venuvatsavai@gmail.com; 5 Renal Associates of Baton Rouge, LLC, Baton Rouge, LA 70808, USA

**Keywords:** major depressive disorder, depression, anxiety, blueberry supplementation

## Abstract

**Background/Objectives**: Blueberries are an important nutraceutical due to their excellent nutritional profile, with particularly high levels of polyphenols and anthocyanins. These compounds can improve mood, cognition, and health. As such, blueberry consumption can potentially benefit those coping with depression and anxiety. In this regard, there is an unmet need for novel, effective, and accessible treatments for these conditions, particularly in rural communities, where specialized health care is often limited. **Methods**: Therefore, we conducted a double-blind, randomized pilot study in a rural population to assess whether daily blueberry supplementation affected symptoms of anxiety and depression. We employed a crossover design to test the effects of 12 weeks daily ingestion of 24 g of whole freeze-dried blueberry powder versus placebo on symptoms of depression and anxiety in those diagnosed with a depressive or anxiety disorder including major depressive disorder (MDD) and generalized anxiety disorder (GAD). We collected behavioral data (HDRS, GAD-7, MDI) at baseline, mid-, and post-treatment timepoints. We collected blood, serum, plasma, and behavioral data (HDRS, GAD-7, MDI) at baseline, mid-, and post-treatment timepoints. We measured inflammatory cytokines IL-1β, IL-6, TNF-α, IFN-γ, and IL-10 in serum, CRP in whole blood, and performed global metabolomics in plasma. **Results**: Blueberries significantly reduced symptoms of depression and anxiety compared to placebo. CRP and inflammatory cytokine levels were unaffected. Our global metabolomic measures suggested that different metabolites were differentially affected at the middle and post-intervention timepoints in the study. **Conclusions**: Overall, this study found potential improvements in symptoms of depression and anxiety following daily blueberry supplementation, although the biochemical mechanisms underlying these behavioral improvements remain unresolved.

## 1. Introduction

Depression and anxiety disorders are common, costly, and major public health concerns. Major depressive disorder (MDD) and generalized anxiety disorder (GAD) affect around 5% and 3% of the populace in the United States, respectively [1,2]. For MDD, treatments typically rely on a combination of psychotherapy and medication [3]. For GAD, psychotherapy and medication are also typical first-line treatments, although official guidelines have not been established by the APA [4]. Although MDD and GAD affect a similar proportion of individuals who live in rural settings, these individuals are less likely to seek treatment for mental health issues and have few or no options when seeking the support of mental health care professionals, particularly specialized or highly trained professionals [5,6,7]. Thus, pharmaceuticals are often the first-line treatment and only treatment option for patients who present to rural clinics with mental health disorders.

Moreover, rural populations are generally overlooked in most research studies, particularly for mental health. Study sites for rural participants usually include only those close to urban areas, typically near universities or institutions conducting such research, which requires significant time and travel for rural participants and creates a barrier to participation. In the literature, only two health studies explicitly report having used Rural Health Clinics (RHCs) for either recruitment or as a study site: (1) one health study focused on rural populations and used Rural Health Clinics (RHCs) for participant recruitment, but the study itself was carried out over the phone [8], and (2) a study in New Mexico which used RHCs as a study sites, but which collected no physiological samples [9]. In the rural areas in Louisiana that we investigated, there is little access to mental health care or mental health care professionals, but plenty of people in need of safe and effective treatment options. Yet, few studies exist on approaches to treating mental health, specifically in underserved, rural areas.

In this regard, nutritional approaches present a simple, safe, accessible, and potentially effective approach in rural settings to improving mental health. Many nutrients are epidemiologically associated with improved mental health conditions, e.g., docosahexaenoic acid (DHA), eicosapentaenoic acid (EPA), various minerals, B vitamins, omega-3 fatty acids, vitamin D, and antioxidants [10,11,12,13]. Such nutrients are abundant in several traditional diets (e.g., Mediterranean diets); these nutrients can positively influence mental health, potentially through interactions with the gut microbiome [14,15,16]. Thus, prebiotic foods that are abundant in such nutrients can potentially lead to improvements in mental health and warrant further investigations [10,11,12,13].

Blueberries have emerged as a potential superfood that contain important micronutrients which have demonstrated benefits for memory, cognition, mood, and health; thus, they may be an ideal food for improving symptoms of depression and anxiety in both rural and urban settings [11]. The polyphenols in blueberries, specifically anthocyanins, produce bioavailable metabolites that are associated with several health benefits [17,18,19,20] and are distributed to different tissues, including the brain [21,22,23,24,25]. In addition, a blueberry diet improved symptoms of anxiety and normalized serotonin (5-HT) levels in the pre-frontal cortex and hippocampus without increasing norepinephrine (NE) in a rodent model of psychosocial stress [26,27]. Reactive oxygen species (ROS) and inflammatory cytokines (including IL-1β) were also normalized in the same regions with blueberry treatment. Other preclinical studies have demonstrated positive effects of blueberries for measures of cognition, memory, and plasticity, and that these effects were accompanied by increases in glutathione peroxidase, ascorbic acid, CREB, ERK1/2 signaling, and BDNF [28,29,30]. Even more compelling is the accumulating evidence suggesting that blueberries positively affect human mood, memory, cognition, and health. For example, acute and long-term blueberry supplementation resulted in beneficial effects on cognition and mood in children and young adults [31,32] as well as older adults [33,34,35]. Physiologically, blueberries enhance systemic endothelial and vascular function and increase resting blood perfusion in brain regions implicated in MDD, GAD, interoception, emotional regulation, and cognitive function [33,34]. Interestingly vascular function and cerebral blood flow regulation is impaired in depression [36] and anxiety [37,38]. Altogether, this preclinical and clinical data demonstrate the potential blueberry has in supporting brain health and warrants further studies on the topic.

So, based on the overlooked mental health needs in rural settings and the established effects of blueberry supplementation, we designed and implemented a nutritional study evaluating the effects of long-term blueberry supplementation on symptoms of depression and anxiety, and biological outcomes associated with MDD and GAD in a rural population in Louisiana. We specifically addressed the following questions: (1) is a study of this nature feasible in a rural health setting and (2) does the data support further research using blueberries as a treatment or adjunct treatment for depression and/or anxiety? Based on our results, this methodology appears adaptable to other rural health studies, and blueberry supplementation may yield significant behavioral improvements for depression and anxiety.

## 2. Materials and Methods

All procedures were approved before recruitment began and carried out under the auspices and approval of the Louisiana State University Institutional Review Board (IRB) (IRB #3762 and #4009; Supplementary Files S1 and S2). All ethical considerations of the study design were reviewed by the Louisiana State University Institutional Review Board (IRB). Importantly, this study did not influence any mental health or general health-related treatment being received by the recruited subjects (see Section 4). The study is registered with ClinicalTrials.gov ID: NCT04398784. This study involved a collaborative effort between researchers from Louisiana State University (LSU) and two Rural Health Clinics (RHCs) in central Louisiana owned and operated by Louisiana Health Care Practitioners LLP.

### 2.1. Recruitment and Survey Study

We employed a rigorous approach to identify and recruit potential participants for the study, which was defined by a priori inclusion/exclusion criteria to obviate any potential selection bias. A modified version of our inclusion/exclusion criteria that was designed to convey this information to participants is available in Supplementary File S3. First, we conducted a brief survey study in the clinics through which we provided potential participants with information about blueberries, depression, and anxiety, and probed if people would be interested in participating in a future study. Those indicating interest in participating were invited to complete the remainder of the survey, in which they filled out a HIPAA agreement and their personal contact information (Supplementary File S4). The initial survey study was conducted at five RHCs in central Louisiana, i.e., the Marksville Family Clinic, Cottonport Family Clinic, Elizabeth Family Clinic, Mansura Family Clinic, and Simmesport Family Clinic, which operate collectively under Louisiana Health Care Practitioners LLP. This stage had multiple objectives, which are as follows: (1) to inform and discuss with clinic clients about the prospective blueberry study and gauge interest, (2) to establish in-person relationships with those interested in participating in the prospective study (described below), and to collect contact information through which we provided interested parties with further information and a preliminary screening questionnaire, (3) to establish professional, in-person relationships with clinic staff, (4) to assess the feasibility and limitations of working in each clinic; and (5) to recruit potential participants for the clinical study. The in-person recruitment was conducted for one day each week for 4 consecutive months. During these visits, informational sessions were provided to clinic staff and nurse practitioners to solicit their feedback and to enable them to inform clients about the study. Surveys were securely collected during our weekly in-person visits to the clinics and were maintained in locked briefcases for travel and stored in locked filing cabinets at LSU. Second, we aired advertisements on local radio stations which included information about how to enroll in the study. Finally, we held a hotdog social for the community at which we also recruited for the study. Overall, recruitment took place over the course of a 6-month period.

Prospective participants completed an initial screening process over the phone to assess eligibility. Interested participants then completed a thorough written informed consent process in-person, which included signing a HIPAA agreement, and were assessed for study eligibility with baseline psychometric measures (i.e., MDI, GAD-7, a custom nutrition and lifestyle survey (Supplementary File S5), and a screening blood draw. All the specified inclusion and exclusion criteria can be found in the informed consent documents located in Supplementary File S3. Specifically, regarding the inclusion/exclusion criteria, we required the following conditions to be met: participants (1) must have had a pre-existing diagnosis of MDD or GAD; (2) had a current MDD diagnosis confirmed by the MDI, or had clinically significant GAD symptoms measured by the GAD-7.

### 2.2. Pilot Study Design and Timeline

**Figure 1:**
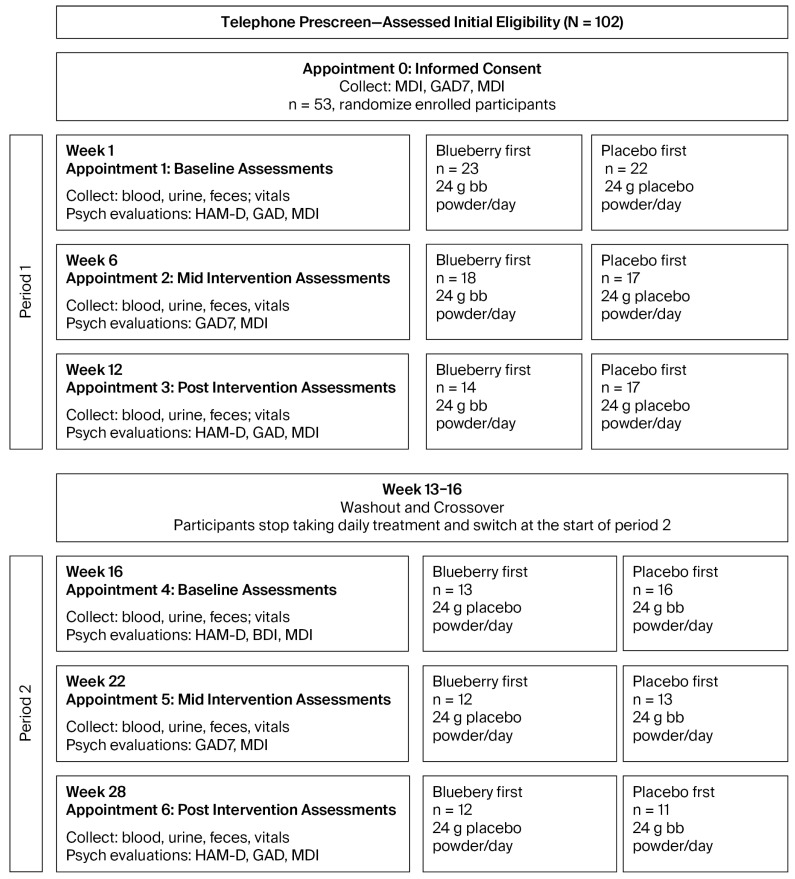
Study design and flow chart. An initial telephone pre-screen was used to assess eligibility. This was followed by an initial appointment to provide information and written informed consent. The trial was constructed within two periods (Period 1 and Period 2), with a four-week washout period separating the periods. During each period, assessments were obtained at baseline (bl), middle (mid), and post-intervention periods. The number of participants (n) at each stage is indicated in the corresponding boxes.

We employed a randomized, double-blind, placebo-controlled crossover design, with two 12-week treatment periods separated by a 4-week washout period (Figure 1). Each period had baseline (bl), mid-, and post-assessment timepoints during which we collected behavioral data, in addition to whole blood, serum, and plasma. At all timepoints, we collected the Major Depression Inventory (MDI) and the Generalized Anxiety Disorder 7-Item Questionnaire (GAD-7), which are self-reported metrics of MDD and GAD, respectively. At bl and post timepoints, we also conducted the Structured Interview Guide for the Hamilton Depression Rating Scale (SIGH-D), a clinician-administered, structured interview which is diagnostic for the symptoms and severity of depression, based on the Hamilton Depression Rating Scale (HDRS), a gold standard metric in studies of MDD [39].

Participants were reimbursed $25 for each completed appointment (6 total), and an additional $50 for completing the study in full, for a total reimbursement of $200. Participants were not reimbursed for missed appointments, but they were allowed to continue participation.

Our highest priority was to adhere to strict ethical standards to prevent adversely affecting the well-being of the study’s participants. This study was designed to determine if adding 24 g/day of freeze-dried blueberries could improve symptoms of MDD and GAD in participants already undergoing standard of care. Thus, we did not influence any of participants’ medication or treatment decisions and blueberry supplementation did not disrupt or interfere with any psychiatric care that participants were receiving from their health care providers. Participants who were prescribed medications continued to take those medications as directed by their physicians and were not prohibited from starting or stopping any medications. The specific drug classes that participants were on, and percentages for each, are included in Supplementary File S6, data repurposed from our prior publication [11]. However, we did request that participants let us know about any changes in their medication or therapeutic regiments during the course of the study. Importantly, we did not present blueberry treatment as a substitute or replacement for regular psychiatric care. While we did not observe any adverse events or serious adverse events in this study, our protocols required that any adverse or serious adverse events observed during the study, including any serious decline in mental health in any participant, be immediately reported to the study’s physicians and participants’ health care providers to immediately facilitate the requisite medical attention.

### 2.3. Randomized Group Assignments for Blueberry Supplementation and Placebo Control

We implemented a pseudo-randomized matched-pairs design to eliminate any potential selection bias. First, we generated matched pairs based on age, sex, MDI scores, and CRP levels measured at the screening visit. Next, we pseudo-randomized participants into either the blueberry-first or placebo-first groups using the Google random number generator, using a balanced design. To illustrate the randomization process, we first matched participants based on four criteria: age, sex, baseline MDI, and baseline CRP, ensuring that, at baseline, the groups each had people of comparable age, sex, MDI depression scores, and levels of inflammation. Next, to select a participant, we randomly generated a number from 1—the total number of remaining unassigned participants using the Google random number generator. After selecting a participant, we generated either a 1 or a 2 (50% odds) using the Google random number generator to randomly assign that participant to either the blueberry-first or placebo-first groups and their matched pair was assigned to the other group. This same assignment procedure was then iteratively completed for all matched pairs, one-by-one, until all participants were assigned to treatment groups. After matching, we confirmed that there were no statistically significant differences between the groups on any of the four criteria, ensuring that our balanced randomization procedure worked and that selection bias did not impact group assignment.

Participants ingested either 24 g whole, freeze-dried blueberry powder or a calorie-, taste-, and color-matched placebo powder mixed in water daily during 12-week treatment periods (information for powder and placebo is included in Supplementary File S7). Blender bottles were provided to all participants at the start of the study for ease of preparation. Participants assigned to the “blueberry-first” condition received blueberry powder in Period 1 and placebo powder in Period 2; participants assigned to the “placebo-first” condition received placebo powder in Period 1 and blueberry powder in Period 2 (Figure 1).

At bl and mid timepoints, participants received a sufficient number of unmarked, individually packaged 24 g treatment pouches to last until their next appointment. These were labeled with a code (i.e., alpha or omega), the meaning of which was unknown to clinic staff, study staff, and both researchers who interacted directly with participants, and distributed in opaque boxes to further ensure that researchers remained blinded to participant treatment allocation. Participants were instructed to store their packages in their freezer or refrigerator, to time their ingestion for around the same time every day. As a metric of compliance, participants were instructed to keep empty packets in their box and return them at their next appointment The used packets were counted by research assistants, who calculated the percentage used to measure net days of treatment (see Section 4).

### 2.4. Behavioral Data Collection

All behavioral data were collected on paper and entered in a database at a later time. Behavioral data were collected by investigators who were blinded to participant treatment allocation by the methods explained above, which obviated any potential bias. The SIGH-D was collected only at bl and post appointments, while the MDI, GAD-7, and LSEQ were collected at all appointments. All behavioral assessments were collected in a private room by asking the participants about any changes, issues, or occurrences that might have happened since their previous appointment. For bl or post appointments, the SIGH-D was first administered, with participants then left alone to complete the MDI, GAD-7, and LSEQ. Mid-appointments were significantly shorter because the SIGH-D was not included. Once participants completed their self-report surveys, they were instructed to open the door; surveys were reviewed for completion and at bl and mid appointments; participants were given their blueberry or placebo packets, and at all appointments, their reimbursement check.

### 2.5. Blood, Serum, Plasma Collection, Storage, and Transport

At the start of the study, clinical phlebotomists were trained in blood collection and sample processing techniques specific to this study. After each appointment, phlebotomists collected whole blood into: Paxgene tubes, CRP tubes (provided by Labcorp or Pathgroup), BD vacutainer tubes with no additive (whole blood, serum), and BD vacutainer EDTA tubes (plasma). All tubes were then processed according to manufacturer instructions and standard sample processing protocols, and samples were either stored as is (i.e., Paxgene tubes), transferred to Labcorp or Pathgroup for analysis (CRP), or aliquoted into 2 mL Eppendorf tubes for storage and analysis. All samples other than CRP tubes were immediately stored on dry ice in a Yeti ice chest, and at the end of each week were transported back to LSU on dry ice by the researchers in a Yeti ice chest and transferred to a −80 °C freezer for long-term storage until analysis. Dry ice was replaced as needed and was obtained at the nearest available location.

### 2.6. C-Reactive Protein (CRP)

CRP is a routine lab test used by health clinics and was completed through contracted services at the clinics for these measurements. Originally, Labcorp (Burlington, NC, USA) was the contracted service provider, but was later changed to Pathgroup (Brentwood, TN, USA) midway through the study. The lower limits of detection differed between the two companies, which was normalized in data processing. Lab services and phlebotomists were familiar with collection protocols, which streamlined the process. Lab services picked up samples at the end of each business day for processing, results were faxed to the clinics, and clinic staff uploaded the results to the patients’ electronic medical records (EMRs). Results were then downloaded from the participants’ EMRs by study staff for analysis.

### 2.7. Serum Inflammatory Cytokine Panel

Serum samples were thawed on ice, vortexed, and three 50 µL aliquots were pipetted into 2 mL microcentrifuge tubes, and used for analysis or stored at −80 °C. Inflammatory cytokines of interest were assayed with the S-PLEX assay from Meso Scale Discovery Systems (Gaithersberg, MD, USA), based on its capability for multiplex assessment of several cytokines at a time in small sample volumes. We designed an S-PLEX assay that probed IL-6, TNF-α, IFN-y, IL-1β, and IL-10 for human serum samples.

First, specialized 96-well plates with electrodes (‘spots’) implanted into the bottom of each well were coated with 50 µL of a ‘coating’ solution (the contents of which were not disclosed by MSD as ‘proprietary information’), for 1 hour (h) at room temperature (RT), which increased the sensitivity and accuracy of the assays. Plates were washed 3 times with 1X phosphate-buffered saline + tris reagent (PBST) solution between each step. Next, calibration dilutions, samples, and the blocking solutions were prepared; 25 µL of the blocking solution was loaded into each well followed by 25 µL of either calibrators or sample and incubated for 1.5 h at RT, followed by three washes. Then, TURBO-Boost Antibody Solutions containing antibodies for IL-6, IL-1β, TNF-α, IFN-γ, and IL-10 were prepared, and 50 µL was added to each well and incubated at RT for 1 h. After the three washes, 50 µL of prepared ‘enhance’ solution was loaded in each well and incubated for 30 min (min) at RT to increase the sensitivity of the assay. Next, after three washes, 50 µL of a prepared ‘Turbo-Tag Detection Solution’ were dispensed in each well, and the plate was incubated for 1 h at 27 °C. Finally, after a gentle final three washes, 150 µL of read buffers were added to the plate, and the plate was placed in the MESO QuickPlex SQ 120 MM, which uses electrochemiluminescence to detect and quantify the protein bound to the spots which are each associated with an antibody of interest.

### 2.8. Metabolomics

Metabolomic data were obtained from serum samples. A total of 50 µL of frozen serum was packaged on dry ice and shipped overnight to the Biological and Small Molecule Mass Spectrometry Core at the University of Tennessee Knoxville (Knoxville, TN, USA), where all subsequent sample preparation, chemical analysis, data acquisition, and initial data analysis were performed. Samples were extracted with an acidic acetonitrile extraction protocol which uses 4:4:2 acetonitrile–methanol–water in 0.1 M formic acid as an extraction solvent. The extracts were dried under a steady stream of nitrogen and resuspended in 300 µL of ddH20. The resuspended samples were then analyzed using ultra-high performance liquid chromatography–high-resolution mass spectrometry. Water-soluble metabolites were separated using a reversed phase (C18) column (Synergi 2.6 µm Hydro RP column (100 mm × 2.1 mm, 100 Å; Phenomenex, Torrance, CA, USA)) and an UltiMate 3000 pump (Dionex, Sunnyvale, CA, USA) with an ion-pairing reagent over a 25 min gradient. The eluent was then introduced to the Exactive Plus Orbitrap MS (Thermo Scientific, San Jose, CA, USA) through electrospray ionization in negative mode (−ESI).

After data acquisition, the raw files generated by Thermo’s Xcalibur software Ver 4.1 were converted to open-source format, mzML, using msConvertGUI from the ProteoWizard v3.0.19254 software package. The mzML files were then uploaded into an open-source software package from Princeton University, Metabolomics Analysis and Visualization Engine (MAVEN). Using MAVEN 3.8.5, the extracted ion chromatograms were used to identify metabolites by comparing exact mass (+/−5 ppm) and retention time to an in-house standard library. The peak area of identified metabolites was integrated and exported into excel for further analysis.

### 2.9. Statistical Analysis

Statistical analyses were conducted and visuals were created with R programming language in R studio (The R Foundation, Indianapolis, IN, USA) R version 4.2.3 (15 March 2023 ucrt)—“Shortstop Beagle” Copyright (C) 2023. The R Foundation for Statistical Computing Group means derived from linear mixed-effects models for repeated measures (MEMRMs) and change score analysis are provided in Supplementary File S8.

For behavioral data, HDRS, GAD-7, and MDI yield raw numerical scores (discrete variables); however, each raw score corresponds with a categorical score of symptom severity, which were summarized as frequency and percentages at each timepoint for both blueberry and placebo. No normalization or transformations were performed because data were normally distributed. For each behavioral measure, efficacy was evaluated in two ways: (1) intention to treat analysis, which utilizes raw scores from all timepoints, and (2) per protocol/completers analysis utilizing change scores calculated from bl to post. Completers analysis was performed by first calculating change scores between bl and post timepoints (post-baseline), which were pooled between BB-first and placebo-first groups, and analyzed using a paired *t*-test. The “full” or intent to treat analysis was conducted using MEMRMs estimated via restricted maximum likelihood (REML). Raw scores for each behavioral measure (HDRS, GAD-7, and MDI) served as the response variable, with treatment (“tx”), “period” (“arm”), sequence (“bb_first”), appointment (“appt_cat”), and treatment-by-appointment interactions modeled as fixed effects, and subject (“de_id”) included as a random effect. Models were fitted with the lmer function in the lme4 package. Least-square means, standard errors, and confidence intervals were calculated using the lsmeans and confint functions in the Lmertest package. Model formulas, output, and evaluation criteria for each response variable are provided in Supplementary File S8 (HDRS, GAD-7, and MDI). Behavior scripts are available in Supplementary File S9.

For inflammatory cytokine markers IL-1β, IL-6, TNF-α, IFN-γ, and IL-10, concentrations (fg/mL) were log-transformed to normalize distributions and outliers that were greater than 3 standard deviations from the mean were excluded from analysis. The simplest mixed-effects models for repeated measures (MEMRMs) that effectively represented the study design were selected and estimated using restricted maximum likelihood (REML). No unnecessary fixed effects or covariates were included. The response variable in each model was cytokine concentration (continuous variable); each cytokine was analyzed in a separate model. Fixed effects included appointment (categorical variable—bl, mid, post), treatment (categorical variable—blueberry or placebo), appointment x treatment interaction, period (categorical—Period 1 or Period 2), and sequence (categorical: blueberry first or placebo first), with the subject as a random effect. Models were fitted using the lmer function with variables defined as follows: bb_first = order; appt_cat = appointment; and de_id = subject identifier. Supplementary File S8 provides detailed model formulas, outputs, and evaluation. Biomarker analysis scripts are provided in Supplementary File S10. Multiple linear regression was employed to examine correlations between inflammatory markers and behavioral measures.

For metabolomic analysis, processed data were analyzed with MetaboAnalyst 5.0 (MetaboAnalyst, 2024), a free, online, open-source metabolomic statistical analysis program. Raw peak intensities were entered into the statistical analysis [one factor] pipeline with the following parameters defined. Outliers were removed from data via the Interquartile Range (IQR) variance filter, set to 5%. After outliers were removed no normalization was selected (since data were previously normalized); however, Log10 transformation and Pareto scaling were used to further transform the data and normalize distributions. The results of the data transformation and scaling parameters are visualized in Supplementary File S11.

Data were first evaluated for period effects to determine if blueberry and placebo conditions between Period 1 and 2 could be pooled and analyzed together. Results indicated a significant period effect, with 65 out of 80 metabolites differing significantly (FDR < 0.05) between Period 1 and Period 2, and principal component analysis (PCA) revealed clear separation between the two periods (see Section 3).

MetaboAnalyst software can only perform one-factor analysis and is unable to implement mixed-effects models to account for period effects. Therefore, analyses could not be carried out directly between blueberry and placebo conditions and only within each condition between timepoints (i.e., bl vs. mid, bl vs. post). Furthermore, because period effects were observed and could not be addressed statistically in MetaboAnalyst, only Period 1 data were included in our metabolomic analysis. Changes were evaluated via paired analyses for the (1) blueberry-first group from baseline (bl) to mid, (2) blueberry-first group from bl to post, (3) placebo-first group from bl to mid, and (4) placebo-first group from bl to post. Since this aspect of the study was exploratory and the use of only Period 1 significantly decreased statistical power, we opted to use detection parameters of 1.25 fold change (FC), which would indicate a 25% change, and an FDR < 0.1 which is less conservative than FDR < 0.05 but more informative than a raw *p* < 0.1 or 0.05. The relevant literature reports significance ranging from raw *p* < 0.1 to FDR < 0.05; FDR are also referred to as adjusted *p*-values [40,41,42]. Results are presented such that the comparisons between the two conditions are facilitated, i.e., blueberry and placebo, at their respective changes at the same timepoint. The data were organized to depict a visualization of metabolites that changed significantly from bl to mid timepoints in blueberry and placebo via volcano plots with labels of metabolites that demonstrated at least a 1.25 FC and an FDR < 0.1. Plots were constructed showing the number of metabolites that had at least a 1.25 FC alone, and metabolites with FDR < 0.1 alone. Statistics for metabolites in each analysis are summarized in Supplementary File S12.

### 2.10. Post Study Participant Information Session

All participants were contacted after the study by phone and informed of the overall study’s outcomes, their personal outcomes, and how they compared to the general outcomes of the study. Questions from the participants were also answered at this time. Finally, the extreme value of their time and participation was communicated to participants in full by the investigators.

## 3. Results

### 3.1. Study Population and Recruitment

Participants for this study were originally recruited from five Rural Health Clinics (RHCs) in Louisiana and through local radio advertisements and community events. After a total of 6 months of recruitment, participants were only elicited from three clinics in Cottonport, Marksville, and Elizabeth (all cities in Louisiana). However, the two patients recruited at the Elizabeth clinic dropped out of the study, narrowing the scope to the two remaining clinics, i.e., Cottonport and Marksville. The final study population was comprised primarily (~80%) of middle-aged females. Demographic information including age, sex, and psychiatric medication can be found in Supplementary File S13.

### 3.2. Behavioral Assessments

**Figure 2:**
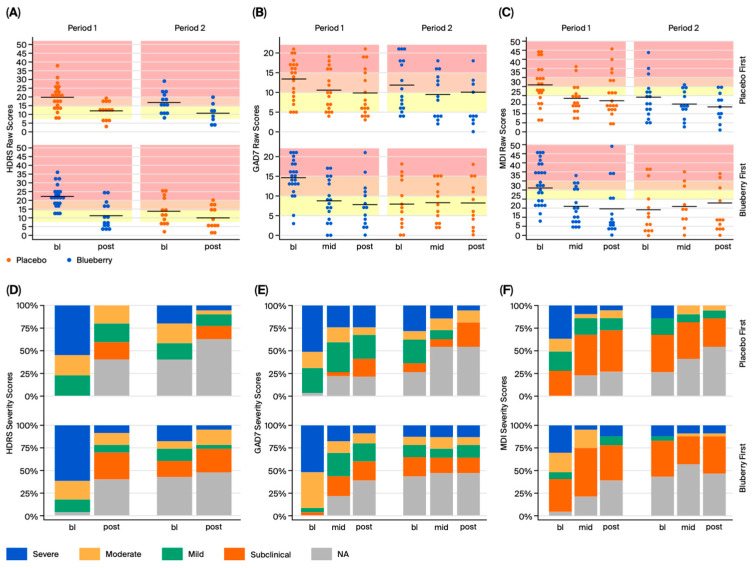
Individual raw scores (top panel) and frequencies (bottom panel) for behavioral tests. HDRS (**A**,**D**), GAD-7 (**B**,**E**), and MDI (**C**,**F**) results are illustrated in the respective panels. Raw scores are presented with dot plots, in which the black lines represent the group means; the background colors in (**A**–**C**) represent the symptom severity (dark pink = severe, light orange = moderate, yellow = mild, and white = subclinical). Frequencies are presented as percentages for ease of comparison between groups.

We employed several standardized approaches to measure behavioral outcomes: Hamilton Depression Rating Scale (HDRS), the Generalized Anxiety Disorder 7-Item Questionnaire (GAD-7), and the Major Depression Inventory (MDI) (Figure 2; Supplementary File S14). The minimal clinically important difference (MCID) on each of these assessments was used to gauge the clinical relevance of the observed effects. The MCID represents the smallest positive change in a therapeutic outcome that is considered worthwhile by a patient [43,44]. The MCID for the HDRS is 3–8 points, [45] while the MCID for the GAD-7 is 4 points [46]. No MCID for the MDI has been established.

**Figure 3:**
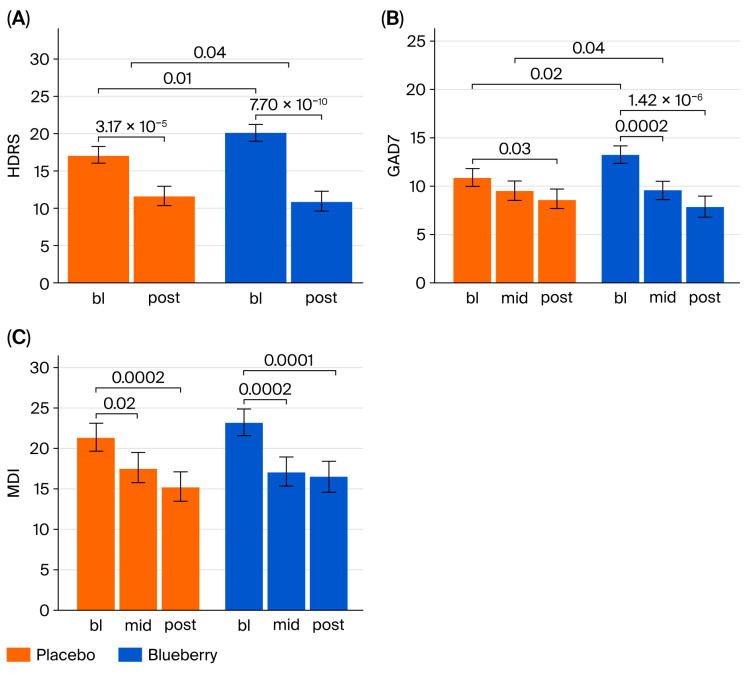
Group means derived from MERMS of HDRS (**A**), GAD-7 (**B**), and MDI (**C**). In (**A**,**B**), the top bar with two downward lines indicates the *p*-value of the comparison between the changes from bl to post in placebo versus blueberry conditions (add the stats details here). All other bars represent the *p*-values of their respective comparisons, for example, placebo bl to blueberry bl; bl to mid or bl to post within condition. Significance was defined as *p* < 0.05.

**Figure 4:**
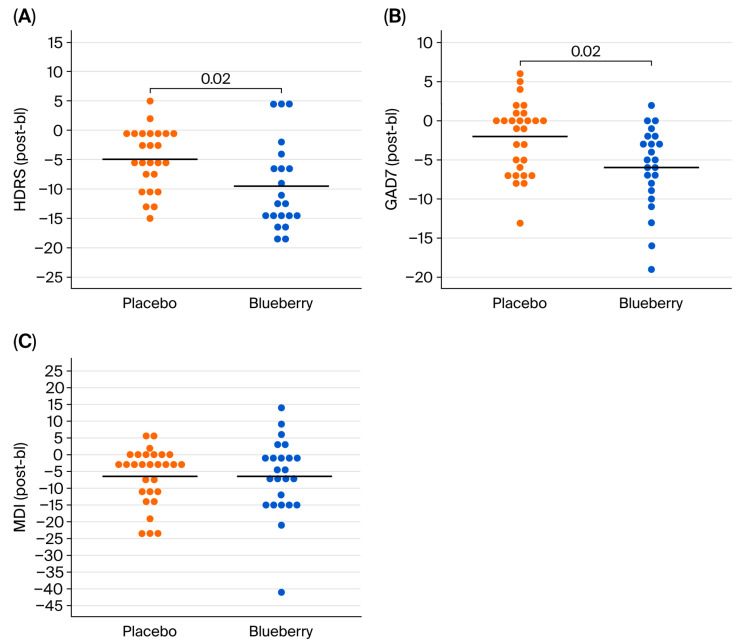
Change scores for placebo and blueberry conditions. HDRS (**A**), GAD-7 (**B**), and MDI (**C**) results are depicted on the respective panels. Each point represents an individual’s change score, calculated by subtracting bl from post for the respective conditions; black bars are group means. Lower scores indicate greater symptom improvement.

In our study, we found that blueberries were significantly more effective than placebo in decreasing the symptoms of depression and anxiety measured via the HDRS and GAD-7 from bl to post timepoints, but not between the bl and mid timepoints (Figure 3 and Figure 4). Specifically, MEMRMs analysis revealed significant effects of blueberry treatment compared to placebo on symptoms of depression (HDRS: 3.686 ± 1.791, t = 2.058, (Pr(>|t|) = 0.0431) and anxiety (GAD-7: 3.134 ± 1.476, t = 2.123, (Pr(>|t|) = 0.035) from bl to post measures, but not for the MDI (0.564 ± 2.365, t = 0.238, (Pr(>|t|) = 0.811). No significant effects were observed between bl and mid for the GAD-7 (2.336 ± 1.406, t = 1.662, (Pr(>|t|) = 0.0987), or for the MDI (2.31 ± 2.295, t = 1.006, (Pr(>|t|) = 0.316) (Figure 3; Supplementary Files S8 and S9).

The blueberry’s mean difference was 3.69 points lower than the placebo’s mean differences from bl to post timepoints and was within the range of the minimal clinically important difference for HDRS (3–8 points), but did not meet the criteria in the case of the GAD-7 (4 points), with blueberry mean differences being only 3.13 points lower than placebo mean differences from bl to post timepoints. Baseline values for all behavioral measures between Period 1 and Period 2 exhibited period effects, which are common in crossover studies. Modeling data also indicate significant differences between placebo and blueberry conditions when baseline values are pooled between Period 1 and Period 2 (Figure 2), but not for either period individually, likely due to increased statistical power when combining Period 1 and Period 2 values. We emphasize that no significant differences were observed between baselines in the placebo and blueberry conditions within Period 1 (HDRS *p* = 0.3, GAD7 *p* = 0.4, and MDI *p* = 1) or Period 2 (HDRS *p* = 0.3, GAD7 *p* = 0.1, and MDI *p* = 0.3) individually. Mixed-effects models were employed here due to their ability to statistically isolate treatment effects by 1) including period as a fixed effect in the model and 2) adjusting for random effects including individual variability across the entire study’s duration. Mixed-effect modeling is the preferred analysis for longitudinal crossover studies with repeated measures.

In addition, we conducted a “completers” analysis to compare the change scores from bl to post timepoints between blueberry and placebo conditions via a paired Student’s *t*-test for each behavioral measure (HDRS, GAD-7, and MDI) (Figure 4). This completers analysis of change scores supported the effects observed in our modeling data, demonstrating that blueberry was significantly more effective at reducing symptoms from bl to post timepoints on the HDRS (*t*(19) = 2.57, *p* = 0.02, Cohen’s d = 0.57 (moderate effect)) and the GAD-7 (*t*(20) = 2.46, *p* = 0.02, Cohen’s d = 0.54 (moderate effect)), but not the MDI (*t*(21) = 0.96, *p* = 0.35, Cohen’s d = 0.20 (small effect)). The mean changes in blueberry compared to placebo conditions were slightly larger according to the completers analysis than the modeling for the HDRS (4.64 points) and the GAD-7 (3.67 points), although still not reaching the clinically important difference threshold for the GAD-7.

### 3.3. C-Reactive Protein and Inflammatory Cytokines

**Figure 5:**
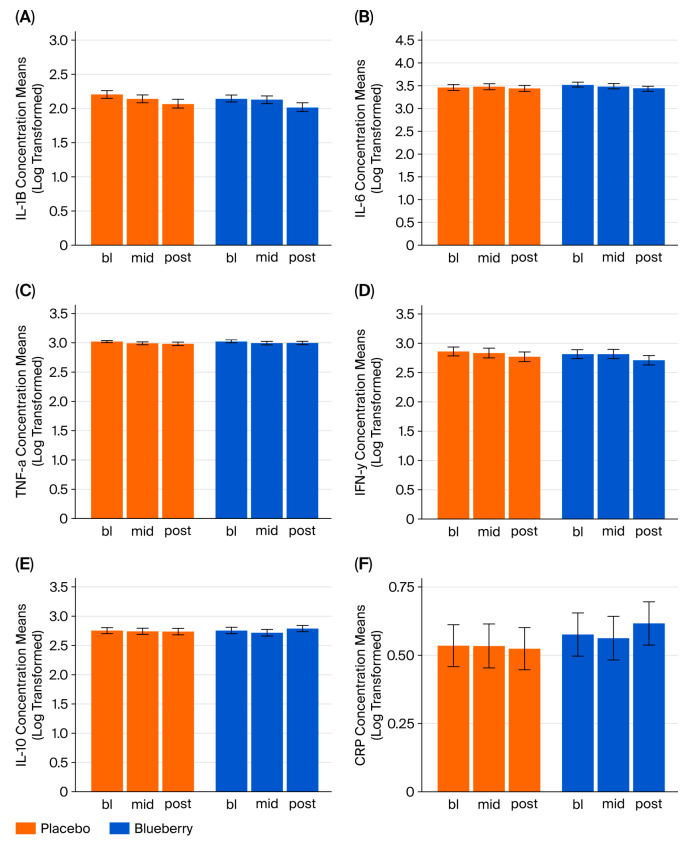
Serum pro and anti-inflammatory cytokines and *C*-reactive protein. Group means derived from MERMS of IL-1B (**A**), IL-6 (**B**), TNF-a (**C**), IFN-y (**D**), IL-10 (**E**), and CRP (**F**). No significant differences were observed for CRP or any cytokine.

Pro- and anti-inflammatory cytokine and *C*-reactive protein levels were assessed in aggregate data across both periods. We compared the serum concentrations of *C*-reactive protein (CRP) and a panel of pro- (IL-1β, IL-6, TNF-α, and IFN-γ) and anti-inflammatory cytokines (IL-10) between blueberry and placebo conditions. The mixed-effects models revealed no significant effects of time or treatment for any inflammatory cytokines and no period or sequence effects (Figure 5, See Supplementary File S8 for detailed statistics). However, we did observe moderate positive correlations between HDRS scores and IL-1β and IL-6 values at the post-treatment timepoint in the blueberry condition only and between HDRS scores and IFN-γ in the post-treatment timepoint in the placebo condition (Supplementary File S15). Spearman correlation coefficients for the cytokines against each behavioral measure are summarized in Supplementary File S15. All scripts are in Supplementary File S10.

### 3.4. Global Metabolomics

We evaluated if changes in the global metabolome differed within each group, and whether any of those changes were correlated to the behavioral outcomes. Overall, 80 metabolites were detected in our plasma samples based on exact mass and retention time (complete list: Supplementary File S12).

**Figure 6:**
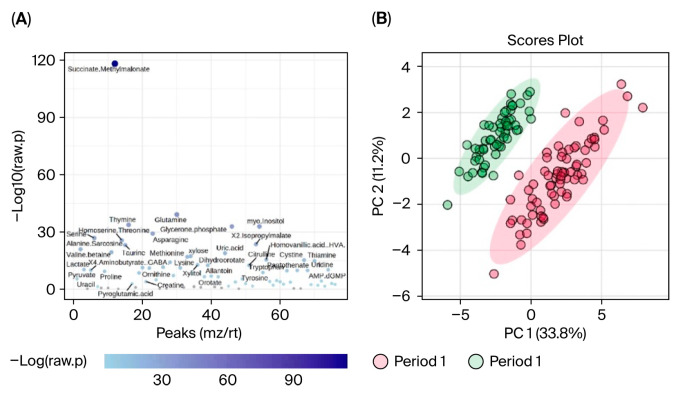
Comparison of metabolites in Period 1 and Period 2. (**A**) *t*-test plots with labeled metabolites indicate that 65 (out of 80) metabolites significantly differed (FDR < 0.05) between Period 1 and Period 2. (**B**) PCA plots indicate clear separation between Period 1 (red) and Period 2 (green), indicating period effects.

Period effects were observed in these metabolites between Period 1 and Period 2. Specifically, 65 (out of 80) metabolites differed significantly (FDR < 0.05) between Period 1 and Period 2, and principal component analysis (PCA) revealed clear separation between the two periods (Figure 6). Due to the software limitations of MetaboAnalyist, which can only perform single factor analysis and cannot employ mixed models that can control for period effects, as we implemented for the behavior results, we only analyzed metabolomic changes in Period 1 (see Section 2).

**Figure 7:**
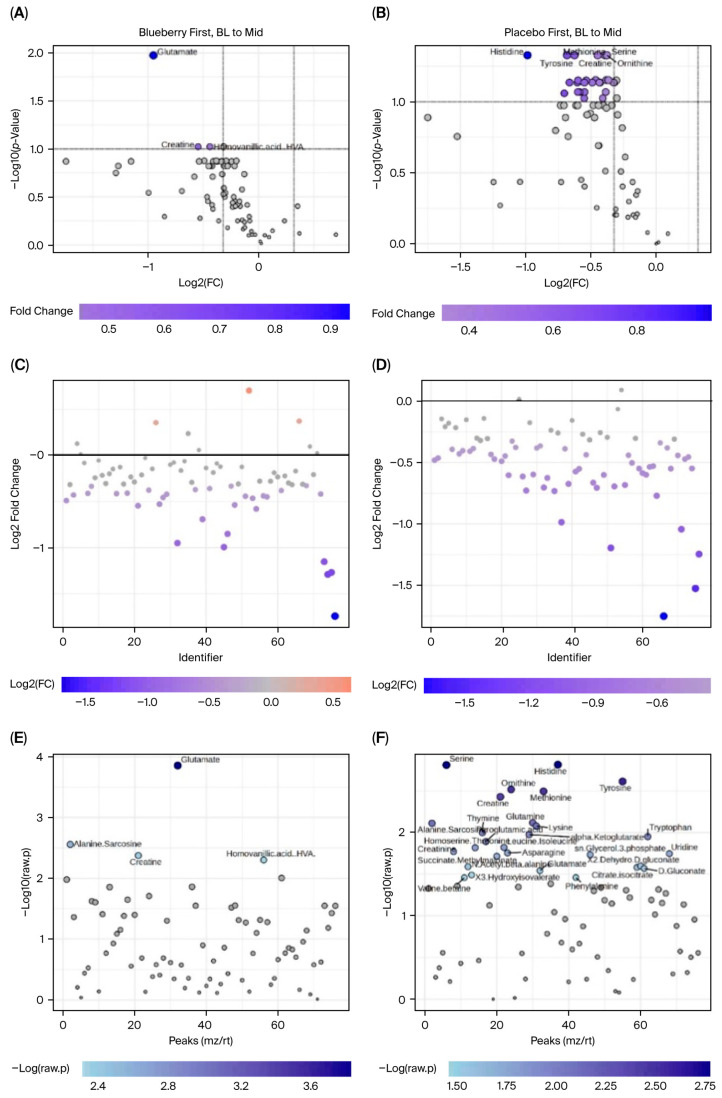
Metabolite alterations at the middle timepoint. Blueberry plots (**left panels**) and placebo plots (**right panels**). Volcano plots (**A**,**B**) visualize and label metabolites that demonstrated an FC > 1.25 and an FDR < 0.1 from bl to mid; FC plots (**C**,**D**) show the number of metabolites that had an FC > 1.25 from bl to mid; and *t*-test plots (**E**,**F**) visualize and label the metabolites that differed significantly (FDR < 0.1) from bl to mid.

**Figure 8:**
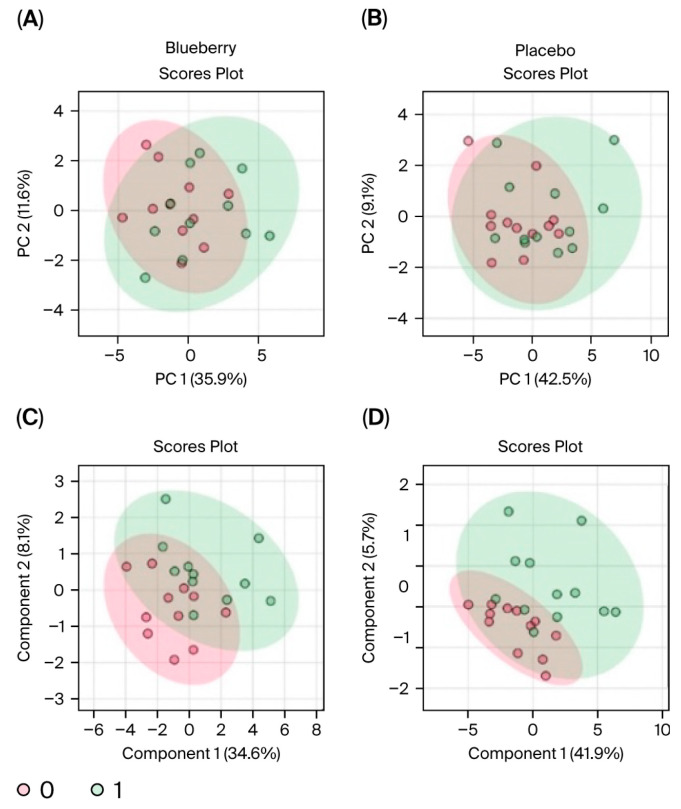
PCA and PLS-DA comparison of baseline and middle timepoints. PCA (**A**,**B**) and PLS-DA (**C**,**D**) plots for blueberry (**left panels**) and placebo (**right panels**). Baseline (bl) samples are indicated in red and middle samples are indicated in green. In these plots, separation is indicated by little to no overlap of the different-colored encompassing ovals.

**Figure 9:**
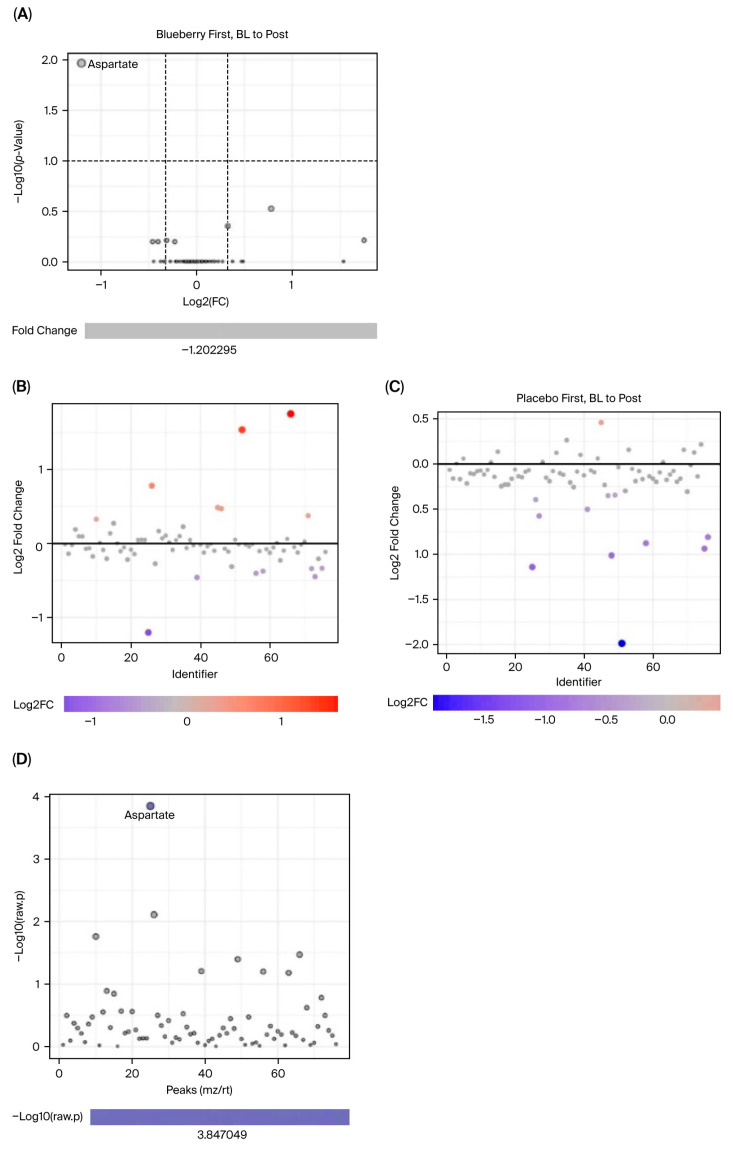
Metabolite alterations between baseline and the post timepoint. Blueberry plots are on the left panel and placebo plots are on the right. Volcano plots (**A**) visualize and label metabolites that demonstrated an FC > 1.25 and an FDR < 0.1 from bl to post in the blueberry group; FC plots (**B**,**C**) show the number of metabolites that had an FC > 1.25 from bl to post; and *t*-test plots (**D**) visualize label the metabolites that differed significantly (FDR < 0.1) from bl to post.

**Figure 10:**
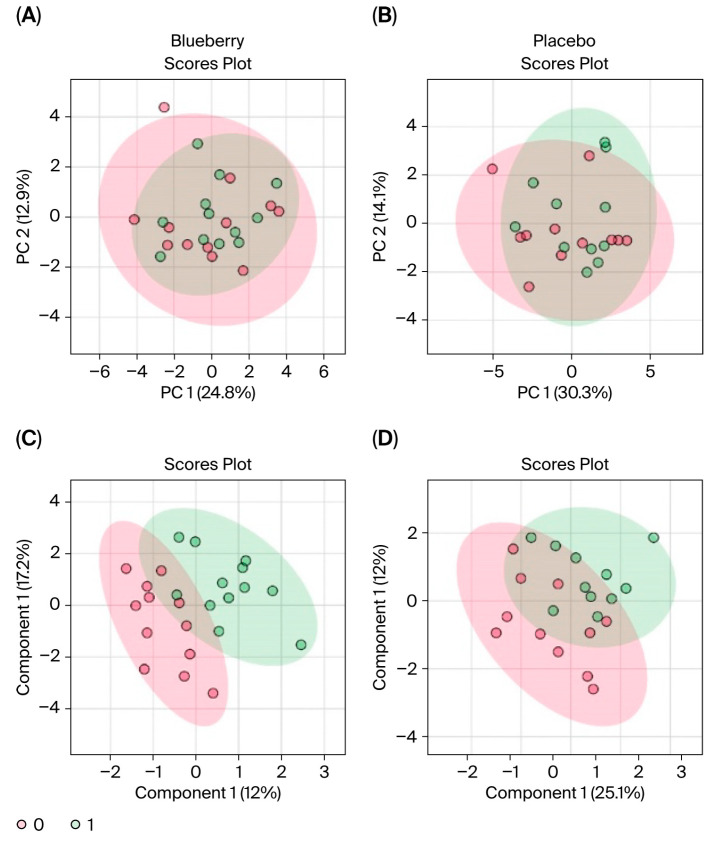
PCA and PLS-DA comparison of baseline and post timepoints. PCA (**A**,**B**) and PLS-DA (**C**,**D**) plots for blueberry (**left panels**) and placebo (**right panels**) between bl and post; bl samples are indicated in red and post samples are indicated in green. In these plots, separation is indicated by little to no overlap of the different-colored encompassing ovals. PCA: primary components analysis; PLS-DA: partial least squares-discriminant analysis; bl: baseline.

Interestingly, we observed more significant changes in metabolite concentrations between the bl vs. mid time points (Figure 7 and Figure 8) compared to bl vs. post (Figure 9 and Figure 10). Among the changes, aspartate only reached significance in the blueberry condition; however, aspartate had a virtually identical FC in both the blueberry and placebo conditions from bl to post, despite not reaching the <0.1 FDR in the placebo condition. However, no metabolite reached significance via both parameters in the placebo condition from the bl to post timepoints. Finally, the blueberry condition exhibited some global separation from the placebo condition in the PLS-DA analysis (Figure 10C,D), but not the PCA (Figure 10A,B). Overall, these data are indicative of potential changes to the global metabolome associated with blueberry treatment or a color- and flavor-matched placebo, but additional studies in larger populations are required to confirm our results.

## 4. Discussion

### 4.1. Overall Outcomes

Overall, our behavioral results suggest a significant effect of 12 weeks of 24 g/day blueberry supplementation on clinical symptoms of depression and anxiety compared to a flavor- and color-matched placebo in participants from rural Louisiana. However, significant effects were not observed at 6 weeks. We also did not observe any treatment effects on pro- and anti-inflammatory cytokines or CRP, or correlations between biomarkers and symptoms. Interestingly, exploratory global metabolomics identified potential metabolite changes in the blueberry group. Importantly, this pilot study is the first of its kind conducted in the unique setting of rural health clinics, and these findings require further investigation and replication in larger populations to draw strong and generalizable conclusions. Within this limited context, our behavioral results support the hypothesis that blueberry supplementation is more effective than placebo in reducing symptoms of anxiety and depression.

The significant reductions in depression and anxiety symptoms of this study at 12 weeks are novel. The HDRS results (a 3.69 point improvement compared to placebo) were clinically significant according established minimal clinically significant difference (MCSD) criteria, and the GAD-7 score (3.13 point improvement compared to placebo) nearly reached clinical significance and was higher than that observed in metanalyses performed on SSRIs [47]. As mentioned, we did not observe significance at the 6-week timepoint. Interestingly, Velichkov et al. (2024) also did not observe significant reductions in depression and anxiety symptoms at the end of their 6 week study, although they did observe improved affect and executive function [48], suggesting that a longer supplementation period might be required to achieve clinically relevant changes for depression and anxiety symptoms. Direct comparison to more recent metanalyses on SSRIs [49] or psylocibin depression studies [50] is not feasible, because the examined primary endpoints differ. The Montgomery–Asberg Depression Rating Scale (MADRS) has replaced the HDRS as the preferred depression scale in research due to its increased sensitivity to treatment effects [51]. Nonetheless, the moderate effect sizes we observed for blueberry compared to placebo in the change score analysis for the HDRS (Cohen’s d = 0.57) and GAD-7 (Cohen’s d = 0.54) are larger than the modest effects observed in the metanalysis conducted on SSRIs [52,53,54,55]. Importantly, our study did not require that study participants stop or alter their current medications, suggesting that blueberry supplementation may improve depressive symptoms in patients already undergoing standard of care for MDD and GAD. However, given our small sample and unique population, these data must be replicated in larger and more diverse populations to draw strong and generalizable conclusions.

A general concern with all studies that employ behavioral measures is the potential confound for experimenter influence on these outcomes. We emphasize that our study was rigorously controlled through a double-blind, placebo-controlled design, as detailed fully in the Methods section. Potential biases in group assignments were minimized through randomization, and all participant-facing study staff were blinded to group assignment until after the end of the study. Finally, our statistical analysis of the data followed the gold standard for analyzing the significance of the behavioral outcomes assessed (See Section 2 and Supplementary File S8).

Taken together, the clinically relevant changes and statistically significant effect sizes observed with blueberry treatment compared to placebo in this pilot study warrant further and more comprehensive investigations in larger studies in more diverse populations. For this pilot study, inclusion and exclusion criteria were intentionally kept as broad as possible to assess whether effects could be measured in a naturalistic, unbiased setting and clinical population. Future studies conducted in different populations or urban settings, which include larger samples, are more tightly controlled, and include the MADRS as an endpoint, are required to confirm or generalize these results and compare them to existing or experimental treatments. For example, the participants in our study were predominantly female and most participants were on psychotropic medications. Therefore, assessing whether blueberries affect symptoms of MDD or GAD in men, and in specific populations who are not on pharmacotherapies, or those who are exclusively on one class of pharmacotherapy (e.g., SSRIs) is necessary to generalize these results, and to determine if blueberries positively augment existing pharmacotherapies. Further, parsing out if blueberry effects symptoms of MDD or GAD alone or in combination is also required to develop a more nuanced understanding of these effects. Longitudinal studies to evaluate the long-term effects past the 12-week treatment period and the durability of these effects after ceasing blueberry treatment are also required to fully understand these effects. Therefore, due to the notable limitations discussed, the ultimate effects of blueberry treatment for symptoms of depression and anxiety warrant deeper research to solidify their potential role as a nutraceutical for these conditions.

### 4.2. Behavioral Measures

Three psychometric measures were implemented in this study. The SIGH-D was chosen because of its test–retest and inter-rater reliability [39]. The MDI and GAD-7 were selected as self-report measures to complement the researcher-administered SIGH-D interviews and to provide data for mid-timepoints [56,57]. The HDRS is a more sensitive and reliable measure of depression than the self-reported MDI, especially for milder symptoms. Thus, the lack of significant results on the MDI is not compelling enough to reject the hypothesis. A self-report measure other than the MDI that is more highly correlated to the HDRS and better at capturing symptoms across the entire range of depressive symptoms may have better captured treatment effects. Overall, each behavioral test has both advantages and disadvantages, as discussed below.

The Hamilton Depression Rating Scale (HDRS) [58] was employed in this study because it is the most common rating scale used in the diagnosis and study of depression, often called the “gold standard”. In 1988, the Structured Interview for the Hamilton Depression Rating Scale (SIGH-D) was created, which increased inter-rater and test–retest reliability [39]. In this study, two interviewers performed all clinical interviews across repeated timepoints; thus, we chose this structured interview to standardize test administration, which enabled diagnosis and severity assessment based on both Diagnostic and Statistical Manual (DSM) and the International Classification of Diseases, Tenth Edition (ICD-10) criteria. A limitation of these interviews is their time commitment (~30 min to 1 h), particularly compared to self-report surveys that can be completed in 5–10 min.

The Generalized Anxiety Disorder 7-Item Questionnaire (GAD-7) is a self-reported questionnaire published in 2006 [56] that was developed to address the issue of lengthy clinical interviews in the assessment of GAD. The GAD-7 is reliable, sensitive, and exhibits high construct, factorial, and procedural validity [56]. This measure was employed due to the highly reduced time commitment compared to a clinical interview, allowing for the measurement of anxiety at all timepoints.

The Major Depression Inventory (MDI) was employed for a similar reason to the GAD-7. The MDI is a brief 10-item, self-report questionnaire developed by the World Health Organization (WHO) to assess moderate to severe depression according to the diagnostic criteria of both the ICD-10 and the DSM [57]. In addition, the MDI is diagnostic, allowing for the confirmation of a participant’s diagnosis at screening, without performing a clinical interview. The MDI demonstrates good specificity and sensitivity for moderate to severe symptoms but is poor for mild symptoms. It is thus unlikely to measure mild depressive symptoms or changes sensitively or accurately. The MDI is less frequently used in clinical settings and research for this reason, and most clinical trials now implement the 21-item, self-reported Beck Depression Inventory (BDI). However, given our need to confirm diagnosis during screening, we implemented the MDI. If needed, future studies may benefit from using the MDI for screening purposes but implement the BDI for longitudinal self-report measures of depressive symptoms.

### 4.3. CRP and Inflammatory Cytokines

This study was motivated by our prior studies in a preclinical stress model, which demonstrated that the human equivalent of 2 cups of highbush blueberry concentrate per day produced anxiolytic effects and normalized stress-induced changes in neurotransmitters, inflammation, and ROS production in a rat model of post-traumatic stress disorder (PTSD)-like stress [26,59]. As such, we sought to assess whether similar physiological effects could be observed in this preliminary clinical study. However, the inflammatory cytokine panel and CRP results led us to reject the hypothesis that behavioral effects were related to changes in systemic inflammatory markers in this population. Data from this pilot study suggest that systemic levels of inflammatory markers are not a likely driver of the observed treatment effects, but other possibilities regarding the immunomodulatory effects of blueberries remain.

Several methodological caveats may be relevant to these null findings. First, it is important to consider that group designations were randomized based on CRP levels; however, we did not recruit in a way that selected those with only inflammation-related depression and/or anxiety. Anti-inflammatory treatments could reduce inflammation-related depression in those with high circulating levels of cytokines, but this was unlikely to be the case in the current study given the relatively low levels of CRP observed here. Circulating peripheral markers also may not be sufficient to capture changes in inflammation that might occur in the central nervous system (CNS) or in response to acute stress. For example, blueberries could produce therapeutic effects by altering inflammatory markers in the brain, as observed in our animal models [26,59]. Blueberries could also impact the stimulated expression of inflammatory markers while not impacting basal levels. Stimulated pro-inflammatory cytokine expression can be measured in primary cells collected from participants using ex vivo stimulation methods paired with cytometry by time of flight (CyToF).

Prior studies have addressed depression associated with neurodegenerative [60] (NCT04932434) and other inflammation-associated disorders, like cardiovascular disease [61,62] (NCT06170255). Blueberries may exhibit a measurable effect on systemic cytokines in the context of preexisting elevated inflammation, and these effects might correlate to reduced anxiety and depression symptoms. Alternatively, peripheral inflammation may not be a direct contributor to the symptoms of MDD and GAD, even in those with elevated inflammation. However, inflammation in the central nervous system (CNS) may still be an important driver of disease. If it is true, employing the more invasive techniques of cerebral spinal fluid (CSF) sampling or positron emission tomography (PET) imaging of inflammatory markers, like the translocator protein (TSPO), might reveal this association, although the benefits would need to outweigh the risks of these procedures.

Alternatively, blueberries might not exert anti-inflammatory effects via the reduction in circulating inflammatory cytokines [63]. In addition, prior results from animal studies may be due to their differences in intrinsic physiology compared to humans [26,59,64,65,66]. While there is ample evidence that blueberries have beneficial effects in conditions that are associated with elevated inflammatory states [67], we did not observe any effects in this current study of participants with relatively low systemic levels of inflammatory markers. Given the methodological considerations above, an anti-inflammatory effect cannot be completely ruled out, but these data suggest that alternative non-immune mechanisms may have contributed to these results.

To determine the therapeutic effects of blueberries on symptoms of anxiety and depression, future studies might focus instead on inflammation-associated anxiety or depression, since there is evidence that high baseline levels of peripheral inflammatory markers might be correlated to increases in symptom severity [68] or resistance [69] to particular treatments. This could be achieved by screening for those with higher levels of CRP or circulating inflammatory cytokines like IL-6 or IL-1β, or by recruiting those with depression and/or anxiety with other comorbidities that are themselves associated with high levels of circulating inflammation, like heart disease, diabetes, or neurodegenerative disorders. Alternatively, assessing other targets like NF-κB, other metrics of pro- and anti-inflammatory activity [70,71], brain levels of inflammatory biomarkers such as TSPO, inflammatory markers in CSF, or stimulated cytokine expression, may help to determine if the immune effects of blueberries play a role in their therapeutic effects in MDD and GAD. Ultimately the FDA requires demonstration of the safety and efficacy of experimental therapeutics, while understanding the mechanisms underlying these effects are helpful, but not required, for approval [72].

### 4.4. Global Metabolomics

Overall, the global metabolomics data from this pilot study suggested potential changes following blueberry supplementation. However, these results are also highly tentative. As noted above, in an exploratory study of this nature, methodological limitations constrain any absolute conclusions. Our measures were impacted by the unknown daily dietary intake over the duration of this study, and collections were not obtained from fasted blood samples. As a result, assigning any observed changes due solely to blueberry supplementation is currently unwarranted.

Dietary intake is common in metabolomic blueberry studies in the literature [34,73], but it is surprisingly not a standard in metabolomic studies related to mental health conditions, although blood draws are typically fasted and the timing of blood draws are controlled [74,75,76], see Caspani et al. (2021). Unfortunately, because the employed metabolomic methods extracted and analyzed mainly water-soluble metabolites, the results are not directly comparable to prior metabolomic studies for mental health, which analyzed mainly lipophilic molecules. Further, we lost significant statistical power due to the limitation of MetaboAnalyst to one-factor analysis and the reported period effects, which restricted our analysis to only Period 1. As such, our metabolomic data, though novel for mental health studies, necessitate further studies from comparable, but larger samples.

As such, we suggest future directions that would be preferable to evaluate the role of blueberry supplementation on the human metabolome in depression and anxiety: (1) targeted metabolomic techniques to evaluate metabolites specifically related to blueberries, which can also be used to measure compliance [77], (2) analyze lipid panels and related molecules with these metabolomic techniques, and (3) control experimental conditions, including dietary intake analysis/control, shorter durations, and highly controlled time-course studies. These additional considerations may yield more informative and concrete data regarding metabolomic alterations following blueberry supplementation.

### 4.5. Methodological Consideration

To our knowledge, this is the first RCT to be conducted using rural health centers as a study site. Although implementation of the study in this unique setting posed distinct challenges not present in urban clinical settings (see Section 4.6), we implemented several de rigeur approaches for maintaining methodological integrity and rigor in the study’s design, implementation, and analysis. In particular, we note that several steps were taken to ensure that the study was free from selection bias.

To limit the influence of potential selection bias on the study’s outcomes, we implemented the following. First, we required that participants had an existing diagnosis of MDD or GAD. Second, we confirmed that participants had a current MDD diagnosis measured by the MDI, or current clinically relevant GAD symptoms measured by the GAD-7. The MDI was selected because it is a validated diagnostic instrument for MDD; therefore, we were certain that these participants were diagnosed with MDD and had MDD at the time of recruitment. In addition, the GAD-7 (not a diagnostic instrument, but has 89% sensitivity/82% selectivity for GAD, see Kroenke et al. (2007) and Supplementary File S14) confirmed that participants had significant GAD symptoms at recruitment [78].

We implemented a matched pseudo-randomized design for group assignments (see Section 2.3). The matching procedure ensured that each group (BB-first or placebo-first) was balanced on four factors: age, sex, baseline MDI, and baseline CRP (see Section 2). We also confirmed that groups did not significantly differ on any of these four factors after randomization. Therefore, the groups were balanced and randomized based on reasonable and relevant criteria, and selection bias did not influence group assignment.

No baseline differences in any primary measure were observed in either period alone. However, baseline differences were observed for the HAMD and GAD-7 when combining data across periods, but these differences were controlled statistically and did not influence the study’s outcomes. Period effects were observed after the four-week crossover period such that both groups were lower in Period 2 than Period 1, as is common in crossover trials, but no group-specific bias was observed. Furthermore, the crossover design allowed each individual participant to serve as their own control, reducing the potential impact of between-group baseline differences on the study’s outcomes. Thus, the observed baseline differences that emerged after combining the two phases are likely a random effect, and not a result of our assignment methods or any form of selection bias.

Any potential sources of bias were controlled for using mixed-effects models, including the observed differences in baseline HAMD and GAD-7 scores after combining data across both periods (see Section 2.9, Supplementary Files S8–S10). For example, our models included subject-level random effects, which absorb any between-subject baseline differences, anchoring each of the participants’ outcomes around their own mean. Period was also included as a fixed effect in our models, preventing period effects from biasing the treatment estimate. Therefore, we conclude that the observed between-group effects are robust to the baseline differences that emerged after combining data across both periods.

### 4.6. Rural Health Settings

This pilot study also demonstrates the feasibility of implementing a long-term study with behavioral and physiological endpoints in a Rural Health Clinic (RHC) setting [11]. This study provides a unique contribution to the field, because, to our knowledge, it is only the third clinic trial and, to our knowledge, the first RCT trial ever conducted in an RHC setting [8,9]. Our study sought to address the current barriers and the neglect of rural populations in health research studies [11]. However, tradeoffs are required when conducting research in an RHC setting compared to traditional and well-equipped research facilities, which require optimization and streamlining. In particular, limited study personnel is a major challenge to these RHC studies, which may require that investigators or clinical research coordinators assume several duties including recruitment, participant management, data collection, database management, and sample transport.

Using RHCs as a study site for rural health research is innovative [11]. For rural participants, traveling to a study site in an urban or suburban area is a major barrier to participation in health research due to work schedules (and lack of flexibility), childcare, or lack of reliable transportation; traveling 1–3 h to participate in a study entails at least a half or full day off work. Furthermore, because travel is often a major burden to rural participants, investigators might ethically be required or encouraged to reimburse participants for travel costs, leading to potentially prohibitively expensive study budgets. Having study sites in proximity to rural participants, particularly at sites in which they already receive health care, is an excellent way to mitigate this barrier to participation and repair the neglect of rural populations in health research. In addition, we found that recruiting a broad demographic population in rural areas is especially challenging, particularly for studies involving mental health conditions, which may be viewed as a taboo subject.

Distrust in the medical system is also growing among those who live in rural areas, which existed previously but was highlighted and exacerbated by the COVID-19 pandemic [79,80]. Lister and Joudrey (2023) offer an excellent perspective on this topic [81]. It is particularly important to understand that this mistrust is not completely unfounded; there are complex and valid sociocultural reasons underlying it. To improve health-related outcomes in these populations, health professionals and researchers must find understanding, balance, and have a willingness to improve communication and relationships in these populations. This mistrust is accompanied by growing desires for more personal control and natural approaches to preventing or treating disease [82]. Medicine in these areas has historically been treated through approaches that would now be considered “alternative”, such as through herbs or plant remedies, but which also hold value and have proven efficacy in some conditions [83]. The dismissal of alternative approaches by health professionals further fueled distrust and at times compelled individuals to reject effective approaches to treating disease. Research is crucial to bridge this gap and to demonstrate which natural products are effective and safe for particular conditions.

Working in an RHC setting requires the efficient use of basic clinic infrastructure to manage the study, including the waiting rooms, the clinical exam rooms, and the front desk staff. Employing clinical phlebotomists for the collection of biological samples expands the capacity of these types of studies and provides an opportunity for them to contribute to human research. However, phlebotomists are still required to perform their clinic duties, which can pose challenges to such studies, particularly on busy days. Another major limitation observed in the RHC settings is the paucity of research equipment for storing and collecting samples and data, requiring the transportation of samples across long distances. Finally, a major strength of RHCs is that their staff must be HIPPA compliant, so maintaining participant confidentiality is commonplace and de rigueur for this setting.

## 5. Conclusions and Limitations

Our pilot study tentatively suggests the potential positive effects of chronic blueberry treatment on the behavioral symptoms of MDD and GAD in a rural population. However, the possible peripheral mediators of these effects remain unclear. This study had several limitations, including the following: (1) the relatively small and homogenous sample; (2) the lack of longitudinal data on the participants’ diet (we only administered the diet questionnaire at baseline for recruitment purposes); (3) the lack of objective compliance measures such as analytical measures of blueberry metabolites in blood; (4) limitations associated with our biomarker measures (described above); and (5) the limited ability of the MDI, our self-report survey of depressive symptoms, which is not sensitive to changes in the mild to moderate categories of depression symptoms. Given the preliminary nature of these findings, future work in larger and more diverse samples and that implement objective compliance measures is required to draw strong conclusions and uncover the physiological mechanisms underlying the observed behavioral effects.

Despite some important tradeoffs associated with performing scientific research in Rural Health Clinics, such studies may yield significant results and positively impact the health of individuals in these highly overlooked areas. In short, these populations should be included in future health research. We suggest that performing research at RHCs increases the socioeconomic and cultural diversity of research populations, enabling more generalizable, or targeted studies, depending on context and design.

## Data Availability

The raw data supporting the conclusions of this article will be made available by the authors on request.

## Supplementary Materials

The following supporting information can be downloaded at: https://www.mdpi.com/article/10.3390/nu17233720/s1, Supplementary Files S1–S15: Study design and data.

## Abbreviations

The following abbreviations are used in this manuscript:

bl	Baseline Timepoint
CNS	Central Nervous System
CRP	*C*-Reactive Protein
CSF	Cerebral Spinal Fluid
FC	Fold Change
GAD	General Anxiety Disorder
GAD-7	Generalized Anxiety Disorder 7-Item Questionnaire
HDRS	Hamilton Depression Rating Scale
MDD	Major Depressive Disorder
MDI	Major Depression Inventory
mid	Middle Timepoint;
RHC	Rural Health Clinic
SIGH-D	Structured Interview Guide for the Hamilton Depression Rating Scale
