# Supplementary material for: Effects of Blueberry Supplementation on Depression and Anxiety Symptoms in a Rural Louisiana Population
Source: Nutrients. 2025 Nov 27;17(23):3720. doi: 10.3390/nu17233720 (PMC12694358; doi:10.3390/nu17233720)
Supplement: Supplementary file 1 [file nutrients-17-03720-s001.zip › SupplementaryFileS1.pdf]

ACTION ON PROTOCOL APPROVAL REQUEST

LSU

Dr. Dennis Landin, Chair
130 David Boyd Hall
Baton Rouge, LA 70803
P: 225.578.8692
F: 225.578.5983
irb@lsu.edu
lsu.edu/research

TO: Joseph Francis
Comparative Biomedical Sciences

FROM: Dennis Landin
Chair, Institutional Review Board

DATE: February 28, 2019

RE: IRB# 3762

TITLE: Survey study: The Potential Health Benefits of Blueberry on Progressive Neuro-Inflammatory Disorders: PTSD and Major Depression

New Protocol/Modification/Continuation: Modification

Brief Modification Description: Add a coversheet to the front of the survey packets.

Review type: Full ___ Expedited _X_ **Review date:** 2/28/2019

Risk Factor: Minimal _X_ Uncertain ___ Greater Than Minimal___

Approved _X_ **Disapproved** ___

Approval Date: 2/28/2019 **Approval Expiration Date:** N/A

Re-review frequency: (annual unless otherwise stated)

Number of subjects approved: 120

LSU Proposal Number (if applicable):

By: Dennis Landin, Chairman ___

PRINCIPAL INVESTIGATOR: PLEASE READ THE FOLLOWING –
Continuing approval is CONDITIONAL on:

1. Adherence to the approved protocol, familiarity with, and adherence to the ethical standards of the Belmont Report, and LSU's Assurance of Compliance with DHHS regulations for the protection of human subjects*
2. Prior approval of a change in protocol, including revision of the consent documents or an increase in the number of subjects over that approved.
3. Obtaining renewed approval (or submittal of a termination report), prior to the approval expiration date, upon request by the IRB office (irrespective of when the project actually begins); notification of project termination.
4. Retention of documentation of informed consent and study records for at least 3 years after the study ends.
5. Continuing attention to the physical and psychological well-being and informed consent of the individual participants including notification of new information that might affect consent.
6. A prompt report to the IRB of any adverse event affecting a participant potentially arising from the study.
7. Notification of the IRB of a serious compliance failure.
8. **SPECIAL NOTE: Make sure you use bcc when emailing more than one recipient.**

**All investigators and support staff have access to copies of the Belmont Report, LSU's Assurance with DHHS, DHHS (45 CFR 46) and FDA regulations governing use of human subjects, and other relevant documents in print in this office or on our World Wide Web site at http://www.lsu.edu/irb*
