# Supplementary material for: Effects of Blueberry Supplementation on Depression and Anxiety Symptoms in a Rural Louisiana Population
Source: Nutrients. 2025 Nov 27;17(23):3720. doi: 10.3390/nu17233720 (PMC12694358; doi:10.3390/nu17233720)
Supplement: Supplementary file 1 [file nutrients-17-03720-s001.zip › SupplementaryFileS10.pdf]

# MSD & CRP scripts

2024-01-02

## Load Packages

```
library(tidyverse)

## ── Attaching core tidyverse packages ──────────────────────── tidyv
erse 2.0.0 ──
## ✔ dplyr     1.1.1     ✔ readr     2.1.4
## ✔ forcats   1.0.0     ✔ stringr   1.5.0
## ✔ ggplot2   3.4.1     ✔ tibble    3.2.1
## ✔ lubridate 1.9.2     ✔ tidyr     1.3.0
## ✔ purrr     1.0.1
## ── Conflicts ────────────────────────────────────────── tidyverse_c
onflicts() ──
## ✖ dplyr::filter() masks stats::filter()
## ✖ dplyr::lag()    masks stats::lag()
## ℹ Use the conflicted package (<http://conflicted.r-lib.org/>) to fo
rce all conflicts to become errors

library(readxl)
library(lme4)

## Loading required package: Matrix
##
## Attaching package: 'Matrix'
##
## The following objects are masked from 'package:tidyr':
##
##     expand, pack, unpack

library(lmerTest)

##
## Attaching package: 'lmerTest'
##
## The following object is masked from 'package:lme4':
##
##     lmer
##
## The following object is masked from 'package:stats':
##
##     step
```

```r
library(lsmeans)

## Loading required package: emmeans
## The 'lsmeans' package is now basically a front end for 'emmeans'.
## Users are encouraged to switch the rest of the way.
## See help('transition') for more information, including how to
## convert old 'lsmeans' objects and scripts to work with 'emmeans'.

library(rstatix)

##
## Attaching package: 'rstatix'
##
## The following object is masked from 'package:stats':
##
##     filter

library(ggpubr)
```

### Upload Data

```r
bbd_polished <- read_excel("data/polished_bbd.xlsx", sheet = "polished
_bbd",
                           na = "NA")


bbd_polished_factors <- bbd_polished |>
  mutate(tx = as.factor(tx)) |>
  mutate(arm = as.factor(arm)) |>
  mutate(appt_cat = as.factor(appt_cat)) |>
  mutate(bb_first = as.factor(bb_first))


bbd_mid_filter <- bbd_polished_factors |>
  filter(appt_cat != "mid" & HDRS_num != "NA")


bbd_bl <- bbd_polished_factors |> filter(appt_cat == "bl")


bbd_mid <- bbd_polished_factors |> filter(appt_cat == "mid")



bbd_post <- bbd_polished_factors |> filter(appt_cat == "post")

bbd_HDRS_filter <- bbd_polished_factors |> filter(appt_cat != "mid")
```

## Identify MSD Outliers

```
mean(log10(bbd_polished$il1b_2), na.rm = TRUE) +
  (sd(log10(bbd_polished$il1b_2), na.rm = TRUE) *3)

## [1] 3.065931

mean(log10(bbd_polished$il6_2), na.rm = TRUE) +
  (sd(log10(bbd_polished$il6_2), na.rm = TRUE)*3)

## [1] 4.347291

mean(log10(bbd_polished$il10_2), na.rm = TRUE) +
  (sd(log10(bbd_polished$il10_2), na.rm = TRUE)*3)

## [1] 3.98512

mean(log10(bbd_polished$ifny_2), na.rm = TRUE) +
  (sd(log10(bbd_polished$ifny_2), na.rm = TRUE)*3)

## [1] 4.077921

mean(log10(bbd_polished$tnfa_2), na.rm = TRUE) +
  (sd(log10(bbd_polished$tnfa_2), na.rm = TRUE)*3)

## [1] 3.395276
```

## IFN-y Modeling and Evaluation

```
outlier_ifny <- bbd_polished_factors |>
  filter(log10(ifny_2) < 4.077921)
out_ifny_2_mem_int <- lmer(log10(ifny_2) ~ tx +
                             arm +
                             tx : appt_cat +
                             bb_first +
                             appt_cat +
                             (1 | de_id),
                           data = outlier_ifny)
print(out_ifny_2_mem_int)

## Linear mixed model fit by REML ['lmerModLmerTest']
## Formula: log10(ifny_2) ~ tx + arm + tx:appt_cat + bb_first + appt_c
at +
##     (1 | de_id)
##    Data: outlier_ifny
## REML criterion at convergence: 150.3028
## Random effects:
##  Groups   Name        Std.Dev.
##  de_id    (Intercept) 0.1480
##  Residual             0.3575
```

```
## Number of obs: 146, groups:  de_id, 27
## Fixed Effects:
##            (Intercept)               txplacebo                       a
rm2
##               2.827996                0.038915                   0.049
208
##              bb_first1             appt_catmid               appt_catp
ost
##              -0.064754                0.003724                  -0.108
295
##  txplacebo:appt_catmid  txplacebo:appt_catpost
##              -0.025965                0.023357
```

```
summary(out_ifny_2_mem_int)
```

```
## Linear mixed model fit by REML. t-tests use Satterthwaite's method
[
## lmerModLmerTest]
## Formula: log10(ifny_2) ~ tx + arm + tx:appt_cat + bb_first + appt_c
at +
##     (1 | de_id)
##    Data: outlier_ifny
##
## REML criterion at convergence: 150.3
##
## Scaled residuals:
##     Min      1Q  Median      3Q     Max
## -7.8812 -0.3471 -0.0689  0.3734  2.6524
##
## Random effects:
##  Groups   Name        Variance Std.Dev.
##  de_id    (Intercept) 0.02191  0.1480
##  Residual             0.12783  0.3575
## Number of obs: 146, groups:  de_id, 27
##
## Fixed effects:
##                          Estimate Std. Error         df t value Pr(
>|t|)
## (Intercept)              2.827996   0.093169 100.800426  30.353   <
2e-16 ***
## txplacebo                0.038915   0.101653 115.919208   0.383
0.703
## arm2                     0.049208   0.059973 119.390666   0.821
0.414
## bb_first1               -0.064754   0.082709  25.192679  -0.783
0.441
```

```
## appt_catmid              0.003724   0.102582 115.225604   0.036
0.971
## appt_catpost            -0.108295   0.101312 114.711275  -1.069
0.287
## txplacebo:appt_catmid   -0.025965   0.144880 114.816956  -0.179
0.858
## txplacebo:appt_catpost   0.023357   0.144982 115.273750   0.161
0.872
## ---
## Signif. codes:  0 '***' 0.001 '**' 0.01 '*' 0.05 '.' 0.1 ' ' 1
##
## Correlation of Fixed Effects:
##                (Intr) txplcb arm2   bb_fr1 appt_ctm appt_ctp txplcb
:ppt_ctm
## txplacebo      -0.557
## arm2           -0.282 -0.023
## bb_first1      -0.485  0.039  0.008
## appt_catmid    -0.539  0.494 -0.038  0.027
## appt_catpst    -0.544  0.498  0.000  0.000  0.494
## txplcb:ppt_ctm  0.375 -0.698  0.036 -0.011 -0.707   -0.350
## txplcb:ppt_ctp  0.379 -0.696  0.001  0.001 -0.345   -0.699    0.490
```

```
confint(out_ifny_2_mem_int)
```

```
## Computing profile confidence intervals ...
```

```
##                               2.5 %     97.5 %
## .sig01                   0.04543597 0.23081884
## .sigma                   0.30862468 0.39778689
## (Intercept)              2.64948034 3.00677129
## txplacebo               -0.15602297 0.23605295
## arm2                    -0.06627601 0.16456864
## bb_first1               -0.22685299 0.09596437
## appt_catmid             -0.19402378 0.20106950
## appt_catpost            -0.30302532 0.08727274
## txplacebo:appt_catmid   -0.30563731 0.25249816
## txplacebo:appt_catpost  -0.25755453 0.30149295
```

```
plot(out_ifny_2_mem_int)
```

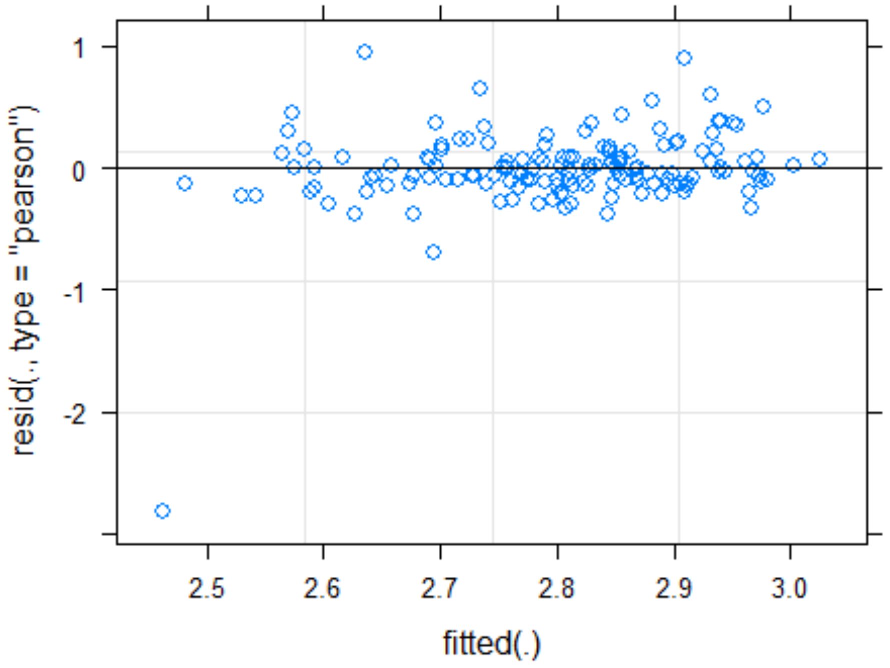


```
ls_means(out_ifny_2_mem_int)

## Least Squares Means table:
##
##                            Estimate Std. Error    df t value    lower
upper
## txblueberry                2.785366   0.050821  53.1  54.807 2.683434
2.887297
## txplacebo                  2.823411   0.051300  54.4  55.037 2.720580
2.926242
## arm1                       2.779784   0.049610  50.6  56.033 2.680168
2.879401
## arm2                       2.828992   0.052492  56.9  53.893 2.723872
2.934112
## bb_first0                  2.836765   0.059121  24.6  47.982 2.714891
2.958639
## bb_first1                  2.772011   0.057822  25.9  47.941 2.653125
2.890898
## appt_catbl                 2.839680   0.058279  80.9  48.726 2.723721
2.955639
## appt_catmid                2.830421   0.059287  83.3  47.741 2.712508
2.948335
## appt_catpost               2.743063   0.059301  83.9  46.257 2.625135
2.860992
## txblueberry:appt_catbl     2.820223   0.077415 127.4  36.430 2.667038
```

```
2.973408
## txplacebo:appt_catbl     2.859137   0.077243 127.4  37.015 2.706292
3.011983
## txblueberry:appt_catmid  2.823947   0.078734 128.5  35.867 2.668164
2.979729
## txplacebo:appt_catmid    2.836896   0.078815 128.5  35.995 2.680954
2.992838
## txblueberry:appt_catpost 2.711927   0.077415 127.4  35.031 2.558742
2.865112
## txplacebo:appt_catpost   2.774200   0.080344 129.7  34.529 2.615244
2.933155
##                           Pr(>|t|)
## txblueberry              < 2.2e-16 ***
## txplacebo                < 2.2e-16 ***
## arm1                     < 2.2e-16 ***
## arm2                     < 2.2e-16 ***
## bb_first0                < 2.2e-16 ***
## bb_first1                < 2.2e-16 ***
## appt_catbl               < 2.2e-16 ***
## appt_catmid              < 2.2e-16 ***
## appt_catpost             < 2.2e-16 ***
## txblueberry:appt_catbl   < 2.2e-16 ***
## txplacebo:appt_catbl     < 2.2e-16 ***
## txblueberry:appt_catmid  < 2.2e-16 ***
## txplacebo:appt_catmid    < 2.2e-16 ***
## txblueberry:appt_catpost < 2.2e-16 ***
## txplacebo:appt_catpost   < 2.2e-16 ***
## ---
## Signif. codes:  0 '***' 0.001 '**' 0.01 '*' 0.05 '.' 0.1 ' ' 1
##
##   Confidence level: 95%
##   Degrees of freedom method: Satterthwaite
```

```
ls_means(out_ifny_2_mem_int, pairwise = TRUE)
```

```
## Least Squares Means table:
##
##                                                         Estimate Std.
Error    df
## txblueberry - txplacebo                               -0.0380456  0.05
99377 119.4
## arm1 - arm2                                           -0.0492080  0.05
99729 119.4
## bb_first0 - bb_first1                                  0.0647542  0.08
27095  25.2
## appt_catbl - appt_catmid                               0.0092586  0.07
```

```
25739 115.8
## appt_catbl - appt_catpost                             0.0966166  0.07
24910 115.3
## appt_catmid - appt_catpost                            0.0873580  0.07
33486 115.8
## txblueberry:appt_catbl - txplacebo:appt_catbl        -0.0389148  0.10
16531 115.9
## txblueberry:appt_catbl - txblueberry:appt_catmid     -0.0037240  0.10
25821 115.2
## txblueberry:appt_catbl - txplacebo:appt_catmid       -0.0166736  0.10
28594 116.5
## txblueberry:appt_catbl - txblueberry:appt_catpost     0.1082953  0.10
13120 114.7
## txblueberry:appt_catbl - txplacebo:appt_catpost       0.0460231  0.10
40684 117.1
## txplacebo:appt_catbl - txblueberry:appt_catmid        0.0351908  0.10
27312 116.7
## txplacebo:appt_catbl - txplacebo:appt_catmid          0.0222411  0.10
24988 115.4
## txplacebo:appt_catbl - txblueberry:appt_catpost       0.1472101  0.10
16531 115.9
## txplacebo:appt_catbl - txplacebo:appt_catpost         0.0849379  0.10
37094 115.8
## txblueberry:appt_catmid - txplacebo:appt_catmid      -0.0129497  0.10
37379 116.4
## txblueberry:appt_catmid - txblueberry:appt_catpost  0.1120193  0.10
25821 115.2
## txblueberry:appt_catmid - txplacebo:appt_catpost     0.0497471  0.10
51448 118.0
## txplacebo:appt_catmid - txblueberry:appt_catpost     0.1249690  0.10
28594 116.5
## txplacebo:appt_catmid - txplacebo:appt_catpost       0.0626968  0.10
46579 115.4
## txblueberry:appt_catpost - txplacebo:appt_catpost  -0.0622722  0.10
40684 117.1
##                                                    t value      low
er
## txblueberry - txplacebo                            -0.6348 -0.15672
45
## arm1 - arm2                                        -0.8205 -0.16795
63
## bb_first0 - bb_first1                               0.7829 -0.10552
31
## appt_catbl - appt_catmid                            0.1276 -0.13448
59
## appt_catbl - appt_catpost                           1.3328 -0.04697
```

```
04
## appt_catmid - appt_catpost                            1.1910 -0.05792
01
## txblueberry:appt_catbl - txplacebo:appt_catbl      -0.3828 -0.24025
30
## txblueberry:appt_catbl - txblueberry:appt_catmid   -0.0363 -0.20691
51
## txblueberry:appt_catbl - txplacebo:appt_catmid     -0.1621 -0.22039
03
## txblueberry:appt_catbl - txblueberry:appt_catpost   1.0689 -0.09238
97
## txblueberry:appt_catbl - txplacebo:appt_catpost     0.4422 -0.16007
75
## txplacebo:appt_catbl - txblueberry:appt_catmid      0.3426 -0.16826
76
## txplacebo:appt_catbl - txplacebo:appt_catmid        0.2170 -0.18078
19
## txplacebo:appt_catbl - txblueberry:appt_catpost     1.4482 -0.05412
82
## txplacebo:appt_catbl - txplacebo:appt_catpost       0.8190 -0.12047
52
## txblueberry:appt_catmid - txplacebo:appt_catmid    -0.1248 -0.21840
90
## txblueberry:appt_catmid - txblueberry:appt_catpost  1.0920 -0.09117
18
## txblueberry:appt_catmid - txplacebo:appt_catpost    0.4731 -0.15846
88
## txplacebo:appt_catmid - txblueberry:appt_catpost    1.2149 -0.07874
77
## txplacebo:appt_catmid - txplacebo:appt_catpost      0.5991 -0.14460
35
## txblueberry:appt_catpost - txplacebo:appt_catpost  -0.5984 -0.26837
29
##                                                         upper Pr(>|
t|)
## txblueberry - txplacebo                             0.0806334   0.5
268
## arm1 - arm2                                         0.0695402   0.4
136
## bb_first0 - bb_first1                               0.2350315   0.4
410
## appt_catbl - appt_catmid                            0.1530031   0.8
987
## appt_catbl - appt_catpost                           0.2402036   0.1
852
## appt_catmid - appt_catpost                          0.2326361   0.2
```

```
361
## txblueberry:appt_catbl - txplacebo:appt_catbl       0.1624235   0.7
026
## txblueberry:appt_catbl - txblueberry:appt_catmid    0.1994671   0.9
711
## txblueberry:appt_catbl - txplacebo:appt_catmid      0.1870430   0.8
715
## txblueberry:appt_catbl - txblueberry:appt_catpost   0.3089803   0.2
873
## txblueberry:appt_catbl - txplacebo:appt_catpost     0.2521238   0.6
591
## txplacebo:appt_catbl - txblueberry:appt_catmid      0.2386491   0.7
326
## txplacebo:appt_catbl - txplacebo:appt_catmid        0.2252641   0.8
286
## txplacebo:appt_catbl - txblueberry:appt_catpost     0.3485484   0.1
503
## txplacebo:appt_catbl - txplacebo:appt_catpost       0.2903510   0.4
145
## txblueberry:appt_catmid - txplacebo:appt_catmid     0.1925097   0.9
009
## txblueberry:appt_catmid - txblueberry:appt_catpost  0.3152104   0.2
771
## txblueberry:appt_catmid - txplacebo:appt_catpost    0.2579630   0.6
370
## txplacebo:appt_catmid - txblueberry:appt_catpost    0.3286856   0.2
268
## txplacebo:appt_catmid - txplacebo:appt_catpost      0.2699970   0.5
503
## txblueberry:appt_catpost - txplacebo:appt_catpost   0.1438284   0.5
507
##
##   Confidence level: 95%
##   Degrees of freedom method: Satterthwaite
```

### IL-10 Modeling and Evaluation

```
outlier_il10 <- bbd_polished_factors |>
  filter(log10(il10_2) < 3.98512)

out_il10_2_mem_int <- lmer(log10(il10_2) ~ tx +
                             arm +
                             tx : appt_cat +
                             bb_first +
                             appt_cat +
                             (1 | de_id),
```

```r
                          data = outlier_il10)
print(out_il10_2_mem_int)
```

```
## Linear mixed model fit by REML ['lmerModLmerTest']
## Formula: log10(il10_2) ~ tx + arm + tx:appt_cat + bb_first + appt_c
at +
##      (1 | de_id)
##    Data: outlier_il10
## REML criterion at convergence: -5.2104
## Random effects:
##  Groups   Name        Std.Dev.
##  de_id    (Intercept) 0.1822
##  Residual             0.1820
## Number of obs: 140, groups:  de_id, 26
## Fixed Effects:
##             (Intercept)                txplacebo                    a
rm2
##                2.724684                 0.005806                -0.009
199
##               bb_first1              appt_catmid              appt_catp
ost
##                0.060479                -0.033648                 0.034
038
##  txplacebo:appt_catmid  txplacebo:appt_catpost
##                0.022788                -0.056314
```

```r
plot(out_il10_2_mem_int)
```

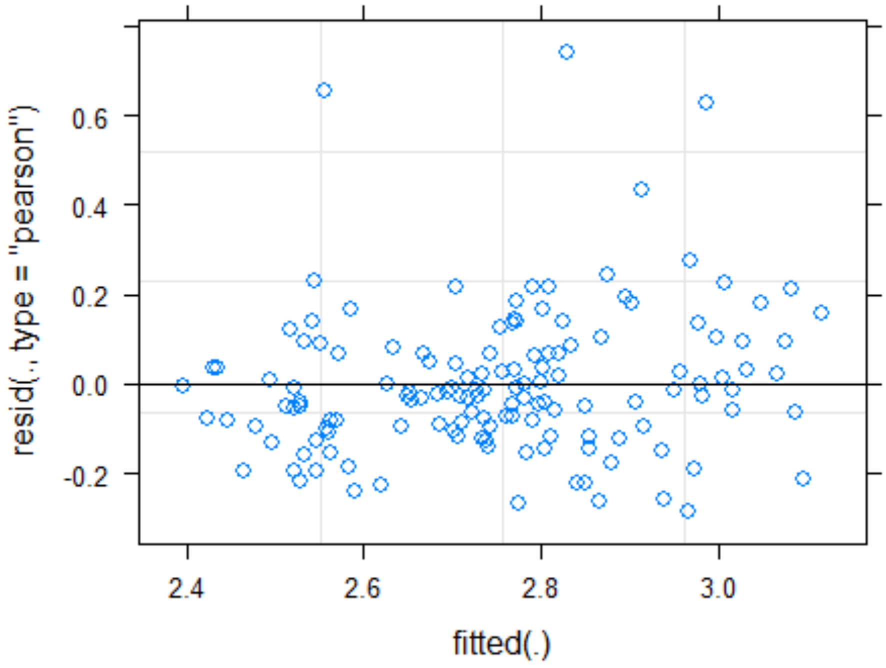


```
summary(out_il10_2_mem_int)

## Linear mixed model fit by REML. t-tests use Satterthwaite's method
[
## lmerModLmerTest]
## Formula: log10(il10_2) ~ tx + arm + tx:appt_cat + bb_first + appt_c
at +
##     (1 | de_id)
##    Data: outlier_il10
##
## REML criterion at convergence: -5.2
##
## Scaled residuals:
##     Min      1Q  Median      3Q     Max
## -1.5643 -0.5246 -0.1227  0.3962  4.0655
##
## Random effects:
##  Groups   Name        Variance Std.Dev.
##  de_id    (Intercept) 0.03319  0.1822
##  Residual             0.03311  0.1820
## Number of obs: 140, groups:  de_id, 26
##
## Fixed effects:
##                            Estimate Std. Error         df t value Pr(
>|t|)
```

```
## (Intercept)               2.724684   0.067903  46.300273  40.126   <
2e-16 ***
## txplacebo                 0.005806   0.052427 109.645627   0.111
0.912
## arm2                     -0.009199   0.031499 111.226449  -0.292
0.771
## bb_first1                 0.060479   0.078262  24.734646   0.773
0.447
## appt_catmid              -0.033648   0.052795 109.187266  -0.637
0.525
## appt_catpost              0.034038   0.052775 109.197082   0.645
0.520
## txplacebo:appt_catmid     0.022788   0.074988 109.159210   0.304
0.762
## txplacebo:appt_catpost  -0.056314   0.075804 109.661933  -0.743
0.459
## ---
## Signif. codes:  0 '***' 0.001 '**' 0.01 '*' 0.05 '.' 0.1 ' ' 1
##
## Correlation of Fixed Effects:
##                 (Intr) txplcb arm2   bb_fr1 appt_ctm appt_ctp txplcb
:ppt_ctm
## txplacebo      -0.382
## arm2           -0.200 -0.037
## bb_first1      -0.628  0.021  0.003
## appt_catmid    -0.370  0.479 -0.026  0.010
## appt_catpst    -0.370  0.482  0.004 -0.001  0.474
## txplcb:ppt_ctm  0.255 -0.691  0.031 -0.002 -0.701   -0.337
## txplcb:ppt_ctp  0.256 -0.686  0.000  0.001 -0.331   -0.700    0.482
```

```r
confint(out_il10_2_mem_int)
```

```
## Computing profile confidence intervals ...
```

```
##                              2.5 %      97.5 %
## .sig01                  0.12760063 0.24457623
## .sigma                  0.15646422 0.20274011
## (Intercept)             2.59382231 2.85554296
## txplacebo              -0.09504789 0.10665636
## arm2                   -0.06981396 0.05134904
## bb_first1              -0.09234094 0.21320714
## appt_catmid            -0.13518142 0.06795398
## appt_catpost           -0.06753153 0.13552735
## txplacebo:appt_catmid  -0.12142728 0.16709636
## txplacebo:appt_catpost -0.20196936 0.08969321
```

```r
ls_means(out_il10_2_mem_int)
```

```
## Least Squares Means table:
##
##                             Estimate Std. Error   df t value    lower
upper
## txblueberry                 2.750454   0.042133 32.9  65.281 2.664719
2.836188
## txplacebo                   2.745084   0.042238 33.2  64.991 2.659168
2.831001
## arm1                        2.752369   0.041677 31.7  66.041 2.667445
2.837293
## arm2                        2.743169   0.042684 34.3  64.268 2.656456
2.829883
## bb_first0                   2.717530   0.057254 24.4  47.465 2.599478
2.835582
## bb_first1                   2.778008   0.053360 25.1  52.062 2.668130
2.887887
## appt_catbl                  2.753226   0.044396 40.1  62.015 2.663503
2.842950
## appt_catmid                 2.730973   0.044992 41.9  60.700 2.640171
2.821775
## appt_catpost                2.759108   0.045159 42.5  61.097 2.668006
2.850210
## txblueberry:appt_catbl      2.750323   0.051293 64.6  53.620 2.647872
2.852775
## txplacebo:appt_catbl        2.756129   0.051820 66.5  53.187 2.652682
2.859577
## txblueberry:appt_catmid     2.716676   0.052531 69.0  51.716 2.611879
2.821473
## txplacebo:appt_catmid       2.745270   0.052510 68.9  52.281 2.640513
2.850027
## txblueberry:appt_catpost    2.784362   0.052620 69.3  52.914 2.679394
2.889330
## txplacebo:appt_catpost      2.733854   0.053222 71.4  51.367 2.627744
2.839964
##                              Pr(>|t|)
## txblueberry                 < 2.2e-16 ***
## txplacebo                   < 2.2e-16 ***
## arm1                        < 2.2e-16 ***
## arm2                        < 2.2e-16 ***
## bb_first0                   < 2.2e-16 ***
## bb_first1                   < 2.2e-16 ***
## appt_catbl                  < 2.2e-16 ***
## appt_catmid                 < 2.2e-16 ***
## appt_catpost                < 2.2e-16 ***
## txblueberry:appt_catbl      < 2.2e-16 ***
## txplacebo:appt_catbl        < 2.2e-16 ***
```

```
## txblueberry:appt_catmid  < 2.2e-16 ***
## txplacebo:appt_catmid    < 2.2e-16 ***
## txblueberry:appt_catpost < 2.2e-16 ***
## txplacebo:appt_catpost   < 2.2e-16 ***
## ---
## Signif. codes:  0 '***' 0.001 '**' 0.01 '*' 0.05 '.' 0.1 ' ' 1
##
##   Confidence level: 95%
##   Degrees of freedom method: Satterthwaite
```

```
ls_means(out_il10_2_mem_int, pairwise = TRUE)
```

```
## Least Squares Means table:
##
##                                                         Estimate Std.
Error    df
## txblueberry - txplacebo                                0.0053693  0.03
15115 111.2
## arm1 - arm2                                            0.0091995  0.03
14994 111.2
## bb_first0 - bb_first1                                 -0.0604787  0.07
82624  24.7
## appt_catbl - appt_catmid                               0.0222534  0.03
76335 109.5
## appt_catbl - appt_catpost                             -0.0058815  0.03
77080 109.1
## appt_catmid - appt_catpost                            -0.0281349  0.03
85144 109.7
## txblueberry:appt_catbl - txplacebo:appt_catbl         -0.0058058  0.05
24271 109.6
## txblueberry:appt_catbl - txblueberry:appt_catmid       0.0336475  0.05
27954 109.2
## txblueberry:appt_catbl - txplacebo:appt_catmid         0.0050534  0.05
31079 109.8
## txblueberry:appt_catbl - txblueberry:appt_catpost     -0.0340385  0.05
27754 109.2
## txblueberry:appt_catbl - txplacebo:appt_catpost        0.0164695  0.05
38350 110.0
## txplacebo:appt_catbl - txblueberry:appt_catmid         0.0394534  0.05
37189 110.1
## txplacebo:appt_catbl - txplacebo:appt_catmid          0.0108593  0.05
34489 109.5
## txplacebo:appt_catbl - txblueberry:appt_catpost       -0.0282326  0.05
35406 109.6
## txplacebo:appt_catbl - txplacebo:appt_catpost          0.0222754  0.05
41446 109.6
```

```
## txblueberry:appt_catmid - txplacebo:appt_catmid    -0.0285941  0.05
41902 109.9
## txblueberry:appt_catmid - txblueberry:appt_catpost -0.0676860  0.05
41197 109.6
## txblueberry:appt_catmid - txplacebo:appt_catpost   -0.0171780  0.05
51400 110.5
## txplacebo:appt_catmid - txblueberry:appt_catpost   -0.0390919  0.05
44555 110.3
## txplacebo:appt_catmid - txplacebo:appt_catpost      0.0114161  0.05
45962 109.4
## txblueberry:appt_catpost - txplacebo:appt_catpost   0.0505080  0.05
51860 110.4
##                                                    t value      low
er
## txblueberry - txplacebo                             0.1704 -0.05707
12
## arm1 - arm2                                         0.2921 -0.05321
72
## bb_first0 - bb_first1                              -0.7728 -0.22175
07
## appt_catbl - appt_catmid                            0.5913 -0.05233
09
## appt_catbl - appt_catpost                          -0.1560 -0.08061
66
## appt_catmid - appt_catpost                         -0.7305 -0.10446
39
## txblueberry:appt_catbl - txplacebo:appt_catbl      -0.1107 -0.10970
78
## txblueberry:appt_catbl - txblueberry:appt_catmid    0.6373 -0.07098
91
## txblueberry:appt_catbl - txplacebo:appt_catmid      0.0952 -0.10019
59
## txblueberry:appt_catbl - txblueberry:appt_catpost  -0.6450 -0.13863
55
## txblueberry:appt_catbl - txplacebo:appt_catpost     0.3059 -0.09021
89
## txplacebo:appt_catbl - txblueberry:appt_catmid      0.7344 -0.06700
37
## txplacebo:appt_catbl - txplacebo:appt_catmid        0.2032 -0.09506
93
## txplacebo:appt_catbl - txblueberry:appt_catpost    -0.5273 -0.13434
15
## txplacebo:appt_catbl - txplacebo:appt_catpost       0.4114 -0.08503
09
## txblueberry:appt_catmid - txplacebo:appt_catmid    -0.5277 -0.13598
74
```

```
## txblueberry:appt_catmid - txblueberry:appt_catpost -1.2507 -0.17494
31
## txblueberry:appt_catmid - txplacebo:appt_catpost   -0.3115 -0.12644
70
## txplacebo:appt_catmid - txblueberry:appt_catpost   -0.7179 -0.14700
72
## txplacebo:appt_catmid - txplacebo:appt_catpost      0.2091 -0.09678
76
## txblueberry:appt_catpost - txplacebo:appt_catpost   0.9152 -0.05885
28
##                                                          upper Pr(>|
t|)
## txblueberry - txplacebo                             0.0678099   0.8
650
## arm1 - arm2                                         0.0716162   0.7
708
## bb_first0 - bb_first1                               0.1007934   0.4
470
## appt_catbl - appt_catmid                            0.0968377   0.5
555
## appt_catbl - appt_catpost                           0.0688535   0.8
763
## appt_catmid - appt_catpost                          0.0481940   0.4
666
## txblueberry:appt_catbl - txplacebo:appt_catbl       0.0980962   0.9
120
## txblueberry:appt_catbl - txblueberry:appt_catmid    0.1382842   0.5
253
## txblueberry:appt_catbl - txplacebo:appt_catmid      0.1103028   0.9
244
## txblueberry:appt_catbl - txblueberry:appt_catpost   0.0705586   0.5
203
## txblueberry:appt_catbl - txplacebo:appt_catpost     0.1231579   0.7
602
## txplacebo:appt_catbl - txblueberry:appt_catmid      0.1459104   0.4
642
## txplacebo:appt_catbl - txplacebo:appt_catmid        0.1167878   0.8
394
## txplacebo:appt_catbl - txblueberry:appt_catpost     0.0778763   0.5
990
## txplacebo:appt_catbl - txplacebo:appt_catpost       0.1295816   0.6
816
## txblueberry:appt_catmid - txplacebo:appt_catmid     0.0787991   0.5
988
## txblueberry:appt_catmid - txblueberry:appt_catpost  0.0395711   0.2
137
```

```
## txblueberry:appt_catmid - txplacebo:appt_catpost    0.0920910   0.7
560
## txplacebo:appt_catmid - txblueberry:appt_catpost    0.0688234   0.4
744
## txplacebo:appt_catmid - txplacebo:appt_catpost      0.1196198   0.8
348
## txblueberry:appt_catpost - txplacebo:appt_catpost   0.1598688   0.3
621
##
##   Confidence level: 95%
##   Degrees of freedom method: Satterthwaite
```

### IL-6 Modeling and Evaluation

```
outlier_il6 <- bbd_polished_factors |>
  filter(log10(il6_2) < 4.347291)

out_il6_2_mem_int <- lmer(log10(il6_2) ~ tx +
                           arm +
                           tx : appt_cat +
                           bb_first +
                           appt_cat +
                           (1 | de_id),
                         data = outlier_il6)
print(out_il6_2_mem_int)
```

```
## Linear mixed model fit by REML ['lmerModLmerTest']
## Formula: log10(il6_2) ~ tx + arm + tx:appt_cat + bb_first + appt_ca
t +
##     (1 | de_id)
##    Data: outlier_il6
## REML criterion at convergence: -6.8012
## Random effects:
##  Groups   Name        Std.Dev.
##  de_id    (Intercept) 0.2399
##  Residual             0.1724
## Number of obs: 146, groups:  de_id, 27
## Fixed Effects:
##            (Intercept)               txplacebo                    a
rm2
##               3.538706               -0.064362               -0.005
915
##              bb_first1             appt_catmid             appt_catp
ost
##              -0.016306               -0.038450               -0.087
186
```

```
##  txplacebo:appt_catmid  txplacebo:appt_catpost
##               0.047137               0.062918
```

```
summary(out_il6_2_mem_int)
```

```
## Linear mixed model fit by REML. t-tests use Satterthwaite's method
[
## lmerModLmerTest]
## Formula: log10(il6_2) ~ tx + arm + tx:appt_cat + bb_first + appt_ca
t +
##     (1 | de_id)
##    Data: outlier_il6
##
## REML criterion at convergence: -6.8
##
## Scaled residuals:
##     Min      1Q  Median      3Q     Max
## -2.9510 -0.4595 -0.0844  0.3747  3.6589
##
## Random effects:
##  Groups   Name        Variance Std.Dev.
##  de_id    (Intercept) 0.05756  0.2399
##  Residual             0.02972  0.1724
## Number of obs: 146, groups:  de_id, 27
##
## Fixed effects:
##                            Estimate Std. Error         df t value Pr(
>|t|)
## (Intercept)                3.538706   0.077583  37.793659  45.612   <
2e-16 ***
## txplacebo                 -0.064362   0.048684 113.608637  -1.322   0
.1888
## arm2                      -0.005915   0.029249 114.613414  -0.202   0
.8401
## bb_first1                 -0.016306   0.096907  25.070844  -0.168   0
.8677
## appt_catmid               -0.038450   0.049018 113.315204  -0.784   0
.4344
## appt_catpost              -0.087186   0.049000 113.321042  -1.779   0
.0779 .
## txplacebo:appt_catmid      0.047137   0.069609 113.300587   0.677   0
.4997
## txplacebo:appt_catpost     0.062918   0.070367 113.598701   0.894   0
.3731
## ---
## Signif. codes:  0 '***' 0.001 '**' 0.01 '*' 0.05 '.' 0.1 ' ' 1
```

```
##
## Correlation of Fixed Effects:
##                 (Intr) txplcb arm2   bb_fr1 appt_ctm appt_ctp txplcb
:ppt_ctm
## txplacebo      -0.315
## arm2           -0.171 -0.011
## bb_first1      -0.652  0.016  0.005
## appt_catmid    -0.301  0.479 -0.027  0.007
## appt_catpst    -0.301  0.483  0.003  0.000  0.475
## txplcb:ppt_ctm  0.207 -0.691  0.031 -0.002 -0.701   -0.338
## txplcb:ppt_ctp  0.208 -0.686  0.002  0.001 -0.331   -0.700    0.483
```

```
confint(out_il6_2_mem_int)
```

```
## Computing profile confidence intervals ...
```

```
##                               2.5 %      97.5 %
## .sig01                   0.17440976 0.315657687
## .sigma                   0.14874150 0.191841808
## (Intercept)              3.38854876 3.688976865
## txplacebo               -0.15803068 0.029454399
## arm2                    -0.06216176 0.050467418
## bb_first1               -0.20579675 0.172950496
## appt_catmid             -0.13275582 0.056023934
## appt_catpost            -0.18134989 0.007401846
## txplacebo:appt_catmid   -0.08699497 0.181082166
## txplacebo:appt_catpost -0.07305343 0.198060696
```

```
plot(out_il6_2_mem_int)
```

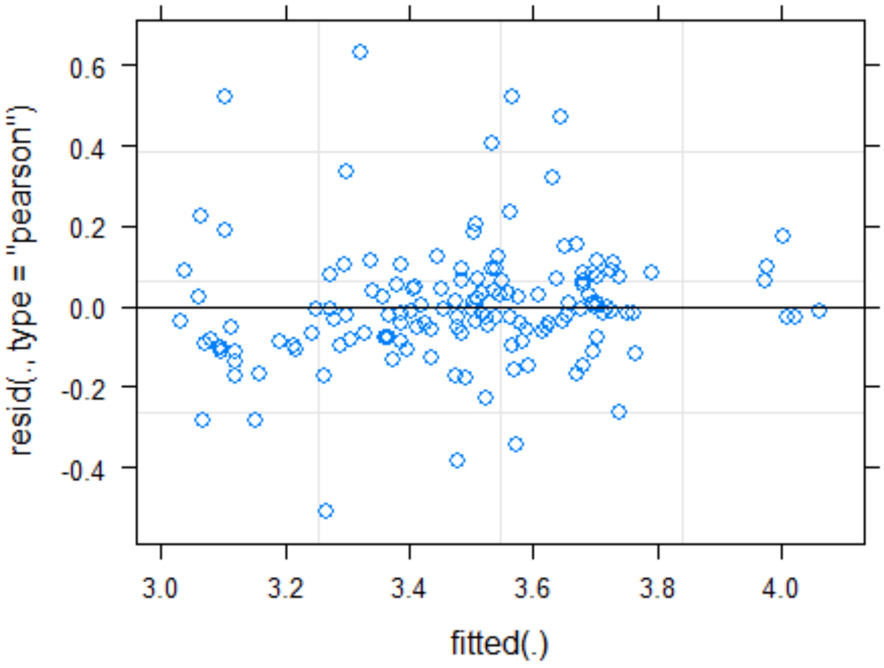


```
ls_means(out_il6_2_mem_int)

## Least Squares Means table:
##
##                          Estimate Std. Error   df t value    lower
upper
## txblueberry              3.485717   0.050548 29.6  68.958 3.382426
3.589008
## txplacebo                3.458041   0.050681 29.9  68.231 3.354522
3.561559
## arm1                     3.474836   0.050245 29.0  69.158 3.372069
3.577603
## arm2                     3.468922   0.050979 30.5  68.046 3.364886
3.572957
## bb_first0                3.480032   0.069642 24.9  49.970 3.336565
3.623499
## bb_first1                3.463726   0.067388 25.3  51.400 3.325017
3.602436
## appt_catbl               3.495415   0.052221 33.6  66.934 3.389244
3.601586
## appt_catmid              3.480534   0.052660 34.7  66.094 3.373592
3.587475
## appt_catpost             3.439688   0.052764 35.0  65.190 3.332566
3.546810
## txblueberry:appt_catbl   3.527596   0.057392 47.6  61.465 3.412175
```

```
3.643017
## txplacebo:appt_catbl     3.463234   0.057840 48.8   59.876 3.346992
3.579476
## txblueberry:appt_catmid  3.489146   0.058349 50.3   59.798 3.371967
3.606324
## txplacebo:appt_catmid    3.471922   0.058377 50.4   59.474 3.354691
3.589152
## txblueberry:appt_catpost 3.440410   0.058386 50.4   58.925 3.323162
3.557658
## txplacebo:appt_catpost   3.438966   0.058897 51.9   58.389 3.320776
3.557157
##                           Pr(>|t|)
## txblueberry              < 2.2e-16 ***
## txplacebo                < 2.2e-16 ***
## arm1                     < 2.2e-16 ***
## arm2                     < 2.2e-16 ***
## bb_first0                < 2.2e-16 ***
## bb_first1                < 2.2e-16 ***
## appt_catbl               < 2.2e-16 ***
## appt_catmid              < 2.2e-16 ***
## appt_catpost             < 2.2e-16 ***
## txblueberry:appt_catbl   < 2.2e-16 ***
## txplacebo:appt_catbl     < 2.2e-16 ***
## txblueberry:appt_catmid  < 2.2e-16 ***
## txplacebo:appt_catmid    < 2.2e-16 ***
## txblueberry:appt_catpost < 2.2e-16 ***
## txplacebo:appt_catpost   < 2.2e-16 ***
## ---
## Signif. codes:  0 '***' 0.001 '**' 0.01 '*' 0.05 '.' 0.1 ' ' 1
##
##   Confidence level: 95%
##   Degrees of freedom method: Satterthwaite
```

```
ls_means(out_il6_2_mem_int, pairwise = TRUE)
```

```
## Least Squares Means table:
##
##                                                        Estimate Std.
Error    df
## txblueberry - txplacebo                               0.0276764  0.02
92600 114.6
## arm1 - arm2                                           0.0059148  0.02
92490 114.6
## bb_first0 - bb_first1                                 0.0163057  0.09
69072  25.1
## appt_catbl - appt_catmid                              0.0148814  0.03
```

```
49417 113.5
## appt_catbl - appt_catpost                         0.0557267  0.03
49940 113.3
## appt_catmid - appt_catpost                        0.0408453  0.03
57311 113.6
## txblueberry:appt_catbl - txplacebo:appt_catbl      0.0643616  0.04
86844 113.6
## txblueberry:appt_catbl - txblueberry:appt_catmid   0.0384501  0.04
90182 113.3
## txblueberry:appt_catbl - txplacebo:appt_catmid     0.0556742  0.04
93203 113.7
## txblueberry:appt_catbl - txblueberry:appt_catpost  0.0871856  0.04
89999 113.3
## txblueberry:appt_catbl - txplacebo:appt_catpost    0.0886293  0.04
99580 113.8
## txplacebo:appt_catbl - txblueberry:appt_catmid    -0.0259115  0.04
98912 113.9
## txplacebo:appt_catbl - txplacebo:appt_catmid      -0.0086874  0.04
96165 113.5
## txplacebo:appt_catbl - txblueberry:appt_catpost    0.0228240  0.04
96694 113.6
## txplacebo:appt_catbl - txplacebo:appt_catpost      0.0242677  0.05
02388 113.6
## txblueberry:appt_catmid - txplacebo:appt_catmid    0.0172241  0.05
03223 113.8
## txblueberry:appt_catmid - txblueberry:appt_catpost 0.0487355  0.05
02166 113.6
## txblueberry:appt_catmid - txplacebo:appt_catpost   0.0501792  0.05
11804 114.1
## txplacebo:appt_catmid - txblueberry:appt_catpost   0.0315114  0.05
05380 114.0
## txplacebo:appt_catmid - txplacebo:appt_catpost     0.0329551  0.05
06288 113.4
## txblueberry:appt_catpost - txplacebo:appt_catpost  0.0014437  0.05
11792 114.1
##                                                   t value      low
er
## txblueberry - txplacebo                            0.9459 -0.03028
40
## arm1 - arm2                                        0.2022 -0.05202
39
## bb_first0 - bb_first1                              0.1683 -0.18324
97
## appt_catbl - appt_catmid                           0.4259 -0.05434
11
## appt_catbl - appt_catpost                          1.5925 -0.01360
```

```
08
## appt_catmid - appt_catpost                              1.1431 -0.02994
04
## txblueberry:appt_catbl - txplacebo:appt_catbl         1.3220 -0.03208
54
## txblueberry:appt_catbl - txblueberry:appt_catmid      0.7844 -0.05866
09
## txblueberry:appt_catbl - txplacebo:appt_catmid        1.1288 -0.04203
15
## txblueberry:appt_catbl - txblueberry:appt_catpost     1.7793 -0.00988
91
## txblueberry:appt_catbl - txplacebo:appt_catpost       1.7741 -0.01033
89
## txplacebo:appt_catbl - txblueberry:appt_catmid       -0.5194 -0.12474
65
## txplacebo:appt_catbl - txplacebo:appt_catmid         -0.1751 -0.10698
20
## txplacebo:appt_catbl - txblueberry:appt_catpost       0.4595 -0.07557
45
## txplacebo:appt_catbl - txplacebo:appt_catpost         0.4830 -0.07525
92
## txblueberry:appt_catmid - txplacebo:appt_catmid       0.3423 -0.08246
61
## txblueberry:appt_catmid - txblueberry:appt_catpost    0.9705 -0.05074
74
## txblueberry:appt_catmid - txplacebo:appt_catpost      0.9804 -0.05120
76
## txplacebo:appt_catmid - txblueberry:appt_catpost      0.6235 -0.06860
41
## txplacebo:appt_catmid - txplacebo:appt_catpost        0.6509 -0.06734
55
## txblueberry:appt_catpost - txplacebo:appt_catpost     0.0282 -0.09994
11
##                                                             upper Pr(>|
t|)
## txblueberry - txplacebo                                0.0856368  0.34
620
## arm1 - arm2                                            0.0638535  0.84
010
## bb_first0 - bb_first1                                  0.2158612  0.86
773
## appt_catbl - appt_catmid                               0.0841038  0.67
099
## appt_catbl - appt_catpost                              0.1250541  0.11
407
## appt_catmid - appt_catpost                             0.1116309  0.25
```

```
539
## txblueberry:appt_catbl - txplacebo:appt_catbl       0.1608086  0.18
882
## txblueberry:appt_catbl - txblueberry:appt_catmid    0.1355612  0.43
444
## txblueberry:appt_catbl - txplacebo:appt_catmid      0.1533799  0.26
135
## txblueberry:appt_catbl - txblueberry:appt_catpost   0.1842603  0.07
787 .
## txblueberry:appt_catbl - txplacebo:appt_catpost     0.1875974  0.07
872 .
## txplacebo:appt_catbl - txblueberry:appt_catmid      0.0729236  0.60
452
## txplacebo:appt_catbl - txplacebo:appt_catmid        0.0896072  0.86
132
## txplacebo:appt_catbl - txblueberry:appt_catpost     0.1212225  0.64
674
## txplacebo:appt_catbl - txplacebo:appt_catpost       0.1237946  0.62
999
## txblueberry:appt_catmid - txplacebo:appt_catmid     0.1169143  0.73
278
## txblueberry:appt_catmid - txblueberry:appt_catpost  0.1482184  0.33
386
## txblueberry:appt_catmid - txplacebo:appt_catpost    0.1515659  0.32
894
## txplacebo:appt_catmid - txblueberry:appt_catpost    0.1316270  0.53
419
## txplacebo:appt_catmid - txplacebo:appt_catpost      0.1332557  0.51
642
## txblueberry:appt_catpost - txplacebo:appt_catpost   0.1028284  0.97
755
## ---
## Signif. codes:  0 '***' 0.001 '**' 0.01 '*' 0.05 '.' 0.1 ' ' 1
##
##   Confidence level: 95%
##   Degrees of freedom method: Satterthwaite
```

### IL-1b Modeling and Evaluation

```
outlier_il1b <- bbd_polished_factors |>
  filter(log10(il1b_2) < 3.065931)
out_il1b_2_mem_int <- lmer(log10(il1b_2) ~ tx +
                           arm +
                           tx : appt_cat +
                           bb_first +
                           appt_cat +
                           (1 | de_id),
```

```
                              data = outlier_il1b)
print(out_il1b_2_mem_int)

## Linear mixed model fit by REML ['lmerModLmerTest']
## Formula: log10(il1b_2) ~ tx + arm + tx:appt_cat + bb_first + appt_c
at +
##     (1 | de_id)
##    Data: outlier_il1b
## REML criterion at convergence: 12.8008
## Random effects:
##  Groups   Name        Std.Dev.
##  de_id    (Intercept) 0.2079
##  Residual             0.1896
## Number of obs: 126, groups:  de_id, 27
## Fixed Effects:
##             (Intercept)                txplacebo                    a
rm2
##               2.1467873                0.0619685                0.0027
183
##               bb_first1              appt_catmid              appt_catp
ost
##              -0.0007877               -0.0121900               -0.1307
426
##  txplacebo:appt_catmid  txplacebo:appt_catpost
##              -0.0536438               -0.0006524

summary(out_il1b_2_mem_int)

## Linear mixed model fit by REML. t-tests use Satterthwaite's method
[
## lmerModLmerTest]
## Formula: log10(il1b_2) ~ tx + arm + tx:appt_cat + bb_first + appt_c
at +
##     (1 | de_id)
##    Data: outlier_il1b
##
## REML criterion at convergence: 12.8
##
## Scaled residuals:
##     Min      1Q  Median      3Q     Max
## -1.9693 -0.5957 -0.1211  0.3844  3.0100
##
## Random effects:
##  Groups   Name        Variance Std.Dev.
##  de_id    (Intercept) 0.04323  0.2079
##  Residual             0.03595  0.1896
```

```
## Number of obs: 126, groups:  de_id, 27
##
## Fixed effects:
##                              Estimate Std. Error         df t value Pr(
>|t|)
## (Intercept)                 2.1467873  0.0748070 39.9163270  28.698   <
2e-16 ***
## txplacebo                   0.0619685  0.0569604 94.1869305   1.088   0
.2794
## arm2                        0.0027183  0.0348809 93.3995104   0.078   0
.9380
## bb_first1                  -0.0007877  0.0884121 21.2818110  -0.009   0
.9930
## appt_catmid                -0.0121900  0.0586374 92.3176968  -0.208   0
.8358
## appt_catpost               -0.1307426  0.0595336 92.4305909  -2.196   0
.0306 *
## txplacebo:appt_catmid      -0.0536438  0.0829669 92.9807870  -0.647   0
.5195
## txplacebo:appt_catpost     -0.0006524  0.0844709 92.2307348  -0.008   0
.9939
## ---
## Signif. codes:  0 '***' 0.001 '**' 0.01 '*' 0.05 '.' 0.1 ' ' 1
##
## Correlation of Fixed Effects:
##                  (Intr) txplcb arm2   bb_fr1 appt_ctm appt_ctp txplcb
:ppt_ctm
## txplacebo        -0.384
## arm2             -0.178 -0.056
## bb_first1        -0.635  0.039 -0.005
## appt_catmid      -0.343  0.473 -0.069  0.015
## appt_catpst      -0.337  0.470 -0.051  0.009  0.459
## txplcb:ppt_ctm    0.267 -0.688  0.046 -0.033 -0.709   -0.334
## txplcb:ppt_ctp    0.244 -0.667  0.043 -0.011 -0.320   -0.706    0.465
```

```r
confint(out_il1b_2_mem_int)
```

```
## Computing profile confidence intervals ...
```

```
##                                  2.5 %      97.5 %
## .sig01                      0.13926413  0.28549455
## .sigma                      0.16061089  0.21354792
## (Intercept)                 2.00375334  2.29343444
## txplacebo                  -0.04683391  0.17268558
## arm2                       -0.06435695  0.06957604
## bb_first1                  -0.17661377  0.17040080
```

```
## appt_catmid              -0.12507562  0.10015285
## appt_catpost             -0.24521493 -0.01659202
## txplacebo:appt_catmid    -0.21338218  0.10527292
## txplacebo:appt_catpost   -0.16451203  0.16075602

plot(out_il1b_2_mem_int)
```

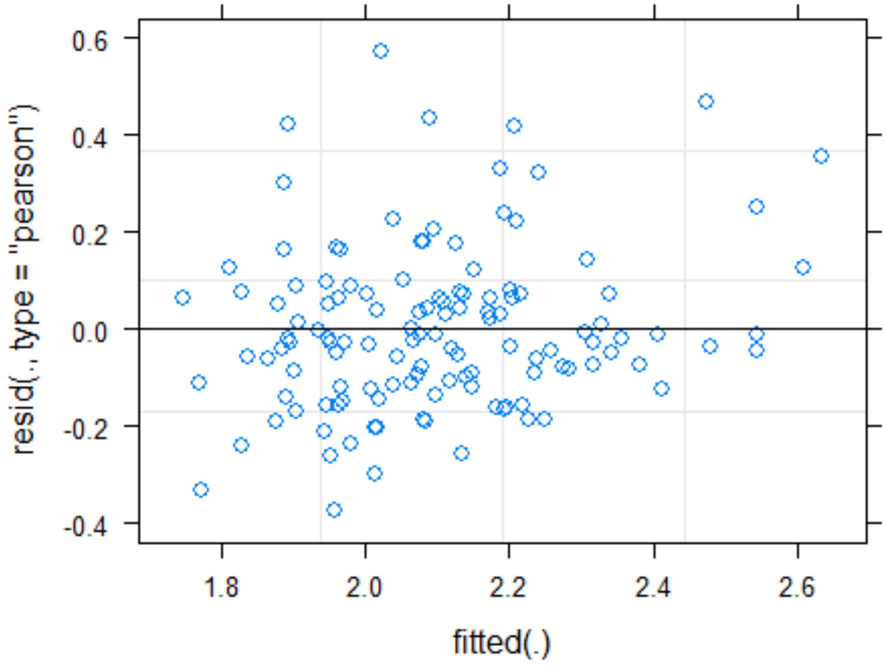


```
ls_means(out_il1b_2_mem_int)

## Least Squares Means table:
##
##                            Estimate Std. Error   df t value    lower
upper
## txblueberry                2.100108   0.047669 28.7  44.056 2.002564
2.197653
## txplacebo                  2.143978   0.047484 28.4  45.152 2.046767
2.241189
## arm1                       2.120684   0.047139 27.6  44.988 2.024054
2.217314
## arm2                       2.123402   0.047992 29.4  44.245 2.025305
2.221500
## bb_first0                  2.122437   0.064216 21.6  33.051 1.989122
2.255753
## bb_first1                  2.121649   0.060841 21.0  34.872 1.995118
2.248181
```

```
## appt_catbl                2.178737   0.049020 32.8   44.446 2.078985
2.278489
## appt_catmid               2.139725   0.050940 36.8   42.005 2.036488
2.242962
## appt_catpost              2.047668   0.051686 38.8   39.617 1.943103
2.152233
## txblueberry:appt_catbl    2.147753   0.056431 53.8   38.060 2.034607
2.260899
## txplacebo:appt_catbl      2.209721   0.056953 55.4   38.799 2.095602
2.323840
## txblueberry:appt_catmid   2.135563   0.059610 62.5   35.825 2.016422
2.254703
## txplacebo:appt_catmid     2.143887   0.058711 60.0   36.516 2.026448
2.261326
## txblueberry:appt_catpost 2.017010   0.060557 65.4   33.308 1.896082
2.137938
## txplacebo:appt_catpost   2.078326   0.060487 65.3   34.360 1.957534
2.199118
##                           Pr(>|t|)
## txblueberry              < 2.2e-16 ***
## txplacebo                < 2.2e-16 ***
## arm1                     < 2.2e-16 ***
## arm2                     < 2.2e-16 ***
## bb_first0                < 2.2e-16 ***
## bb_first1                < 2.2e-16 ***
## appt_catbl               < 2.2e-16 ***
## appt_catmid              < 2.2e-16 ***
## appt_catpost             < 2.2e-16 ***
## txblueberry:appt_catbl   < 2.2e-16 ***
## txplacebo:appt_catbl     < 2.2e-16 ***
## txblueberry:appt_catmid  < 2.2e-16 ***
## txplacebo:appt_catmid    < 2.2e-16 ***
## txblueberry:appt_catpost < 2.2e-16 ***
## txplacebo:appt_catpost   < 2.2e-16 ***
## ---
## Signif. codes:  0 '***' 0.001 '**' 0.01 '*' 0.05 '.' 0.1 ' ' 1
##
##   Confidence level: 95%
##   Degrees of freedom method: Satterthwaite
```

```
ls_means(out_il1b_2_mem_int, pairwise = TRUE)
```

```
## Least Squares Means table:
##
##                                                       Estimate  Std
. Error   df
```

```
## txblueberry - txplacebo                                   -0.04386973  0.0
3493067 93.1
## arm1 - arm2                                               -0.00271834  0.0
3488087 93.4
## bb_first0 - bb_first1                                      0.00078774  0.0
8841208 21.3
## appt_catbl - appt_catmid                                   0.03901186  0.0
4134923 92.1
## appt_catbl - appt_catpost                                  0.13106876  0.0
4218677 92.2
## appt_catmid - appt_catpost                                 0.09205689  0.0
4349559 91.4
## txblueberry:appt_catbl - txplacebo:appt_catbl             -0.06196845  0.0
5696035 94.2
## txblueberry:appt_catbl - txblueberry:appt_catmid           0.01218996  0.0
5863744 92.3
## txblueberry:appt_catbl - txplacebo:appt_catmid             0.00386531  0.0
5762447 91.7
## txblueberry:appt_catbl - txblueberry:appt_catpost          0.13074257  0.0
5953363 92.4
## txblueberry:appt_catbl - txplacebo:appt_catpost            0.06942649  0.0
5999577 93.3
## txplacebo:appt_catbl - txblueberry:appt_catmid             0.07415842  0.0
5935403 92.7
## txplacebo:appt_catbl - txplacebo:appt_catmid               0.06583377  0.0
5850571 92.7
## txplacebo:appt_catbl - txblueberry:appt_catpost            0.19271102  0.0
6002232 92.4
## txplacebo:appt_catbl - txplacebo:appt_catpost              0.13139494  0.0
5985697 92.0
## txblueberry:appt_catmid - txplacebo:appt_catmid           -0.00832465  0.0
6017665 91.4
## txblueberry:appt_catmid - txblueberry:appt_catpost         0.11855261  0.0
6149203 90.6
## txblueberry:appt_catmid - txplacebo:appt_catpost           0.05723653  0.0
6202096 91.7
## txplacebo:appt_catmid - txblueberry:appt_catpost           0.12687726  0.0
6145679 92.0
## txplacebo:appt_catmid - txplacebo:appt_catpost             0.06556118  0.0
6123554 91.8
## txblueberry:appt_catpost - txplacebo:appt_catpost         -0.06131608  0.0
6297543 91.8
##                                                           t value       lo
wer
## txblueberry - txplacebo                                   -1.2559 -0.11323
429
```

```
## arm1 - arm2                                                 -0.0779 -0.07198
094
## bb_first0 - bb_first1                                        0.0089 -0.18292
712
## appt_catbl - appt_catmid                                     0.9435 -0.04311
047
## appt_catbl - appt_catpost                                    3.1069  0.04728
405
## appt_catmid - appt_catpost                                   2.1165  0.00566
323
## txblueberry:appt_catbl - txplacebo:appt_catbl               -1.0879 -0.17506
164
## txblueberry:appt_catbl - txblueberry:appt_catmid             0.2079 -0.10426
371
## txblueberry:appt_catbl - txplacebo:appt_catmid               0.0671 -0.11058
646
## txblueberry:appt_catbl - txblueberry:appt_catpost            2.1961  0.01251
099
## txblueberry:appt_catbl - txplacebo:appt_catpost              1.1572 -0.04970
803
## txplacebo:appt_catbl - txblueberry:appt_catmid               1.2494 -0.04371
136
## txplacebo:appt_catbl - txplacebo:appt_catmid                 1.1253 -0.05035
134
## txplacebo:appt_catbl - txblueberry:appt_catpost              3.2107  0.07350
845
## txplacebo:appt_catbl - txplacebo:appt_catpost                2.1951  0.01251
307
## txblueberry:appt_catmid - txplacebo:appt_catmid             -0.1383 -0.12785
177
## txblueberry:appt_catmid - txblueberry:appt_catpost           1.9279 -0.00360
181
## txblueberry:appt_catmid - txplacebo:appt_catpost             0.9229 -0.06594
830
## txplacebo:appt_catmid - txblueberry:appt_catpost             2.0645  0.00481
842
## txplacebo:appt_catmid - txplacebo:appt_catpost               1.0706 -0.05606
169
## txblueberry:appt_catpost - txplacebo:appt_catpost           -0.9737 -0.18639
381
##                                                                   upper Pr(>
|t|)
## txblueberry - txplacebo                                      0.02549483 0.21
2292
## arm1 - arm2                                                  0.06654426 0.93
8049
```

```
## bb_first0 - bb_first1                                 0.18450260 0.99
2974
## appt_catbl - appt_catmid                              0.12113420 0.34
7909
## appt_catbl - appt_catpost                             0.21485346 0.00
2514 **
## appt_catmid - appt_catpost                            0.17845055 0.03
7023 *
## txblueberry:appt_catbl - txplacebo:appt_catbl         0.05112473 0.27
9405
## txblueberry:appt_catbl - txblueberry:appt_catmid      0.12864364 0.83
5775
## txblueberry:appt_catbl - txplacebo:appt_catmid        0.11831708 0.94
6666
## txblueberry:appt_catbl - txblueberry:appt_catpost     0.24897415 0.03
0586 *
## txblueberry:appt_catbl - txplacebo:appt_catpost       0.18856101 0.25
0149
## txplacebo:appt_catbl - txblueberry:appt_catmid        0.19202819 0.21
4654
## txplacebo:appt_catbl - txplacebo:appt_catmid          0.18201887 0.26
3385
## txplacebo:appt_catbl - txblueberry:appt_catpost       0.31191360 0.00
1822 **
## txplacebo:appt_catbl - txplacebo:appt_catpost         0.25027681 0.03
0671 *
## txblueberry:appt_catmid - txplacebo:appt_catmid       0.11120247 0.89
0279
## txblueberry:appt_catmid - txblueberry:appt_catpost    0.24070702 0.05
6997 .
## txblueberry:appt_catmid - txplacebo:appt_catpost      0.18042136 0.35
8504
## txplacebo:appt_catmid - txblueberry:appt_catpost      0.24893609 0.04
1786 *
## txplacebo:appt_catmid - txplacebo:appt_catpost        0.18718405 0.28
7139
## txblueberry:appt_catpost - txplacebo:appt_catpost     0.06376165 0.33
2788
## ---
## Signif. codes:  0 '***' 0.001 '**' 0.01 '*' 0.05 '.' 0.1 ' ' 1
##
##   Confidence level: 95%
##   Degrees of freedom method: Satterthwaite
```

### TNF-a Modeling and Evaluation

```
outlier_tnfa <- bbd_polished_factors |>
  filter(log10(tnfa_2) < 3.395276)

out_tnfa_2_mem_int <- lmer(log10(tnfa_2) ~ tx +
                             arm +
                             tx : appt_cat +
                             bb_first +
                             appt_cat +
                             (1 | de_id),
                           data = outlier_tnfa)
print(out_tnfa_2_mem_int)
```

```
## Linear mixed model fit by REML ['lmerModLmerTest']
## Formula: log10(tnfa_2) ~ tx + arm + tx:appt_cat + bb_first + appt_c
at +
##     (1 | de_id)
##    Data: outlier_tnfa
## REML criterion at convergence: -252.1181
## Random effects:
##  Groups   Name        Std.Dev.
##  de_id    (Intercept) 0.11084
##  Residual             0.06925
## Number of obs: 146, groups:  de_id, 27
## Fixed Effects:
##             (Intercept)                txplacebo                    a
rm2
##               3.0123719                0.0020886                -0.0047
836
##               bb_first1              appt_catmid               appt_catp
ost
##               0.0115310               -0.0237541                -0.0189
074
##  txplacebo:appt_catmid  txplacebo:appt_catpost
##              -0.0004806               -0.0164679
```

```
summary(out_tnfa_2_mem_int)
```

```
## Linear mixed model fit by REML. t-tests use Satterthwaite's method
[
## lmerModLmerTest]
## Formula: log10(tnfa_2) ~ tx + arm + tx:appt_cat + bb_first + appt_c
at +
##     (1 | de_id)
##    Data: outlier_tnfa
##
```

```
## REML criterion at convergence: -252.1
##
## Scaled residuals:
##     Min      1Q  Median      3Q     Max
## -2.3640 -0.4974 -0.0412  0.4238  3.9175
##
## Random effects:
##  Groups   Name        Variance Std.Dev.
##  de_id    (Intercept) 0.012285 0.11084
##  Residual             0.004795 0.06925
## Number of obs: 146, groups:  de_id, 27
##
## Fixed effects:
##                            Estimate Std. Error         df t value Pr(
>|t|)
## (Intercept)               3.012e+00  3.467e-02  3.502e+01  86.887   <
2e-16 ***
## txplacebo                 2.089e-03  1.956e-02  1.137e+02   0.107
0.915
## arm2                     -4.784e-03  1.176e-02  1.145e+02  -0.407
0.685
## bb_first1                 1.153e-02  4.428e-02  2.526e+01   0.260
0.797
## appt_catmid              -2.375e-02  1.969e-02  1.134e+02  -1.206
0.230
## appt_catpost             -1.891e-02  1.968e-02  1.134e+02  -0.961
0.339
## txplacebo:appt_catmid  -4.806e-04  2.796e-02  1.134e+02  -0.017
0.986
## txplacebo:appt_catpost -1.647e-02  2.827e-02  1.137e+02  -0.583
0.561
## ---
## Signif. codes:  0 '***' 0.001 '**' 0.01 '*' 0.05 '.' 0.1 ' ' 1
##
## Correlation of Fixed Effects:
##                  (Intr) txplcb arm2   bb_fr1 appt_ctm appt_ctp txplcb
:ppt_ctm
## txplacebo      -0.283
## arm2           -0.154 -0.011
## bb_first1      -0.666  0.014  0.004
## appt_catmid    -0.270  0.478 -0.027  0.006
## appt_catpst    -0.271  0.483  0.003  0.000  0.475
## txplcb:ppt_ctm  0.186 -0.691  0.032 -0.001 -0.701   -0.338
## txplcb:ppt_ctp  0.187 -0.686  0.002  0.001 -0.331   -0.700    0.483
```

```
confint(out_tnfa_2_mem_int)
```

```
## Computing profile confidence intervals ...

##                                2.5 %     97.5 %
## .sig01                    0.08144420 0.14500774
## .sigma                    0.05974643 0.07704192
## (Intercept)               2.94520369 3.07966918
## txplacebo                -0.03554283 0.03977831
## arm2                     -0.02737096 0.01791293
## bb_first1                -0.07506917 0.09796313
## appt_catmid              -0.06165577 0.01417065
## appt_catpost             -0.05679129 0.01900727
## txplacebo:appt_catmid    -0.05430401 0.05337437
## txplacebo:appt_catpost   -0.07089896 0.03796437
```

```
plot(out_tnfa_2_mem_int)
```

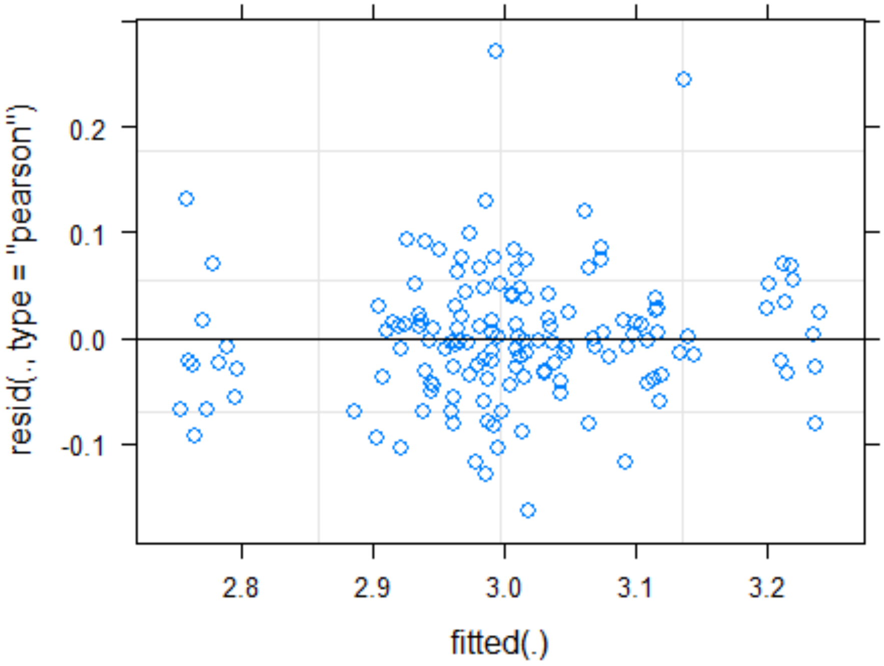


```
ls_means(out_tnfa_2_mem_int)

## Least Squares Means table:
##
##                             Estimate Std. Error   df t value    lower
upper
## txblueberry                 3.001525   0.022882 28.8 131.174 2.954710
3.048340
## txplacebo                   2.997964   0.022929 29.0 130.747 2.951069
```

```
3.044860
## arm1                     3.002136   0.022772 28.3 131.834 2.955510
3.048763
## arm2                     2.997353   0.023038 29.5 130.107 2.950271
3.044434
## bb_first0                2.993979   0.031832 25.1  94.054 2.928433
3.059525
## bb_first1                3.005510   0.030774 25.4  97.665 2.942185
3.068836
## appt_catbl               3.016790   0.023479 31.8 128.488 2.968955
3.064625
## appt_catmid              2.992796   0.023638 32.7 126.611 2.944685
3.040906
## appt_catpost             2.989649   0.023675 32.9 126.280 2.941474
3.037823
## txblueberry:appt_catbl   3.015746   0.025352 42.4 118.954 2.964599
3.066893
## txplacebo:appt_catbl     3.017834   0.025517 43.4 118.270 2.966390
3.069278
## txblueberry:appt_catmid  2.991992   0.025704 44.6 116.403 2.940208
3.043775
## txplacebo:appt_catmid    2.993600   0.025714 44.6 116.419 2.941797
3.045402
## txblueberry:appt_catpost 2.996838   0.025717 44.6 116.529 2.945029
3.048647
## txplacebo:appt_catpost   2.982459   0.025905 45.8 115.129 2.930308
3.034610
##                           Pr(>|t|)
## txblueberry              < 2.2e-16 ***
## txplacebo                < 2.2e-16 ***
## arm1                     < 2.2e-16 ***
## arm2                     < 2.2e-16 ***
## bb_first0                < 2.2e-16 ***
## bb_first1                < 2.2e-16 ***
## appt_catbl               < 2.2e-16 ***
## appt_catmid              < 2.2e-16 ***
## appt_catpost             < 2.2e-16 ***
## txblueberry:appt_catbl   < 2.2e-16 ***
## txplacebo:appt_catbl     < 2.2e-16 ***
## txblueberry:appt_catmid  < 2.2e-16 ***
## txplacebo:appt_catmid    < 2.2e-16 ***
## txblueberry:appt_catpost < 2.2e-16 ***
## txplacebo:appt_catpost   < 2.2e-16 ***
## ---
## Signif. codes:  0 '***' 0.001 '**' 0.01 '*' 0.05 '.' 0.1 ' ' 1
##
```

```
##   Confidence level: 95%
##   Degrees of freedom method: Satterthwaite
```

```
ls_means(out_tnfa_2_mem_int, pairwise = TRUE)
```

```
## Least Squares Means table:
##
##                                                            Estimate  Std
. Error
## txblueberry - txplacebo                                  0.00356086  0.0
1176168
## arm1 - arm2                                              0.00478359  0.0
1175719
## bb_first0 - bb_first1                                   -0.01153104  0.0
4427517
## appt_catbl - appt_catmid                                 0.02399435  0.0
1403758
## appt_catbl - appt_catpost                                0.02714140  0.0
1405691
## appt_catmid - appt_catpost                               0.00314706  0.0
1435538
## txblueberry:appt_catbl - txplacebo:appt_catbl           -0.00208864  0.0
1955951
## txblueberry:appt_catbl - txblueberry:appt_catmid         0.02375405  0.0
1969066
## txblueberry:appt_catbl - txplacebo:appt_catmid           0.02214600  0.0
1981605
## txblueberry:appt_catbl - txblueberry:appt_catpost        0.01890745  0.0
1968336
## txblueberry:appt_catbl - txplacebo:appt_catpost          0.03328672  0.0
2007337
## txplacebo:appt_catbl - txblueberry:appt_catmid           0.02584269  0.0
2004727
## txplacebo:appt_catbl - txplacebo:appt_catmid             0.02423464  0.0
1993292
## txplacebo:appt_catbl - txblueberry:appt_catpost          0.02099609  0.0
1995514
## txplacebo:appt_catbl - txplacebo:appt_catpost            0.03537536  0.0
2018351
## txblueberry:appt_catmid - txplacebo:appt_catmid         -0.00160805  0.0
2021937
## txblueberry:appt_catmid - txblueberry:appt_catpost -0.00484661  0.0
2017448
## txblueberry:appt_catmid - txplacebo:appt_catpost         0.00953267  0.0
2056780
## txplacebo:appt_catmid - txblueberry:appt_catpost        -0.00323855  0.0
```

```
2030805
## txplacebo:appt_catmid - txplacebo:appt_catpost      0.01114072  0.0
2033877
## txblueberry:appt_catpost - txplacebo:appt_catpost   0.01437927  0.0
2056685
##                                                        df t value
lower
## txblueberry - txplacebo                             114.5  0.3028 -0
.01973788
## arm1 - arm2                                         114.5  0.4069 -0
.01850629
## bb_first0 - bb_first1                                25.3 -0.2604 -0
.10266977
## appt_catbl - appt_catmid                            113.6  1.7093 -0
.00381504
## appt_catbl - appt_catpost                           113.4  1.9308 -0
.00070674
## appt_catmid - appt_catpost                          113.7  0.2192 -0
.02529172
## txblueberry:appt_catbl - txplacebo:appt_catbl       113.7 -0.1068 -0
.04083706
## txblueberry:appt_catbl - txblueberry:appt_catmid    113.4  1.2064 -0
.01525505
## txblueberry:appt_catbl - txplacebo:appt_catmid      113.8  1.1176 -0
.01711033
## txblueberry:appt_catbl - txblueberry:appt_catpost  113.4  0.9606 -0
.02008717
## txblueberry:appt_catbl - txplacebo:appt_catpost     113.8  1.6583 -0
.00647907
## txplacebo:appt_catbl - txblueberry:appt_catmid      113.9  1.2891 -0
.01387118
## txplacebo:appt_catbl - txplacebo:appt_catmid        113.6  1.2158 -0
.01525385
## txplacebo:appt_catbl - txblueberry:appt_catpost     113.7  1.0522 -0
.01853613
## txplacebo:appt_catbl - txplacebo:appt_catpost       113.6  1.7527 -0
.00460940
## txblueberry:appt_catmid - txplacebo:appt_catmid     113.8 -0.0795 -0
.04166317
## txblueberry:appt_catmid - txblueberry:appt_catpost 113.6 -0.2402 -0
.04481350
## txblueberry:appt_catmid - txplacebo:appt_catpost    114.1  0.4635 -0
.03121166
## txplacebo:appt_catmid - txblueberry:appt_catpost    114.0 -0.1595 -0
.04346877
## txplacebo:appt_catmid - txplacebo:appt_catpost      113.5  0.5478 -0
```

```
.02915201
## txblueberry:appt_catpost - txplacebo:appt_catpost  114.1  0.6991 -0
.02636331
##                                                              upper Pr(>
|t|)
## txblueberry - txplacebo                                 0.02685961  0.7
6263
## arm1 - arm2                                             0.02807347  0.6
8487
## bb_first0 - bb_first1                                   0.07960768  0.7
9664
## appt_catbl - appt_catmid                                0.05180374  0.0
9013 .
## appt_catbl - appt_catpost                               0.05498955  0.0
5600 .
## appt_catmid - appt_catpost                              0.03158583  0.8
2687
## txblueberry:appt_catbl - txplacebo:appt_catbl           0.03665979  0.9
1515
## txblueberry:appt_catbl - txblueberry:appt_catmid        0.06276316  0.2
3019
## txblueberry:appt_catbl - txplacebo:appt_catmid          0.06140234  0.2
6610
## txblueberry:appt_catbl - txblueberry:appt_catpost       0.05790207  0.3
3881
## txblueberry:appt_catbl - txplacebo:appt_catpost         0.07305252  0.1
0002
## txplacebo:appt_catbl - txblueberry:appt_catmid          0.06555656  0.1
9998
## txplacebo:appt_catbl - txplacebo:appt_catmid            0.06372312  0.2
2658
## txplacebo:appt_catbl - txblueberry:appt_catpost         0.06052830  0.2
9496
## txplacebo:appt_catbl - txplacebo:appt_catpost           0.07536012  0.0
8235 .
## txblueberry:appt_catmid - txplacebo:appt_catmid         0.03844707  0.9
3675
## txblueberry:appt_catmid - txblueberry:appt_catpost      0.03512029  0.8
1058
## txblueberry:appt_catmid - txplacebo:appt_catpost        0.05027700  0.6
4391
## txplacebo:appt_catmid - txblueberry:appt_catpost        0.03699166  0.8
7358
## txplacebo:appt_catmid - txplacebo:appt_catpost          0.05143345  0.5
8493
## txblueberry:appt_catpost - txplacebo:appt_catpost       0.05512186  0.4
```

```
8588
## ---
## Signif. codes:  0 '***' 0.001 '**' 0.01 '*' 0.05 '.' 0.1 ' ' 1
##
##   Confidence level: 95%
##   Degrees of freedom method: Satterthwaite
```

## CRP Modeling and Evaluation

```
crp_mem_int <- lmer(crp ~ tx +
                           arm +
                           tx : appt_cat +
                           bb_first +
                           appt_cat +
                           (1 | de_id),
                         data = bbd_polished_factors)
print(crp_mem_int)
```

```
## Linear mixed model fit by REML ['lmerModLmerTest']
## Formula: crp ~ tx + arm + tx:appt_cat + bb_first + appt_cat + (1 |
de_id)
##    Data: bbd_polished_factors
## REML criterion at convergence: 840.7048
## Random effects:
##  Groups   Name        Std.Dev.
##  de_id    (Intercept) 3.421
##  Residual             3.279
## Number of obs: 154, groups:  de_id, 31
## Fixed Effects:
##             (Intercept)                txplacebo                     a
rm2
##                  6.5476                  -0.9337                  -0.6
822
##               bb_first1              appt_catmid              appt_catp
ost
##                 -1.0215                  -0.2646                   0.1
531
##  txplacebo:appt_catmid  txplacebo:appt_catpost
##                  0.9265                   0.1699
```

```
plot(crp_mem_int)
```

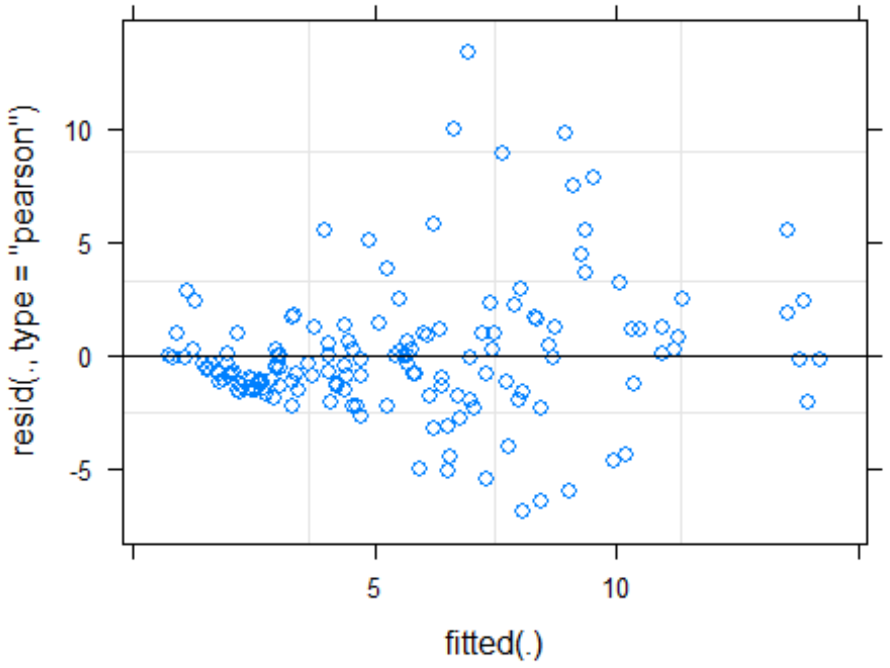


```
summary(crp_mem_int)

## Linear mixed model fit by REML. t-tests use Satterthwaite's method
[
## lmerModLmerTest]
## Formula: crp ~ tx + arm + tx:appt_cat + bb_first + appt_cat + (1 |
de_id)
##    Data: bbd_polished_factors
##
## REML criterion at convergence: 840.7
##
## Scaled residuals:
##     Min      1Q  Median      3Q     Max
## -2.0982 -0.4123 -0.1511  0.2953  4.0831
##
## Random effects:
##  Groups   Name        Variance Std.Dev.
##  de_id    (Intercept) 11.71    3.421
##  Residual             10.75    3.279
## Number of obs: 154, groups:  de_id, 31
##
## Fixed effects:
##                          Estimate Std. Error       df t value Pr(>|t|
)
## (Intercept)                6.5476     1.1159  62.8679   5.867 1.79e-0
```

```
7 ***
## txplacebo                -0.9337     0.9218 120.2850  -1.013    0.31
3
## arm2                     -0.6822     0.5470 121.0946  -1.247    0.21
5
## bb_first1                -1.0215     1.3520  29.6818  -0.756    0.45
6
## appt_catmid              -0.2646     0.9267 119.9925  -0.285    0.77
6
## appt_catpost              0.1531     0.9263 119.4749   0.165    0.86
9
## txplacebo:appt_catmid     0.9265     1.3357 120.2067   0.694    0.48
9
## txplacebo:appt_catpost    0.1699     1.3050 120.5382   0.130    0.89
7
## ---
## Signif. codes:  0 '***' 0.001 '**' 0.01 '*' 0.05 '.' 0.1 ' ' 1
##
## Correlation of Fixed Effects:
##                  (Intr) txplcb arm2   bb_fr1 appt_ctm appt_ctp txplcb
:ppt_ctm
## txplacebo        -0.424
## arm2             -0.265  0.077
## bb_first1        -0.546  0.004  0.001
## appt_catmid      -0.392  0.489  0.021 -0.017
## appt_catpst      -0.392  0.490  0.076 -0.038  0.489
## txplcb:ppt_ctm    0.285 -0.688 -0.041  0.003 -0.698   -0.345
## txplcb:ppt_ctp    0.281 -0.707 -0.059  0.030 -0.358   -0.721    0.500
```

```
ls_means(crp_mem_int)
```

```
## Least Squares Means table:
##
##                              Estimate Std. Error   df t value   lower
upper
## txblueberry                   5.65864    0.72769 39.4  7.7762 4.18720 7
.13007
## txplacebo                     5.09043    0.73010 39.8  6.9722 3.61463 6
.56623
## arm1                          5.71565    0.72060 38.1  7.9318 4.25703 7
.17428
## arm2                          5.03342    0.73737 41.1  6.8261 3.54442 6
.52242
## bb_first0                     5.88528    0.91289 30.2  6.4469 4.02135 7
.74920
## bb_first1                     4.86379    0.99700 29.2  4.8784 2.82545 6
```

```
.90214
## appt_catbl               5.22894    0.76859 48.4   6.8033 3.68390 6
.77399
## appt_catmid              5.42766    0.78616 52.0   6.9040 3.85013 7
.00519
## appt_catpost             5.46700    0.76929 48.5   7.1066 3.92065 7
.01335
## txblueberry:appt_catbl   5.69578    0.89004 77.5   6.3995 3.92367 7
.46788
## txplacebo:appt_catbl     4.76211    0.90230 80.2   5.2777 2.96654 6
.55768
## txblueberry:appt_catmid  5.43122    0.91101 82.2   5.9617 3.61899 7
.24346
## txplacebo:appt_catmid    5.42410    0.93564 87.7   5.7972 3.56462 7
.28357
## txblueberry:appt_catpost 5.84890    0.91162 82.4   6.4160 4.03555 7
.66226
## txplacebo:appt_catpost   5.08510    0.88209 75.6   5.7648 3.32810 6
.84209
##                           Pr(>|t|)
## txblueberry              1.770e-09 ***
## txplacebo                2.099e-08 ***
## arm1                     1.370e-09 ***
## arm2                     2.853e-08 ***
## bb_first0                3.923e-07 ***
## bb_first1                3.485e-05 ***
## appt_catbl               1.420e-08 ***
## appt_catmid              7.034e-09 ***
## appt_catpost             4.783e-09 ***
## txblueberry:appt_catbl   1.088e-08 ***
## txplacebo:appt_catbl     1.089e-06 ***
## txblueberry:appt_catmid  5.998e-08 ***
## txplacebo:appt_catmid    1.042e-07 ***
## txblueberry:appt_catpost 8.344e-09 ***
## txplacebo:appt_catpost   1.682e-07 ***
## ---
## Signif. codes:  0 '***' 0.001 '**' 0.01 '*' 0.05 '.' 0.1 ' ' 1
##
##   Confidence level: 95%
##   Degrees of freedom method: Satterthwaite
```

```
ls_means(crp_mem_int, pairwise = TRUE)
```

```
## Least Squares Means table:
##
##                                                      Estimate Std.
```

```
Error    df
## txblueberry - txplacebo                            0.5682010  0.54
62717 121.1
## arm1 - arm2                                        0.6822373  0.54
70112 121.1
## bb_first0 - bb_first1                              1.0214839  1.35
20422  29.7
## appt_catbl - appt_catmid                          -0.1987168  0.66
43205 119.6
## appt_catbl - appt_catpost                         -0.2380559  0.64
23865 118.4
## appt_catmid - appt_catpost                        -0.0393390  0.66
15086 119.0
## txblueberry:appt_catbl - txplacebo:appt_catbl      0.9336673  0.92
17962 120.3
## txblueberry:appt_catbl - txblueberry:appt_catmid   0.2645524  0.92
67191 120.0
## txblueberry:appt_catbl - txplacebo:appt_catmid     0.2716813  0.95
19494 120.4
## txblueberry:appt_catbl - txblueberry:appt_catpost -0.1531256  0.92
63281 119.5
## txblueberry:appt_catbl - txplacebo:appt_catpost    0.6106812  0.89
59394 119.0
## txplacebo:appt_catbl - txblueberry:appt_catmid    -0.6691149  0.93
41740 119.5
## txplacebo:appt_catbl - txplacebo:appt_catmid      -0.6619860  0.95
70119 119.8
## txplacebo:appt_catbl - txblueberry:appt_catpost   -1.0867929  0.93
37023 119.3
## txplacebo:appt_catbl - txplacebo:appt_catpost     -0.3229861  0.90
48690 119.5
## txblueberry:appt_catmid - txplacebo:appt_catmid    0.0071289  0.96
88001 120.3
## txblueberry:appt_catmid - txblueberry:appt_catpost -0.4176780  0.93
64013 118.6
## txblueberry:appt_catmid - txplacebo:appt_catpost   0.3461288  0.92
26498 120.4
## txplacebo:appt_catmid - txblueberry:appt_catpost  -0.4248069  0.97
00442 120.3
## txplacebo:appt_catmid - txplacebo:appt_catpost     0.3389999  0.93
31995 119.1
## txblueberry:appt_catpost - txplacebo:appt_catpost  0.7638068  0.92
25123 120.2
##                                                   t value      low
er
## txblueberry - txplacebo                            1.0401 -0.51327
```

```
99
## arm1 - arm2                                          1.2472 -0.40070
70
## bb_first0 - bb_first1                                0.7555 -1.74099
63
## appt_catbl - appt_catmid                            -0.2991 -1.51407
24
## appt_catbl - appt_catpost                           -0.3706 -1.51011
02
## appt_catmid - appt_catpost                          -0.0595 -1.34919
57
## txblueberry:appt_catbl - txplacebo:appt_catbl        1.0129 -0.89138
10
## txblueberry:appt_catbl - txblueberry:appt_catmid     0.2855 -1.57028
81
## txblueberry:appt_catbl - txplacebo:appt_catmid       0.2854 -1.61304
57
## txblueberry:appt_catbl - txblueberry:appt_catpost  -0.1653 -1.98727
28
## txblueberry:appt_catbl - txplacebo:appt_catpost      0.6816 -1.16336
46
## txplacebo:appt_catbl - txblueberry:appt_catmid      -0.7163 -2.51878
96
## txplacebo:appt_catbl - txplacebo:appt_catmid        -0.6917 -2.55683
48
## txplacebo:appt_catbl - txblueberry:appt_catpost     -1.1640 -2.93556
35
## txplacebo:appt_catbl - txplacebo:appt_catpost       -0.3569 -2.11463
74
## txblueberry:appt_catmid - txplacebo:appt_catmid      0.0074 -1.91098
00
## txblueberry:appt_catmid - txblueberry:appt_catpost -0.4460 -2.27190
75
## txblueberry:appt_catmid - txplacebo:appt_catpost     0.3751 -1.48059
10
## txplacebo:appt_catmid - txblueberry:appt_catpost    -0.4379 -2.34537
79
## txplacebo:appt_catmid - txplacebo:appt_catpost       0.3633 -1.50881
27
## txblueberry:appt_catpost - txplacebo:appt_catpost    0.8280 -1.06267
41
##                                                          upper Pr(>|
t|)
## txblueberry - txplacebo                              1.6496819   0.3
003
## arm1 - arm2                                          1.7651816   0.2
```

```
147
## bb_first0 - bb_first1                                 3.7839642   0.4
559
## appt_catbl - appt_catmid                              1.1166387   0.7
654
## appt_catbl - appt_catpost                             1.0339984   0.7
116
## appt_catmid - appt_catpost                            1.2705176   0.9
527
## txblueberry:appt_catbl - txplacebo:appt_catbl         2.7587156   0.3
132
## txblueberry:appt_catbl - txblueberry:appt_catmid      2.0993929   0.7
758
## txblueberry:appt_catbl - txplacebo:appt_catmid        2.1564082   0.7
758
## txblueberry:appt_catbl - txblueberry:appt_catpost     1.6810216   0.8
690
## txblueberry:appt_catbl - txplacebo:appt_catpost       2.3847270   0.4
968
## txplacebo:appt_catbl - txblueberry:appt_catmid        1.1805597   0.4
752
## txplacebo:appt_catbl - txplacebo:appt_catmid          1.2328628   0.4
905
## txplacebo:appt_catbl - txblueberry:appt_catpost       0.7619776   0.2
468
## txplacebo:appt_catbl - txplacebo:appt_catpost         1.4686652   0.7
218
## txblueberry:appt_catmid - txplacebo:appt_catmid       1.9252378   0.9
941
## txblueberry:appt_catmid - txblueberry:appt_catpost  1.4365515   0.6
564
## txblueberry:appt_catmid - txplacebo:appt_catpost      2.1728486   0.7
082
## txplacebo:appt_catmid - txblueberry:appt_catpost      1.4957641   0.6
622
## txplacebo:appt_catmid - txplacebo:appt_catpost        2.1868126   0.7
170
## txblueberry:appt_catpost - txplacebo:appt_catpost     2.5902877   0.4
093
##
##   Confidence level: 95%
##   Degrees of freedom method: Satterthwaite
```

### EPR Modeling and Evaluation

```
epr_mem_int <- lmer(epr ~ tx +
                       arm +
```

```
                                tx : appt_cat +
                                bb_first +
                                appt_cat +
                                (1 | de_id),
                              data = bbd_polished_factors)
print(epr_mem_int)
```

```
## Linear mixed model fit by REML ['lmerModLmerTest']
## Formula: epr ~ tx + arm + tx:appt_cat + bb_first + appt_cat + (1 |
de_id)
##    Data: bbd_polished_factors
## REML criterion at convergence: 56.2315
## Random effects:
##  Groups   Name        Std.Dev.
##  de_id    (Intercept) 0.07409
##  Residual             0.25738
## Number of obs: 161, groups:  de_id, 31
## Fixed Effects:
##            (Intercept)                txplacebo                     a
rm2
##               0.276764                -0.013716                 0.041
558
##              bb_first1              appt_catmid             appt_catp
ost
##              -0.014866                 0.027264                -0.009
649
##  txplacebo:appt_catmid  txplacebo:appt_catpost
##              -0.117603                -0.060108
```

```
plot(epr_mem_int)
```

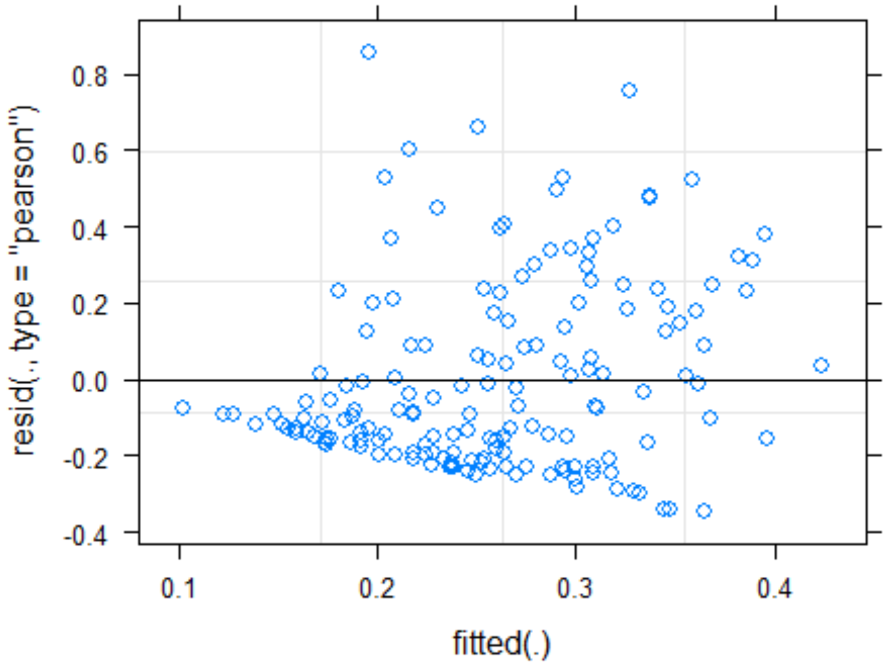


```
summary(epr_mem_int)

## Linear mixed model fit by REML. t-tests use Satterthwaite's method
[
## lmerModLmerTest]
## Formula: epr ~ tx + arm + tx:appt_cat + bb_first + appt_cat + (1 |
de_id)
##    Data: bbd_polished_factors
##
## REML criterion at convergence: 56.2
##
## Scaled residuals:
##     Min      1Q  Median      3Q     Max
## -1.3431 -0.6884 -0.3556  0.5803  3.3258
##
## Random effects:
##  Groups   Name        Variance Std.Dev.
##  de_id    (Intercept) 0.005489 0.07409
##  Residual             0.066245 0.25738
## Number of obs: 161, groups:  de_id, 31
##
## Fixed effects:
##                            Estimate Std. Error         df t value Pr(
>|t|)
## (Intercept)                0.276764   0.059474 137.533687   4.654 7.5
```

```
9e-06 ***
## txplacebo                -0.013716   0.068546 128.394487  -0.200
0.842
## arm2                      0.041558   0.041148 134.771462   1.010
0.314
## bb_first1                -0.014866   0.049073  28.519211  -0.303
0.764
## appt_catmid               0.027264   0.071035 128.717901   0.384
0.702
## appt_catpost             -0.009649   0.070297 128.088162  -0.137
0.891
## txplacebo:appt_catmid    -0.117603   0.099416 127.312527  -1.183
0.239
## txplacebo:appt_catpost   -0.060108   0.098458 127.417096  -0.610
0.543
## ---
## Signif. codes:  0 '***' 0.001 '**' 0.01 '*' 0.05 '.' 0.1 ' ' 1
##
## Correlation of Fixed Effects:
##                   (Intr) txplcb arm2   bb_fr1 appt_ctm appt_ctp txplcb
:ppt_ctm
## txplacebo         -0.620
## arm2              -0.338  0.050
## bb_first1         -0.400  0.047 -0.009
## appt_catmid       -0.561  0.487 -0.011  0.009
## appt_catpst       -0.567  0.492  0.024 -0.022  0.477
## txplcb:ppt_ctm     0.403 -0.684  0.004 -0.009 -0.713   -0.340
## txplcb:ppt_ctp     0.413 -0.692 -0.030  0.005 -0.340   -0.713    0.477
```

```
ls_means(epr_mem_int)
```

```
## Least Squares Means table:
##
##                            Estimate Std. Error    df t value    lower
upper
## txblueberry                0.295982   0.032164  71.0  9.2023 0.231849
0.360115
## txplacebo                  0.223029   0.031897  70.9  6.9921 0.159426
0.286632
## arm1                       0.238727   0.030816  67.5  7.7469 0.177227
0.300226
## arm2                       0.280284   0.033193  74.1  8.4441 0.214147
0.346421
## bb_first0                  0.266938   0.033422  29.6  7.9869 0.198640
0.335237
## bb_first1                  0.252073   0.035942  27.7  7.0133 0.178407
```

```
0.325738
## appt_catbl              0.283252   0.036791 106.0  7.6989 0.210310
0.356195
## appt_catmid             0.251715   0.038751 112.0  6.4957 0.174934
0.328496
## appt_catpost            0.243549   0.038018 110.4  6.4062 0.168210
0.318888
## txblueberry:appt_catbl   0.290110   0.050576 149.7  5.7361 0.190175
0.390046
## txplacebo:appt_catbl     0.276394   0.049985 149.3  5.5295 0.177624
0.375165
## txblueberry:appt_catmid  0.317375   0.053488 150.7  5.9335 0.211691
0.423059
## txplacebo:appt_catmid    0.186056   0.052671 150.3  3.5324 0.081984
0.290127
## txblueberry:appt_catpost 0.280461   0.052499 150.4  5.3422 0.176729
0.384193
## txplacebo:appt_catpost   0.206637   0.051612 149.9  4.0037 0.104656
0.308617
##                           Pr(>|t|)
## txblueberry              9.911e-14 ***
## txplacebo                1.222e-09 ***
## arm1                     6.571e-11 ***
## arm2                     1.860e-12 ***
## bb_first0                7.201e-09 ***
## bb_first1                1.346e-07 ***
## appt_catbl               7.595e-12 ***
## appt_catmid              2.359e-09 ***
## appt_catpost             3.765e-09 ***
## txblueberry:appt_catbl   5.190e-08 ***
## txplacebo:appt_catbl     1.399e-07 ***
## txblueberry:appt_catmid  1.956e-08 ***
## txplacebo:appt_catmid    0.0005472 ***
## txblueberry:appt_catpost 3.334e-07 ***
## txplacebo:appt_catpost   9.779e-05 ***
## ---
## Signif. codes:  0 '***' 0.001 '**' 0.01 '*' 0.05 '.' 0.1 ' ' 1
##
##   Confidence level: 95%
##   Degrees of freedom method: Satterthwaite
```

```
ls_means(epr_mem_int, pairwise = TRUE)
```

```
## Least Squares Means table:
##
##                                                        Estimate Std.
```

```
Error    df
## txblueberry - txplacebo                             0.0729530  0.04
11626 134.9
## arm1 - arm2                                        -0.0415576  0.04
11484 134.8
## bb_first0 - bb_first1                               0.0148656  0.04
90730  28.5
## appt_catbl - appt_catmid                            0.0315371  0.04
98182 130.0
## appt_catbl - appt_catpost                           0.0397037  0.04
92732 128.4
## appt_catmid - appt_catpost                          0.0081665  0.05
07013 129.4
## txblueberry:appt_catbl - txplacebo:appt_catbl       0.0137159  0.06
85462 128.4
## txblueberry:appt_catbl - txblueberry:appt_catmid   -0.0272644  0.07
10350 128.7
## txblueberry:appt_catbl - txplacebo:appt_catmid      0.1040545  0.07
05599 130.8
## txblueberry:appt_catbl - txblueberry:appt_catpost   0.0096494  0.07
02971 128.1
## txblueberry:appt_catbl - txplacebo:appt_catpost     0.0834738  0.06
97718 130.1
## txplacebo:appt_catbl - txblueberry:appt_catmid     -0.0409803  0.07
07506 130.8
## txplacebo:appt_catbl - txplacebo:appt_catmid        0.0903386  0.06
97104 128.6
## txplacebo:appt_catbl - txblueberry:appt_catpost    -0.0040665  0.06
99833 129.8
## txplacebo:appt_catbl - txplacebo:appt_catpost       0.0697579  0.06
90003 127.8
## txblueberry:appt_catmid - txplacebo:appt_catmid     0.1313189  0.07
25523 130.5
## txblueberry:appt_catmid - txblueberry:appt_catpost  0.0369138  0.07
22990 127.3
## txblueberry:appt_catmid - txplacebo:appt_catpost    0.1107381  0.07
19547 132.6
## txplacebo:appt_catmid - txblueberry:appt_catpost   -0.0944051  0.07
18898 130.9
## txplacebo:appt_catmid - txplacebo:appt_catpost     -0.0205808  0.07
09373 128.8
## txblueberry:appt_catpost - txplacebo:appt_catpost   0.0738243  0.07
11233 130.4
##                                                   t value      low
er
## txblueberry - txplacebo                             1.7723 -0.00845
```

```
46
## arm1 - arm2                                       -1.0099 -0.12293
77
## bb_first0 - bb_first1                              0.3029 -0.08557
35
## appt_catbl - appt_catmid                           0.6330 -0.06702
23
## appt_catbl - appt_catpost                          0.8058 -0.05778
86
## appt_catmid - appt_catpost                         0.1611 -0.09214
45
## txblueberry:appt_catbl - txplacebo:appt_catbl       0.2001 -0.12191
06
## txblueberry:appt_catbl - txblueberry:appt_catmid   -0.3838 -0.16781
17
## txblueberry:appt_catbl - txplacebo:appt_catmid      1.4747 -0.03553
13
## txblueberry:appt_catbl - txblueberry:appt_catpost   0.1373 -0.12944
45
## txblueberry:appt_catbl - txplacebo:appt_catpost     1.1964 -0.05456
03
## txplacebo:appt_catbl - txblueberry:appt_catmid     -0.5792 -0.18094
36
## txplacebo:appt_catbl - txplacebo:appt_catmid        1.2959 -0.04758
90
## txplacebo:appt_catbl - txblueberry:appt_catpost    -0.0581 -0.14252
24
## txplacebo:appt_catbl - txplacebo:appt_catpost       1.0110 -0.06677
34
## txblueberry:appt_catmid - txplacebo:appt_catmid     1.8100 -0.01221
18
## txblueberry:appt_catmid - txblueberry:appt_catpost  0.5106 -0.10614
93
## txblueberry:appt_catmid - txplacebo:appt_catpost    1.5390 -0.03158
92
## txplacebo:appt_catmid - txblueberry:appt_catpost   -1.3132 -0.23662
14
## txplacebo:appt_catmid - txplacebo:appt_catpost     -0.2901 -0.16093
44
## txblueberry:appt_catpost - txplacebo:appt_catpost   1.0380 -0.06688
06
##                                                        upper Pr(>|
t|)
## txblueberry - txplacebo                            0.1543606   0.0
786 .
## arm1 - arm2                                        0.0398225   0.3
```

```
143
## bb_first0 - bb_first1                                0.1153048   0.7
641
## appt_catbl - appt_catmid                             0.1300965   0.5
278
## appt_catbl - appt_catpost                            0.1371959   0.4
219
## appt_catmid - appt_catpost                           0.1084776   0.8
723
## txblueberry:appt_catbl - txplacebo:appt_catbl       0.1493424   0.8
417
## txblueberry:appt_catbl - txblueberry:appt_catmid    0.1132829   0.7
017
## txblueberry:appt_catbl - txplacebo:appt_catmid      0.2436404   0.1
427
## txblueberry:appt_catbl - txblueberry:appt_catpost   0.1487434   0.8
910
## txblueberry:appt_catbl - txplacebo:appt_catpost     0.2215078   0.2
337
## txplacebo:appt_catbl - txblueberry:appt_catmid      0.0989830   0.5
634
## txplacebo:appt_catbl - txplacebo:appt_catmid        0.2282663   0.1
973
## txplacebo:appt_catbl - txblueberry:appt_catpost     0.1343895   0.9
538
## txplacebo:appt_catbl - txplacebo:appt_catpost       0.2062891   0.3
139
## txblueberry:appt_catmid - txplacebo:appt_catmid     0.2748496   0.0
726 .
## txblueberry:appt_catmid - txblueberry:appt_catpost  0.1799769   0.6
105
## txblueberry:appt_catmid - txplacebo:appt_catpost    0.2530655   0.1
262
## txplacebo:appt_catmid - txblueberry:appt_catpost    0.0478112   0.1
914
## txplacebo:appt_catmid - txplacebo:appt_catpost      0.1197729   0.7
722
## txblueberry:appt_catpost - txplacebo:appt_catpost   0.2145292   0.3
012
## ---
## Signif. codes:  0 '***' 0.001 '**' 0.01 '*' 0.05 '.' 0.1 ' ' 1
##
##   Confidence level: 95%
##   Degrees of freedom method: Satterthwaite
```

## MSD & HDRS Correlations

```
ggplot(data = filter(outlier_il1b, appt_cat != "mid"),
       mapping = aes(
         x = il1b_2,
         y = HDRS_num)) +
  geom_point(size = .5) +
  stat_cor(method = "pearson",
           cor.coef.name = "R",
           size = 2.25)

## Warning: Removed 8 rows containing non-finite values (`stat_cor()`)
.

## Warning: Removed 8 rows containing missing values (`geom_point()`).
```

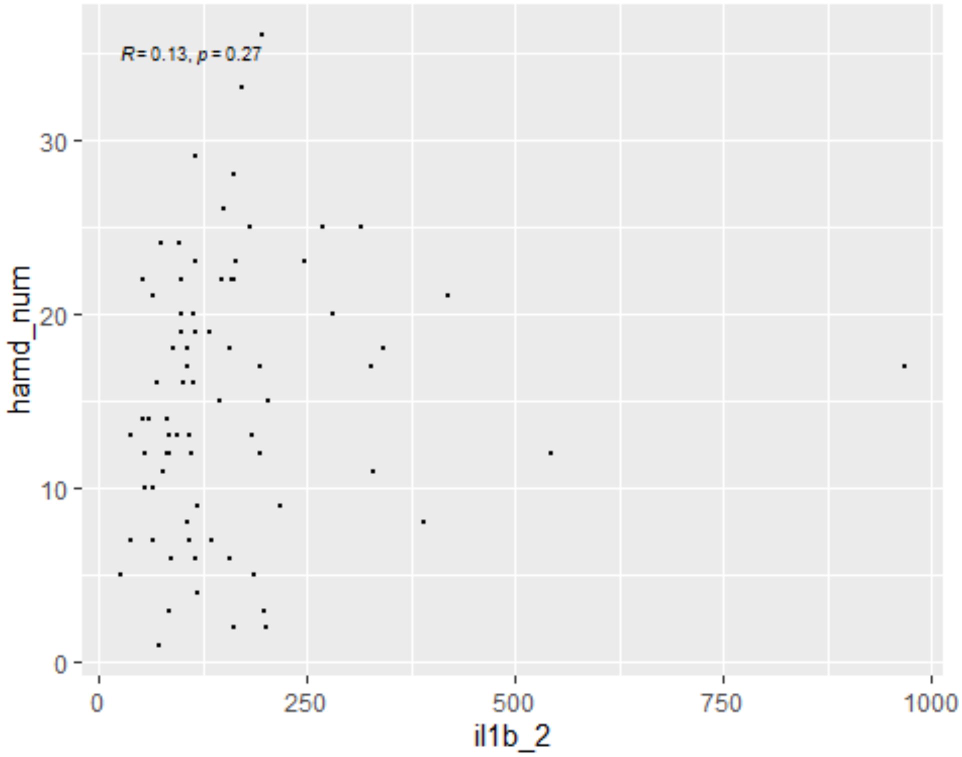


```
ggplot(data = filter(outlier_il6, appt_cat != "mid"),
       mapping = aes(
         x = il6_2,
         y = HDRS_num)) +
  geom_point(size = .5) +
  stat_cor(method = "pearson",
           cor.coef.name = "R",
           size = 2.25)
```

```
## Warning: Removed 8 rows containing non-finite values (`stat_cor()`)
.
## Removed 8 rows containing missing values (`geom_point()`).
```

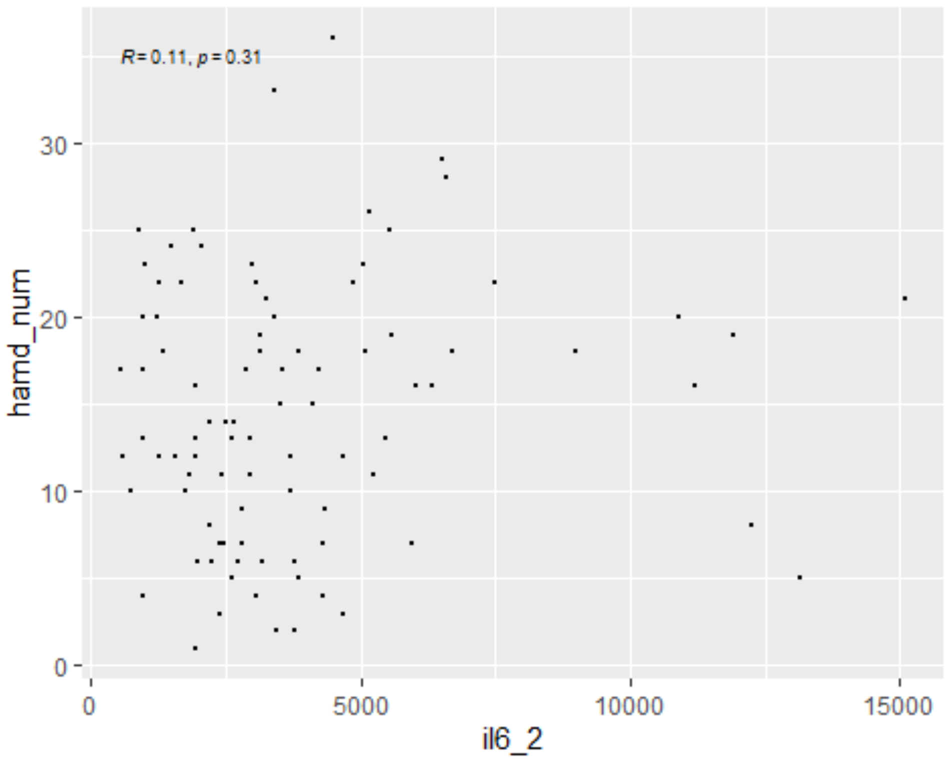


```
ggplot(data =  filter(outlier_tnfa, appt_cat != "mid"),
       mapping = aes(
         x = tnfa_2,
         y = HDRS_num)) +
  geom_point(size = .5) +
  stat_cor(method = "pearson",
           cor.coef.name = "R",
           size = 2.25)
```

```
## Warning: Removed 8 rows containing non-finite values (`stat_cor()`)
.
## Removed 8 rows containing missing values (`geom_point()`).
```

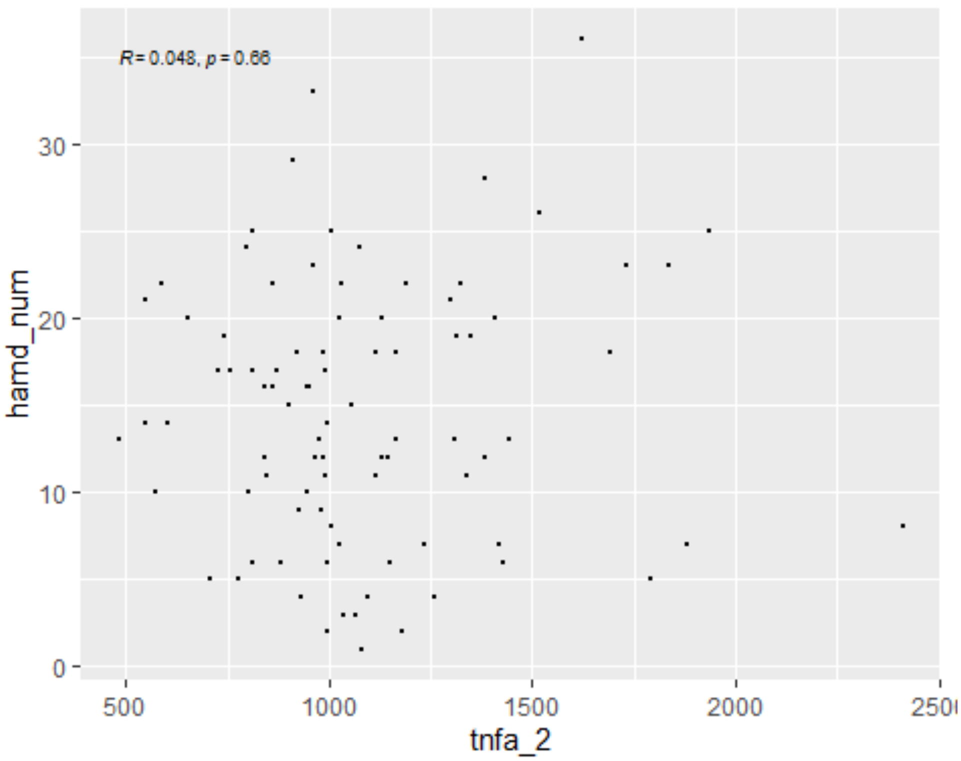


```
ggplot(data =  filter(outlier_ifny, appt_cat != "mid"),
       mapping = aes(
         x = ifny_2,
         y = HDRS_num)) +
  geom_point(size = .5) +
  stat_cor(method = "pearson",
           cor.coef.name = "R",
           size = 2.25)

## Warning: Removed 8 rows containing non-finite values (`stat_cor()`)
.
## Removed 8 rows containing missing values (`geom_point()`).
```

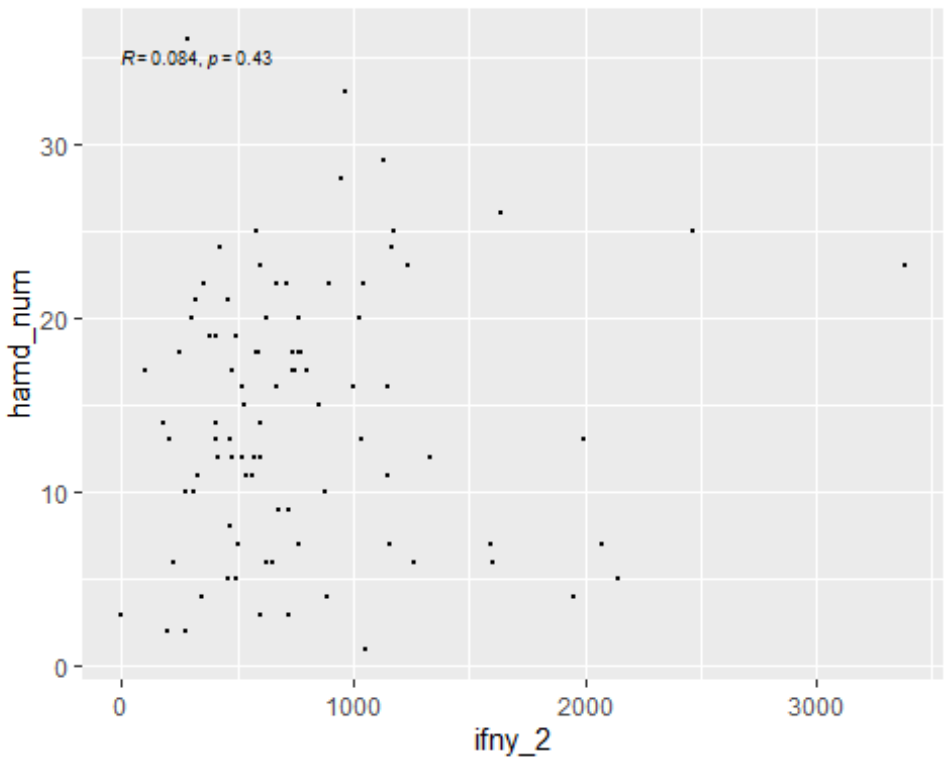


```
ggplot(data =  filter(outlier_il10, appt_cat != "mid"),
       mapping = aes(
         x = il10_2,
         y = HDRS_num)) +
  geom_point(size = .5) +
  stat_cor(method = "pearson",
           cor.coef.name = "R",
           size = 2.25)

## Warning: Removed 7 rows containing non-finite values (`stat_cor()`)
.

## Warning: Removed 7 rows containing missing values (`geom_point()`).
```

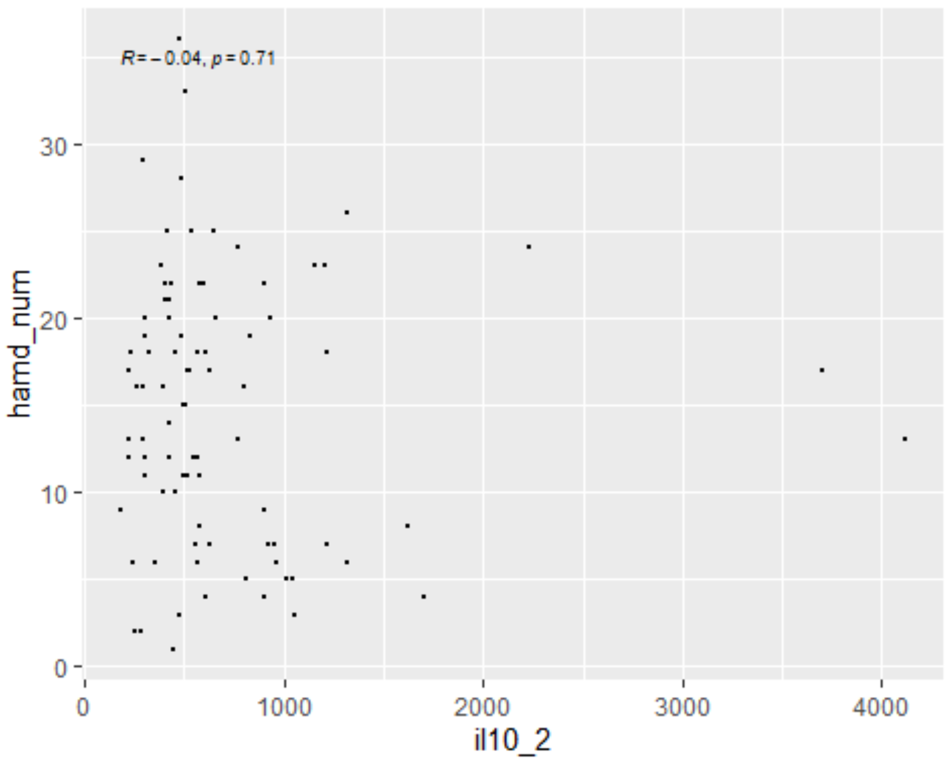


## MSD & GAD-7 Correlations

```
ggplot(data = outlier_il1b,
       mapping = aes(
         x = il1b_2,
         y = GAD-7_num)) +
  geom_point(size = .5) +
  stat_cor(method = "pearson",
           cor.coef.name = "R",
           size = 2.25)

## Warning: Removed 3 rows containing non-finite values (`stat_cor()`)
.

## Warning: Removed 3 rows containing missing values (`geom_point()`).
```

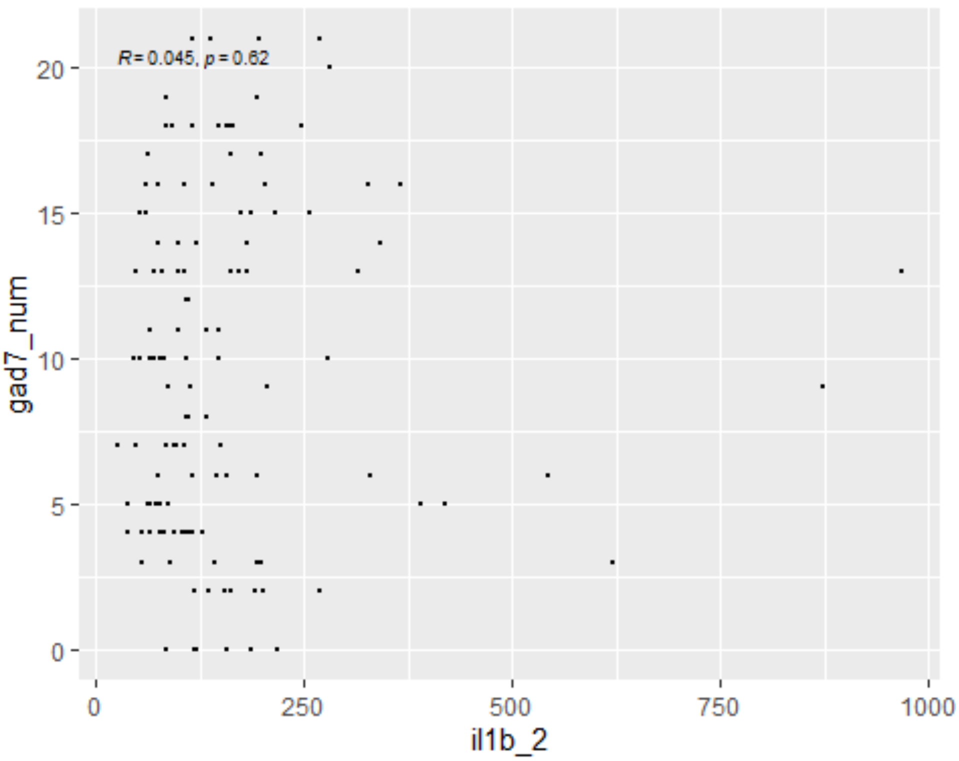


```
ggplot(data = outlier_il6,
       mapping = aes(
         x = il6_2,
         y = GAD-7_num)) +
  geom_point(size = .5) +
  stat_cor(method = "pearson",
           cor.coef.name = "R",
           size = 2.25)

## Warning: Removed 3 rows containing non-finite values (`stat_cor()`)
.
## Removed 3 rows containing missing values (`geom_point()`).
```

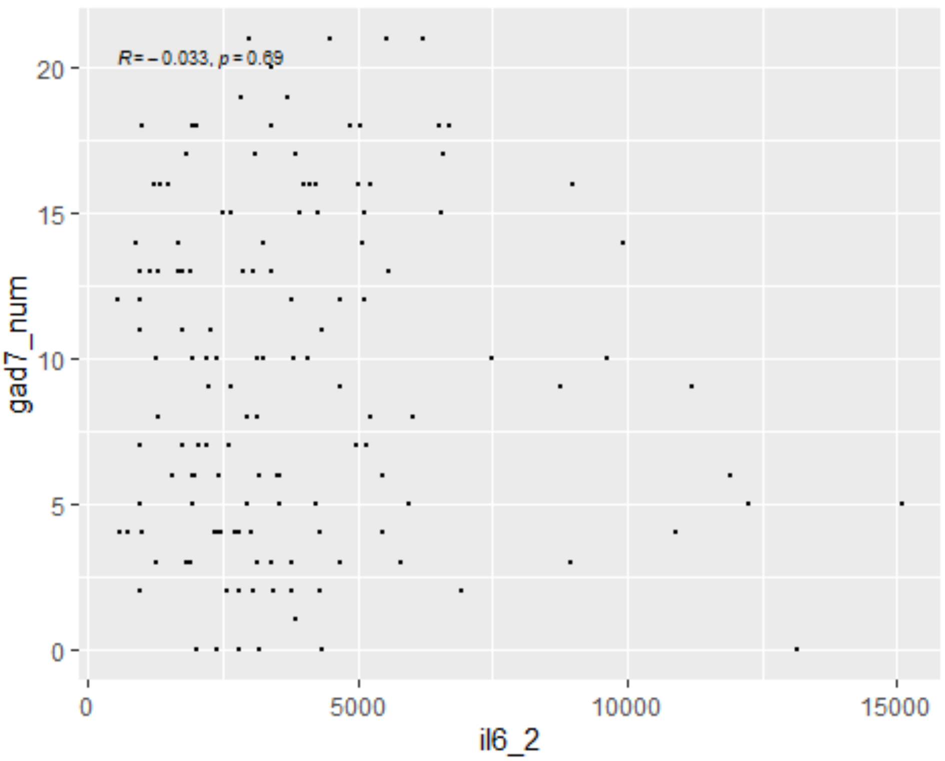


```
ggplot(data = outlier_tnfa,
       mapping = aes(
         x = tnfa_2,
         y = GAD-7_num)) +
  geom_point(size = .5) +
  stat_cor(method = "pearson",
           cor.coef.name = "R",
           size = 2.25)

## Warning: Removed 3 rows containing non-finite values (`stat_cor()`)
.
## Removed 3 rows containing missing values (`geom_point()`).
```

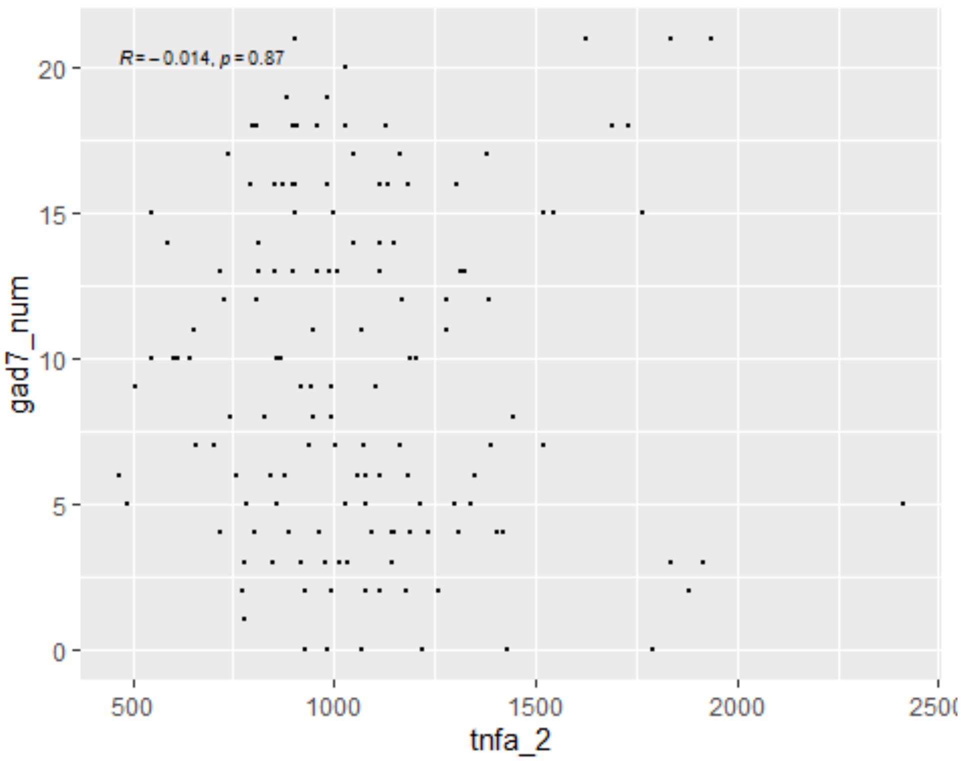


```
ggplot(data = outlier_ifny,
       mapping = aes(
         x = ifny_2,
         y = GAD-7_num)) +
  geom_point(size = .5) +
  stat_cor(method = "pearson",
           cor.coef.name = "R",
           size = 2.25)

## Warning: Removed 3 rows containing non-finite values (`stat_cor()`)
.
## Removed 3 rows containing missing values (`geom_point()`).
```

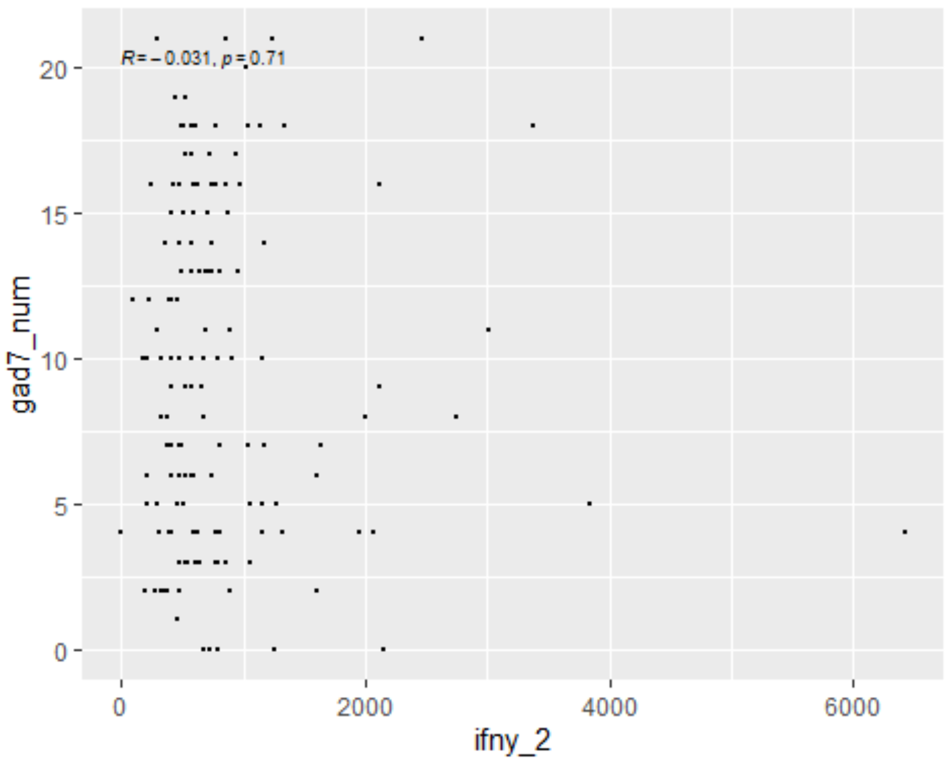


```r
ggplot(data = outlier_il10,
       mapping = aes(
         x = il10_2,
         y = GAD-7_num)) +
  geom_point(size = .5) +
  stat_cor(method = "pearson",
           cor.coef.name = "R",
           size = 2.25)
```

```
## Warning: Removed 2 rows containing non-finite values (`stat_cor()`)
.

## Warning: Removed 2 rows containing missing values (`geom_point()`).
```

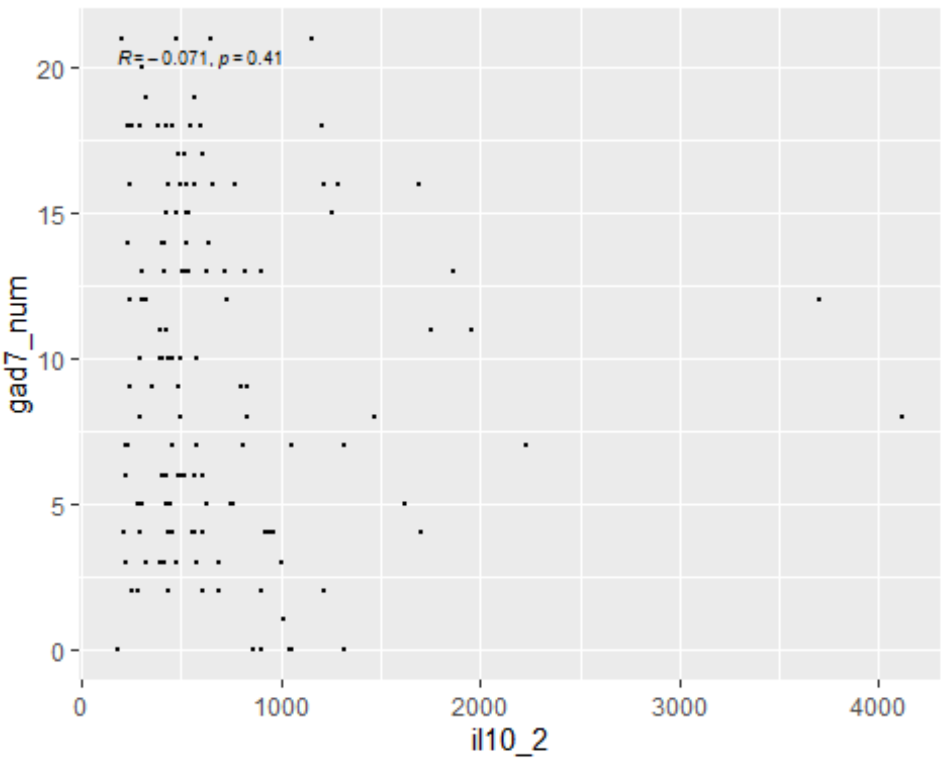


```
ggplot(data = bbd_polished,
       mapping = aes(
         x = crp,
         y = GAD-7_num)) +
  geom_point(size = .5) +
  stat_cor(method = "pearson",
           cor.coef.name = "R",
           size = 2.25)

## Warning: Removed 118 rows containing non-finite values (`stat_cor()
`).

## Warning: Removed 118 rows containing missing values (`geom_point()`
).
```

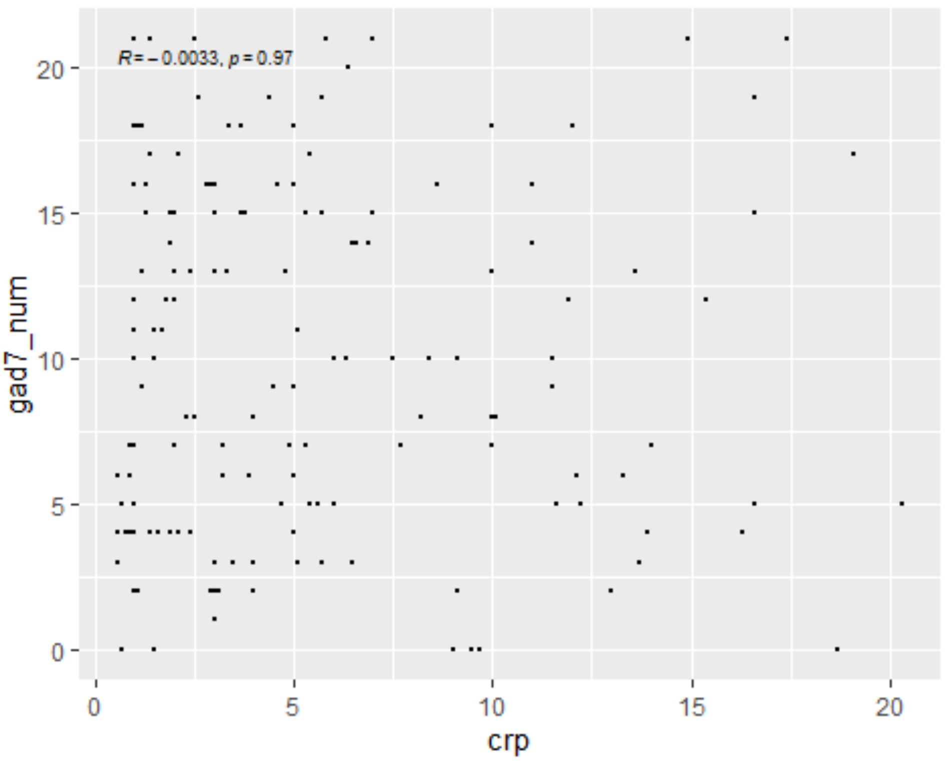


## MSD & MDI Correlations

```
ggplot(data = outlier_il1b,
       mapping = aes(
         x = il1b_2,
         y = mdi_num)) +
  geom_point(size = .5) +
  stat_cor(method = "pearson",
           cor.coef.name = "R",
           size = 2.25)

## Warning: Removed 3 rows containing non-finite values (`stat_cor()`)
.

## Warning: Removed 3 rows containing missing values (`geom_point()`).
```

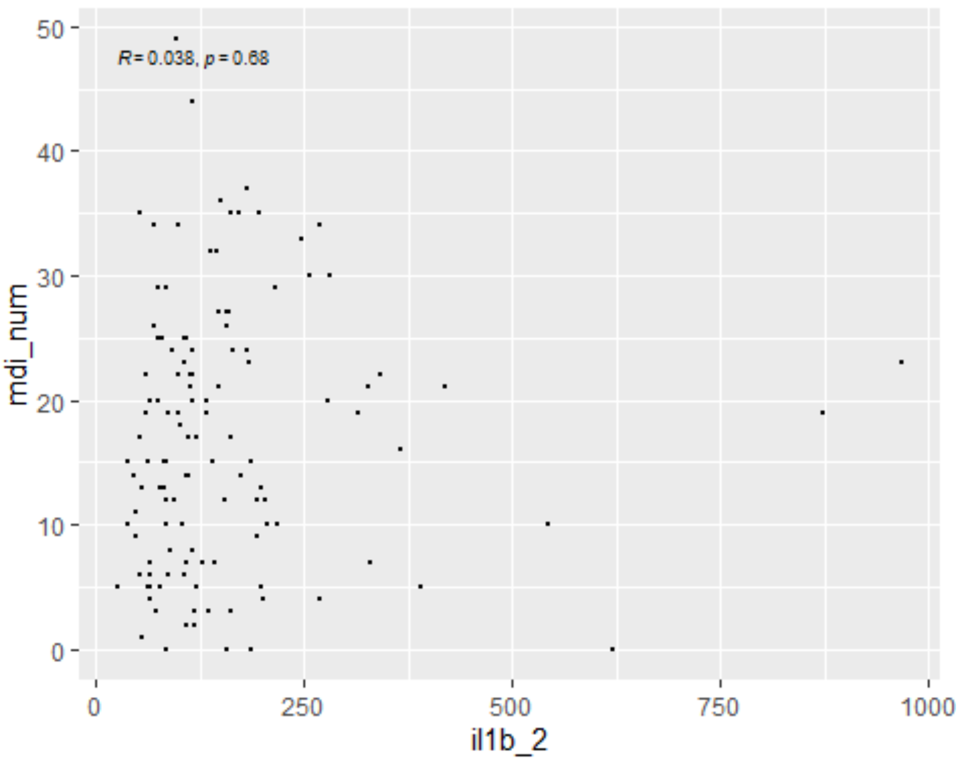


```
ggplot(data = outlier_il6,
       mapping = aes(
         x = il6_2,
         y = mdi_num)) +
  geom_point(size = .5) +
  stat_cor(method = "pearson",
           cor.coef.name = "R",
           size = 2.25)

## Warning: Removed 3 rows containing non-finite values (`stat_cor()`)
.
## Removed 3 rows containing missing values (`geom_point()`).
```

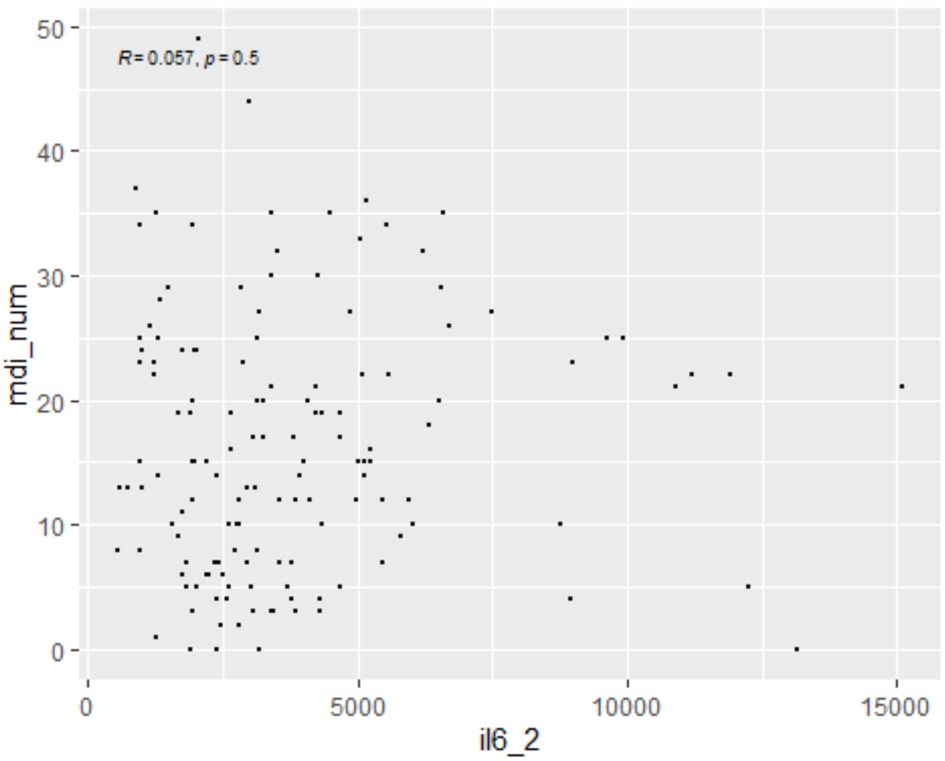


```
ggplot(data = outlier_tnfa,
       mapping = aes(
         x = tnfa_2,
         y = mdi_num)) +
  geom_point(size = .5) +
  stat_cor(method = "pearson",
           cor.coef.name = "R",
           size = 2.25)

## Warning: Removed 3 rows containing non-finite values (`stat_cor()`)
.
## Removed 3 rows containing missing values (`geom_point()`).
```

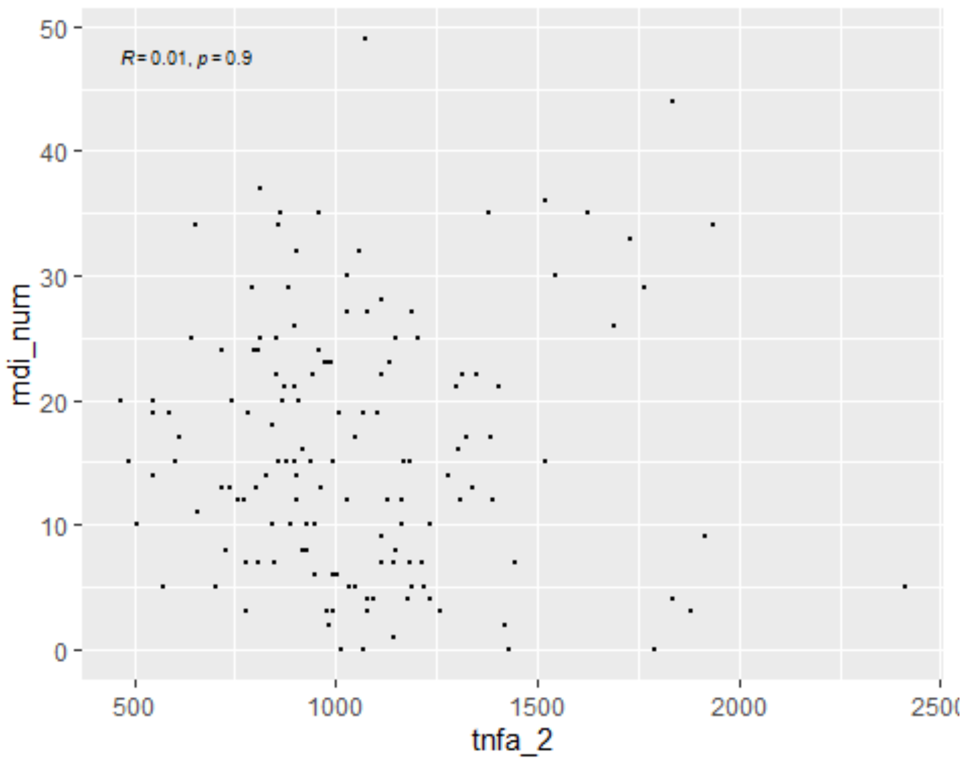


```
ggplot(data = outlier_ifny,
       mapping = aes(
         x = ifny_2,
         y = mdi_num)) +
  geom_point(size = .5) +
  stat_cor(method = "pearson",
           cor.coef.name = "R",
           size = 2.25)

## Warning: Removed 3 rows containing non-finite values (`stat_cor()`)
.
## Removed 3 rows containing missing values (`geom_point()`).
```

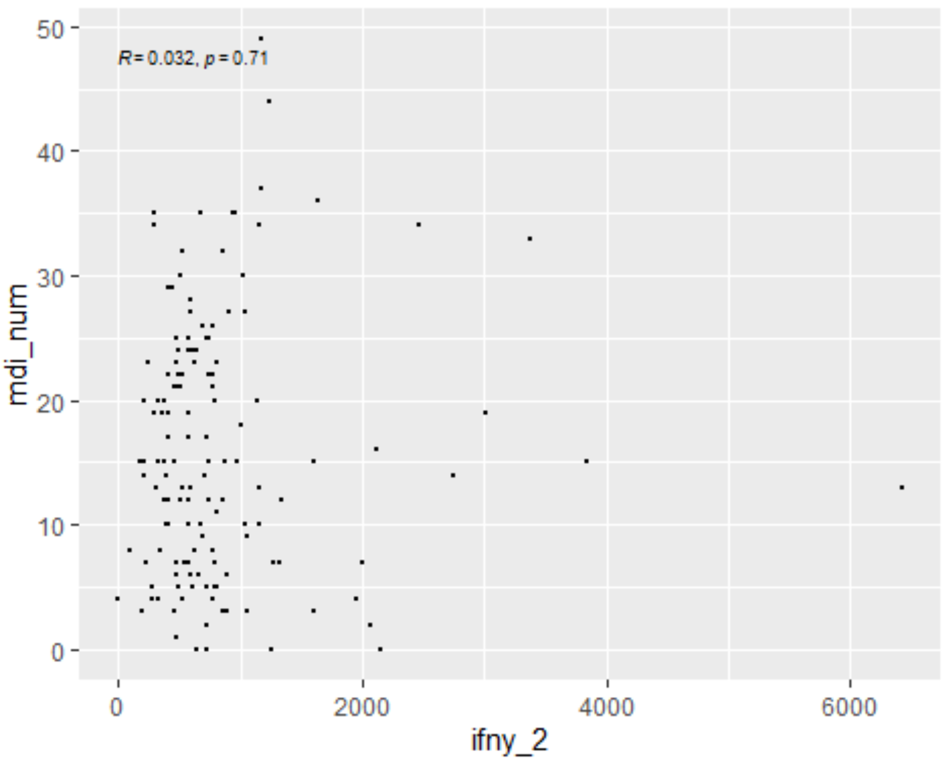


```
ggplot(data = outlier_il10,
       mapping = aes(
         x = il10_2,
         y = mdi_num)) +
  geom_point(size = .5) +
  stat_cor(method = "pearson",
           cor.coef.name = "R",
           size = 2.25)

## Warning: Removed 3 rows containing non-finite values (`stat_cor()`)
.
## Removed 3 rows containing missing values (`geom_point()`).
```

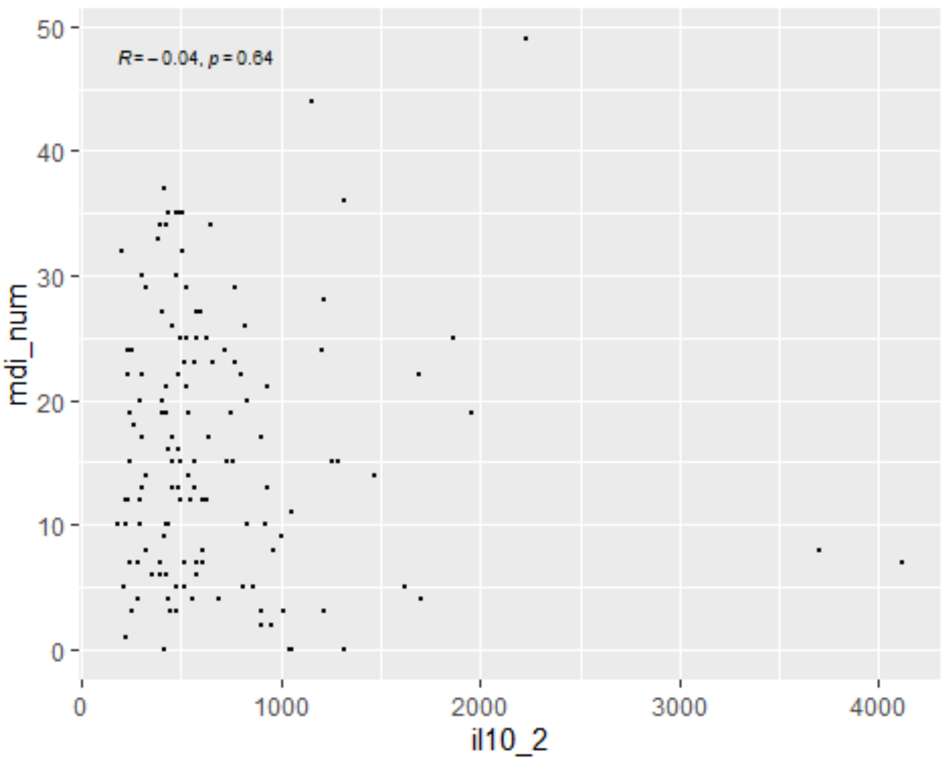


## CRP & EPR, Behavior Correlations

```
ggplot(data =  filter(bbd_polished, appt_cat != "mid"),
       mapping = aes(
         x = crp,
         y = HDRS_num)) +
  geom_point(size = .5) +
  stat_cor(method = "pearson",
           cor.coef.name = "R",
           size = 2.25)

## Warning: Removed 85 rows containing non-finite values (`stat_cor()`
).

## Warning: Removed 85 rows containing missing values (`geom_point()`)
.
```

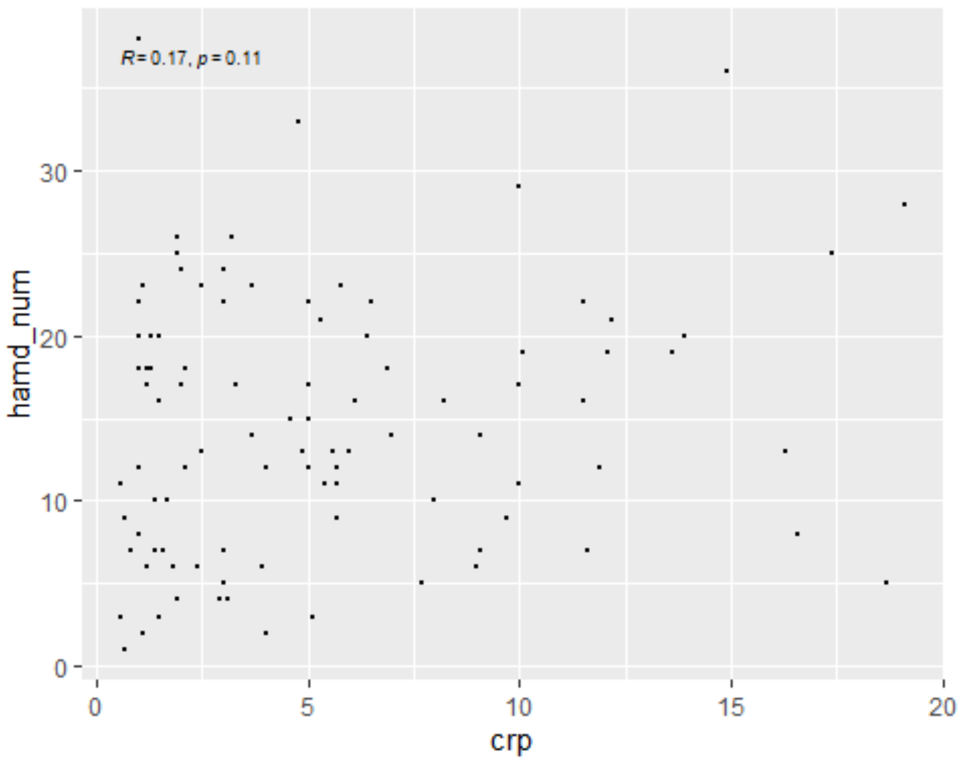


```
ggplot(data =  bbd_polished,
       mapping = aes(
         x = crp,
         y = GAD-7_num)) +
  geom_point(size = .5) +
  stat_cor(method = "pearson",
           cor.coef.name = "R",
           size = 2.25)

## Warning: Removed 118 rows containing non-finite values (`stat_cor()
`).

## Warning: Removed 118 rows containing missing values (`geom_point()`
).
```

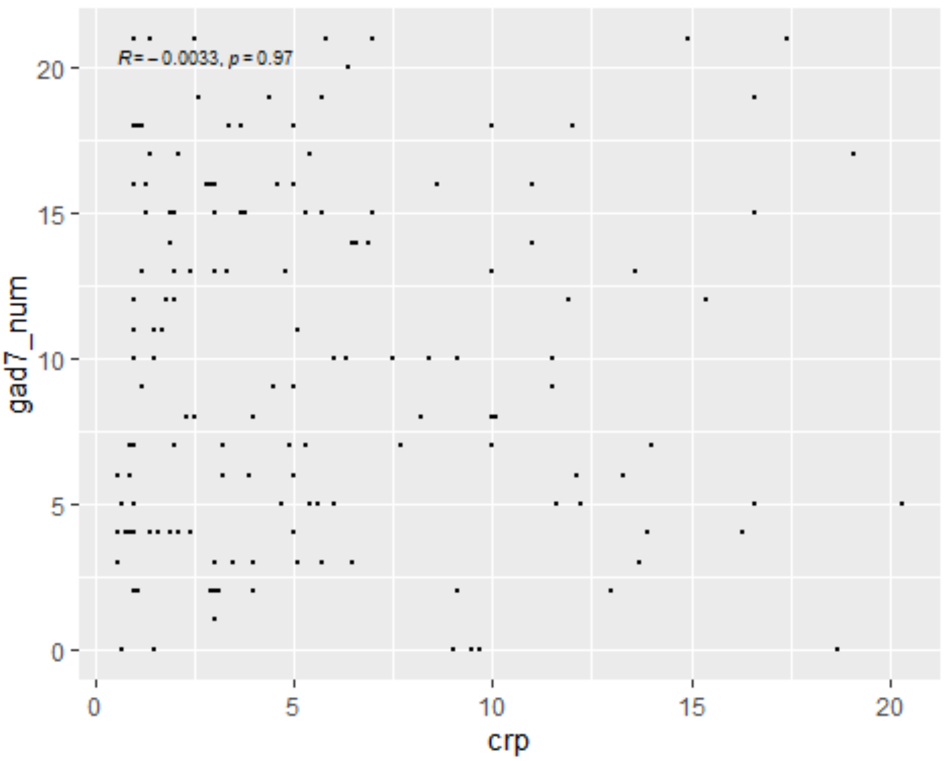


```
ggplot(data =  bbd_polished,
       mapping = aes(
         x = crp,
         y = mdi_num)) +
  geom_point(size = .5) +
  stat_cor(method = "pearson",
           cor.coef.name = "R",
           size = 2.25)

## Warning: Removed 120 rows containing non-finite values (`stat_cor()
`).

## Warning: Removed 120 rows containing missing values (`geom_point()`
).
```

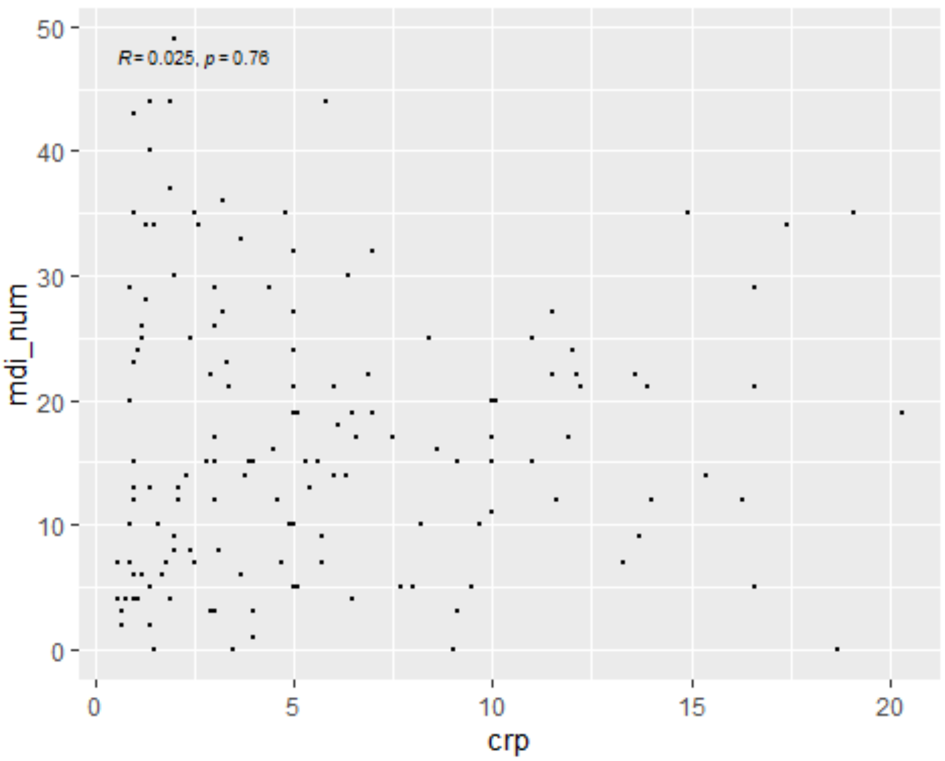


```
ggplot(data =  filter(bbd_polished, appt_cat != "mid"),
       mapping = aes(
         x = epr,
         y = HDRS_num)) +
  geom_point(size = .5) +
  stat_cor(method = "pearson",
           cor.coef.name = "R",
           size = 2.25)

## Warning: Removed 82 rows containing non-finite values (`stat_cor()`
).

## Warning: Removed 82 rows containing missing values (`geom_point()`)
.
```

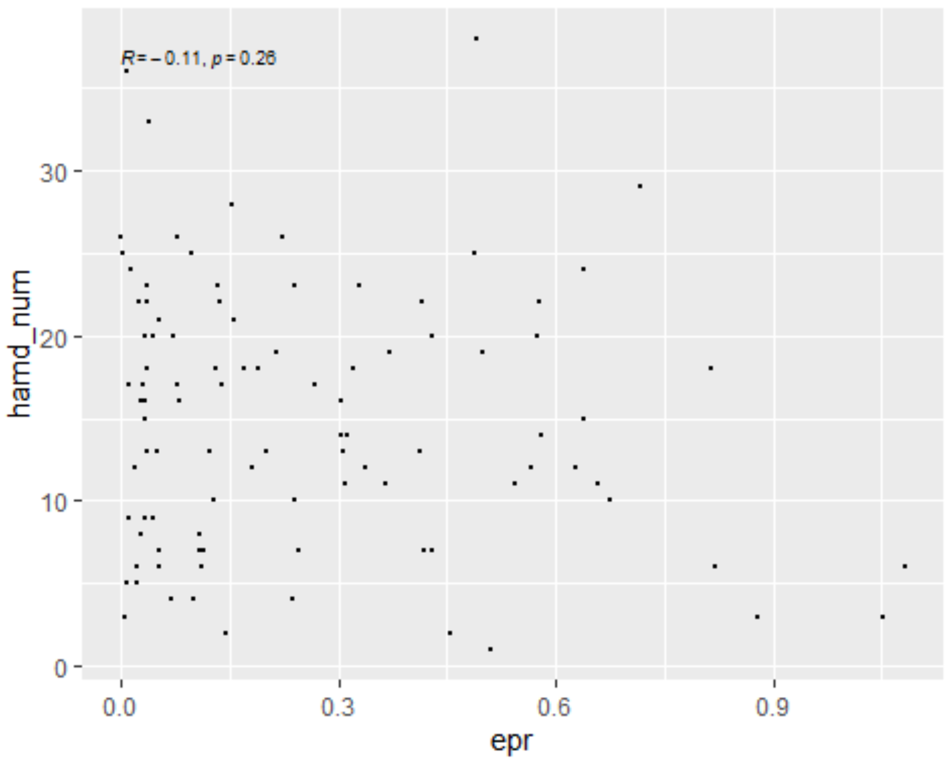


```
ggplot(data =  bbd_polished,
       mapping = aes(
         x = epr,
         y = GAD-7_num)) +
  geom_point(size = .5) +
  stat_cor(method = "pearson",
           cor.coef.name = "R",
           size = 2.25)

## Warning: Removed 113 rows containing non-finite values (`stat_cor()
`).

## Warning: Removed 113 rows containing missing values (`geom_point()`
).
```

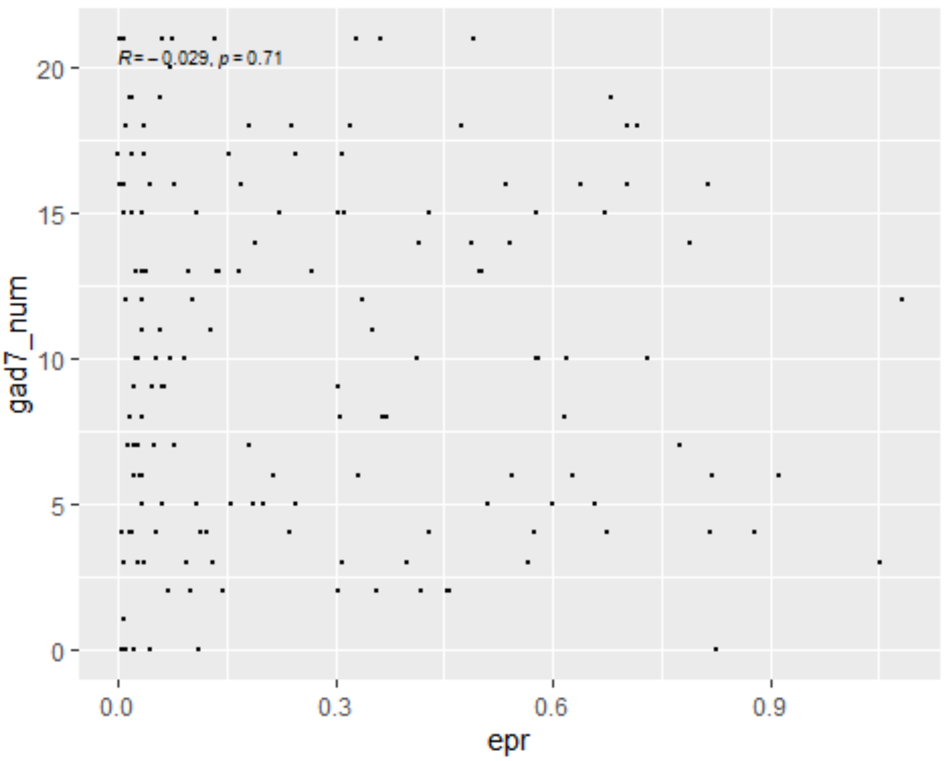


```r
ggplot(data =  bbd_polished,
       mapping = aes(
         x = epr,
         y = mdi_num)) +
  geom_point(size = .5) +
  stat_cor(method = "pearson",
           cor.coef.name = "R",
           size = 2.25)

## Warning: Removed 113 rows containing non-finite values (`stat_cor()
`).
## Removed 113 rows containing missing values (`geom_point()`).
```

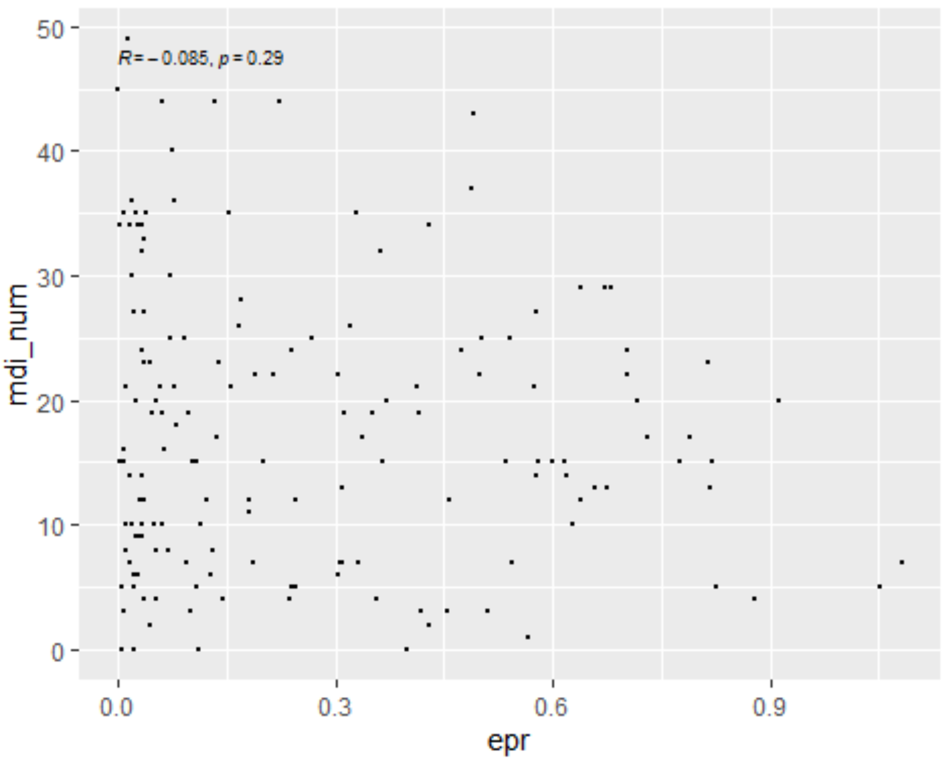
R = −0.085, p = 0.29
mdi_num
epr
0
10
20
30
40
50
0.0
0.3
0.6
0.9
