# Supplementary material for: Effects of Blueberry Supplementation on Depression and Anxiety Symptoms in a Rural Louisiana Population
Source: Nutrients. 2025 Nov 27;17(23):3720. doi: 10.3390/nu17233720 (PMC12694358; doi:10.3390/nu17233720)

A. Period 1/Period 2 Normalization Metabolomics

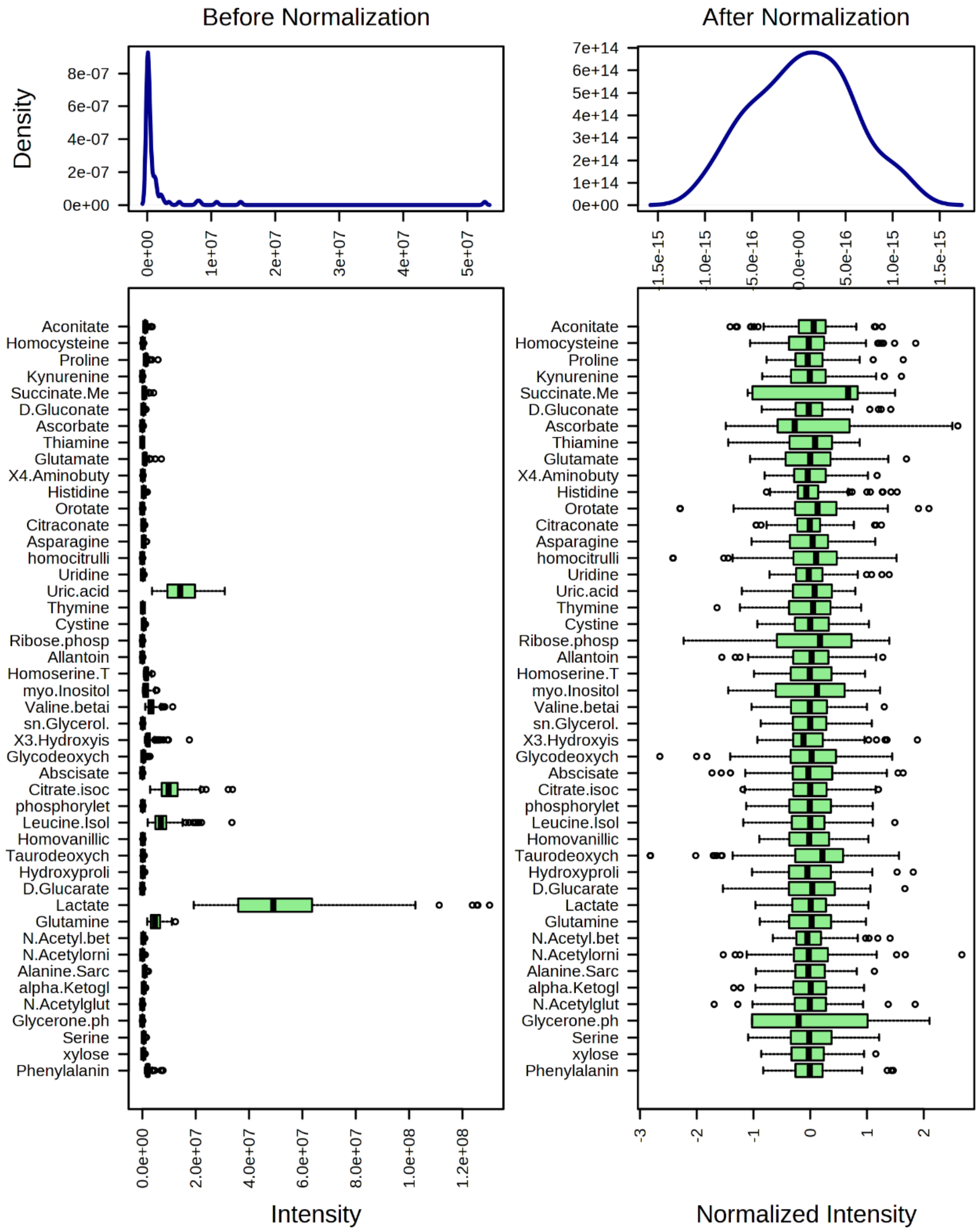

B. Blueberry First BL/Mid Normalization

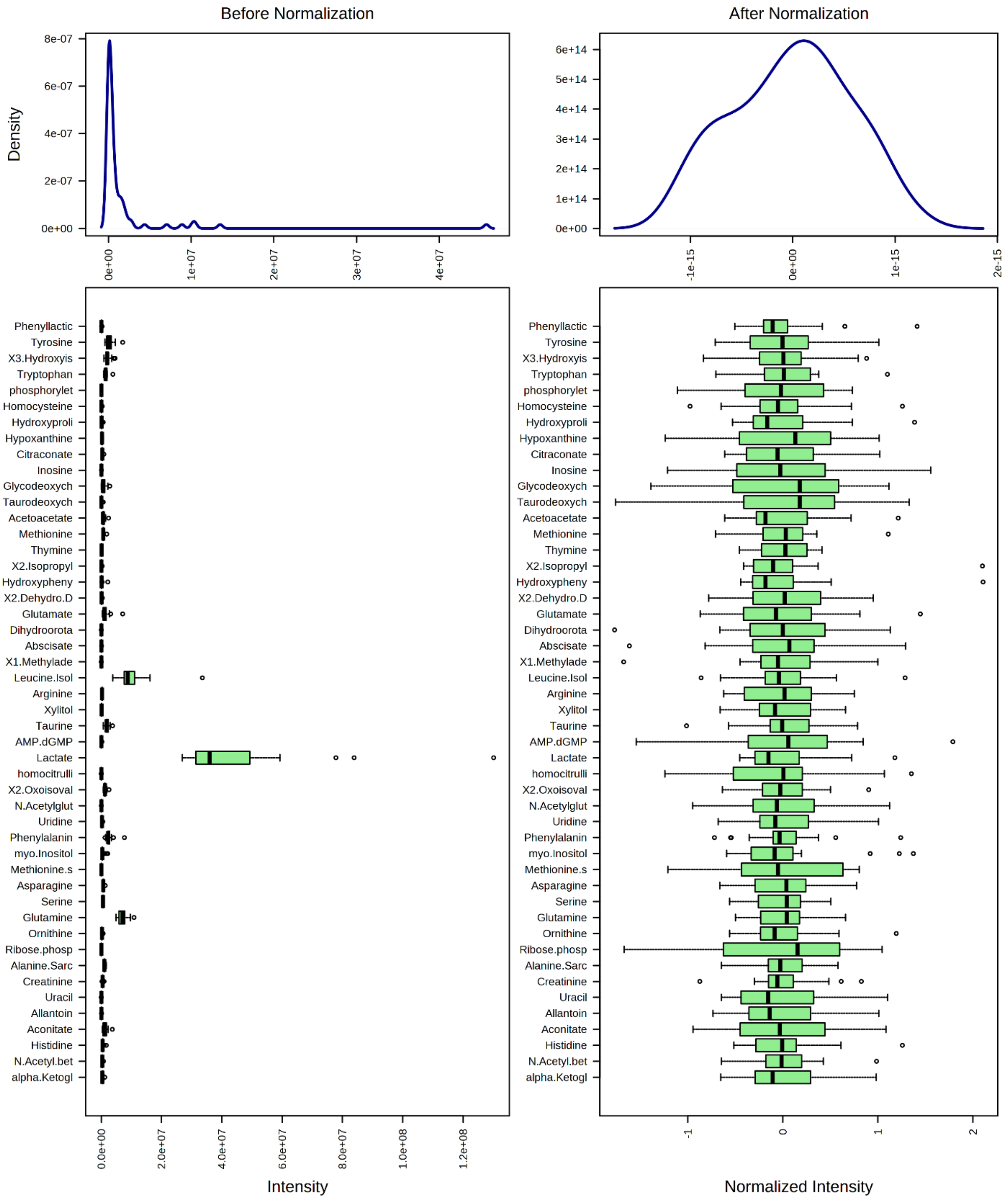

C. Blueberry First BL/Post Normalization

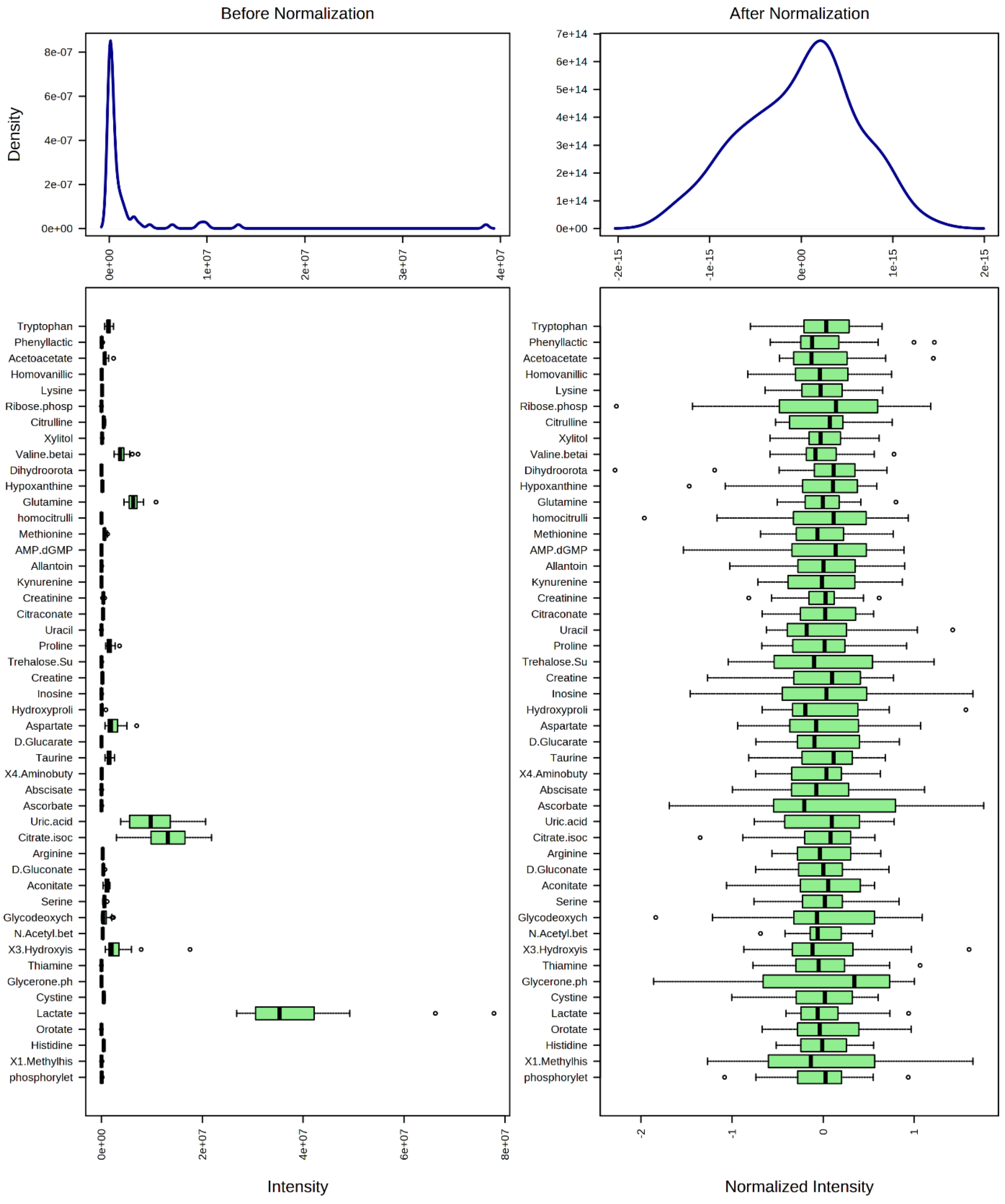

D. Placebo First BL/Mid Normalization

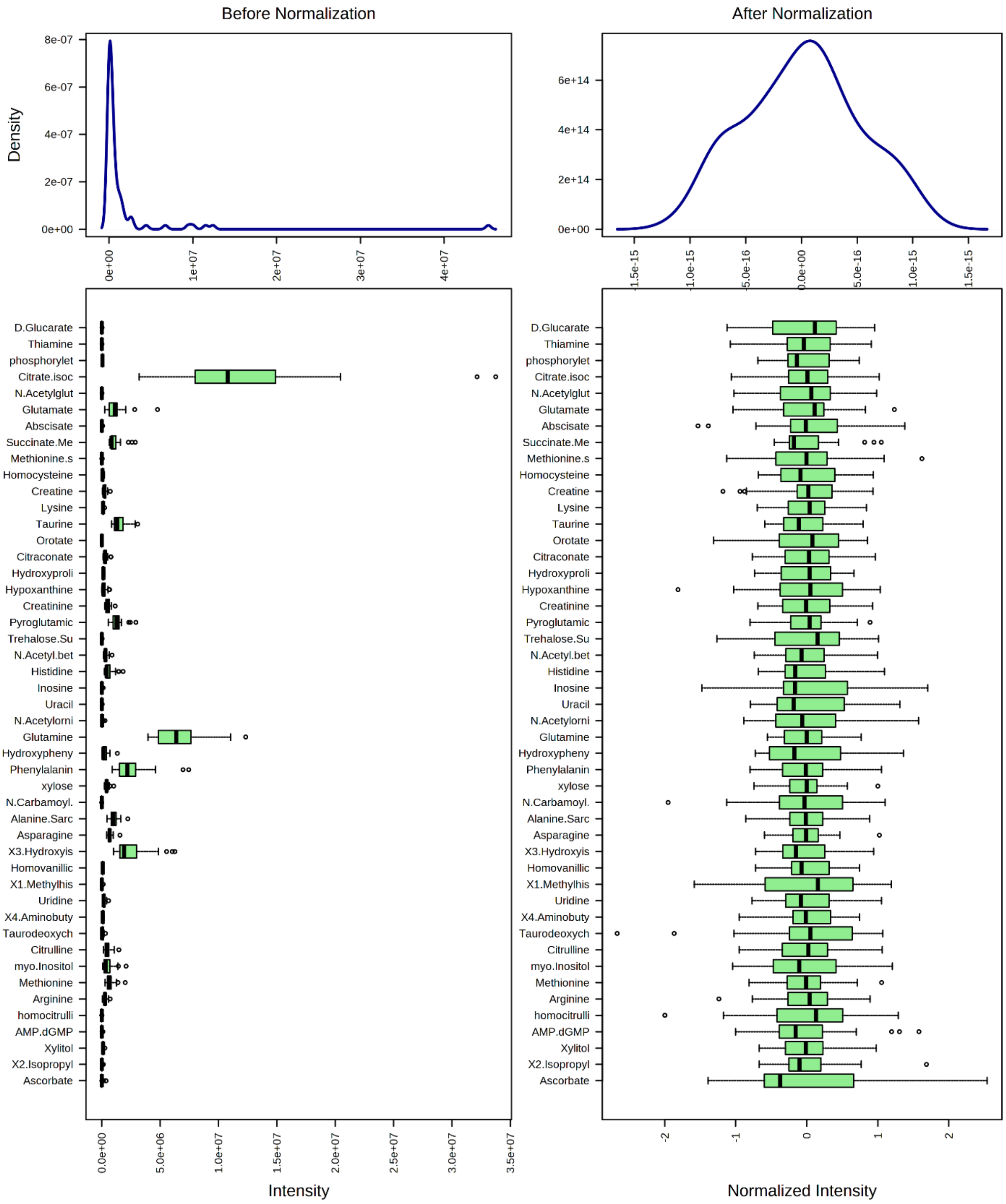

E. Placebo First BL/Post Normalization

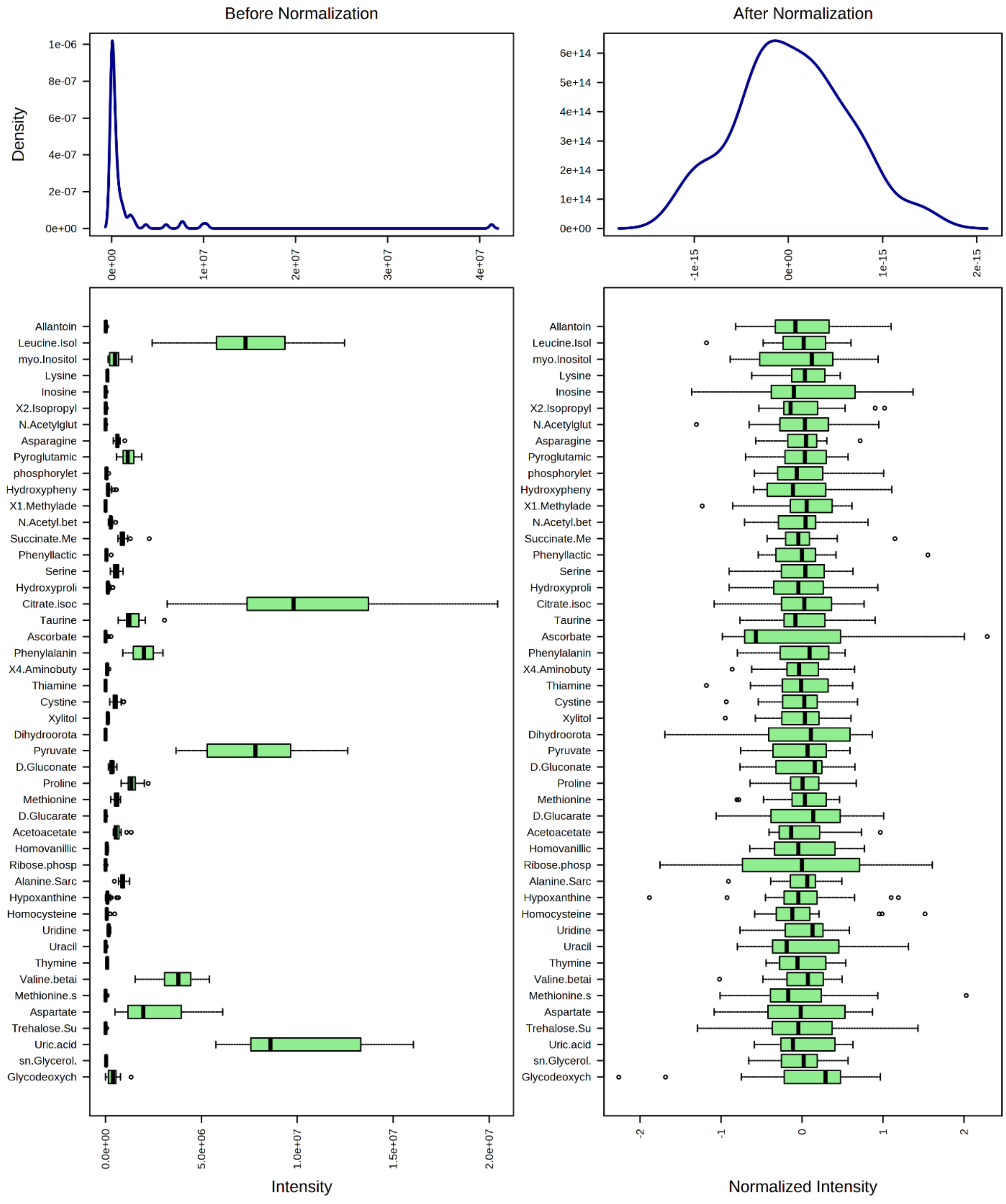

Supplement: Supplementary file 1 [file nutrients-17-03720-s001.zip › SupplementaryFileS11.pdf]
