# Supplementary material for: Effects of Blueberry Supplementation on Depression and Anxiety Symptoms in a Rural Louisiana Population
Source: Nutrients. 2025 Nov 27;17(23):3720. doi: 10.3390/nu17233720 (PMC12694358; doi:10.3390/nu17233720)
Supplement: Supplementary file 1 [file nutrients-17-03720-s001.zip › SupplementaryFileS12.pdf]

Metabolomics: BL to Mid, FC > 1.25 & FDR < 0.1

**Placebo**			**Blueberry**		
**Metabolite**	**FC**	**FDR**	**Metabolite**	**FC**	**FDR**
Histidine	0.50496	0.047005	Glutamate	0.51731	0.010599
Tyrosine	0.62295	0.047005	Creatine	0.68478	0.094951
Methionine	0.64838	0.047005	Homovanillic.acid.	0.73735	0.094951
Creatine	0.73368	0.047005			
Serine	0.76188	0.047005			
Ornithine	0.7702	0.047005			
Alanine.Sarcosine	0.72538	0.070316			
Glutamine	0.76751	0.070316			
Lysine	0.77657	0.070316			
alpha.Ketoglutarate	0.66094	0.070718			
Tryptophan	0.68994	0.070718			
sn.Glycerol.3.phosphate	0.63142	0.07322			
Leucine.Isoleucine	0.65837	0.07322			
Uridine	0.68315	0.07322			
N.Acetyl.beta.alanine	0.71246	0.07322			
Pyroglutamic.acid	0.74024	0.07322			
Creatinine	0.74273	0.07322			
Asparagine	0.79817	0.07322			
D.Gluconate	0.65983	0.085369			
X2.Dehydro.D.gluconate	0.6662	0.085369			
Citrate.isocitrate	0.68379	0.085369			
Succinate.Methylmalonate	0.76538	0.085369			
Glutamate	0.61413	0.087173			
Phenylalanine	0.68255	0.094478			
Valine.betaine	0.75336	0.094478			

Metabolomics: BL to Mid FC > 1.25

Placebo		Blueberry	
**Metabolite**	**FC**	**Metabolite**	**FC**
Ribose.phosphate	0.2971	**Taurodeoxycholate**	**0.29883**
Glycodeoxycholate	0.3473	**AMP.dGMP**	**0.40874**
Taurodeoxycholate	0.4216	**Glycodeoxycholate**	**0.4149**
Ascorbate	0.4367	**Trehalose.Sucrose**	**0.44963**
Inosine	0.4853	X1.Methylhistidine	0.5019
Histidine	0.5049	**Glutamate**	**0.51731**
D.Glucarate	0.5862	Glycerone.phosphate	0.55425
Hydroxyphenylacetate	0.6021	**Ascorbate**	**1.6265**
Hypoxanthine	0.6036	**Dihydroorotate**	**0.61849**
Aconitate	0.6130	myo.Inositol	0.66887
Glutamate	0.6141	**Creatine**	**0.68478**
N.Carbamoyl.L.aspartate	0.6180	**Aconitate**	**0.68917**
Tyrosine	0.6229	**Hypoxanthine**	**0.69316**
Dihydroorotate	0.6269	Pyruvate	0.71168
sn.Glycerol.3.phosphate	0.6314	X2.Isopropylmalate	0.7246
Methionine	0.6483	phosphorylethanolamine	0.72855
Homocysteine	0.6535	**N.Acetylglutamate**	**0.73222**
Leucine.Isoleucine	0.6583	Citrulline	0.73564
Arginine	0.6594	**Homovanillic.acid..HVA.**	**0.73735**
D.Gluconate	0.6598	Lactate	0.74249
alpha.Ketoglutarate	0.6609	**alpha.Ketoglutarate**	**0.74664**
X2.Dehydro.D.gluconate	0.6662	**X1.Methyladenosine**	**0.74786**
Methionine.sulfoxide	0.6719	Taurine	0.75023
Phenylalanine	0.6825	Uracil	0.75146
Uridine	0.6831	**Citraconate**	**0.75286**
Citrate.isocitrate	0.6837	**D.Gluconate**	**0.76773**
AMP.dGMP	0.6839	**Ornithine**	**0.77002**
Tryptophan	0.6899	**Histidine**	**0.77292**
Kynurenine	0.6930	Ribose.phosphate	1.293

N.Acetylglutamate	0.7056	**Methionine.sulfoxide**	**0.77921**
N.Acetyl.beta.alanine	0.7124	**Homocysteine**	**1.2787**
Pyruvate	0.7165	**Creatinine**	**0.79262**
Citraconate	0.7209	**sn.Glycerol.3.phosphate**	**0.79373**
X1.Methyladenosine	0.7210	**Uridine**	**0.79629**
Alanine.Sarcosine	0.7253		
Trehalose.Sucrose	0.7297		
Creatine	0.7336		
Thiamine	0.7360		
Homovanillic.acid..HVA.	0.7368		
Uric.acid	0.7387		
Pyroglutamic.acid	0.7402		
Creatinine	0.7427		
Valine.betaine	0.7533		
Proline	0.7561		
Serine	0.761		
Orotate	0.7643		
Succinate.Methylmalonate	0.7653		
Glutamine	0.7675		
Cystine	0.7681		
Ornithine	0.7702		
Lysine	0.7765		
Pantothenate	0.7901		
Asparagine	0.7981		

Metabolomics: BL to Mid Paired T-tests, FDR < 0.1

Placebo			Blueberry		
**Metabolite**	**Raw *p***	**FDR**	**Metabolite**	**Raw *p***	**FDR**
Histidine	0.001525	0.047005	**Glutamate**	**0.000139**	**0.010599**
Serine	0.001539	0.047005	**Alanine.Sarcosine**	**0.002777**	**0.094951**
Tyrosine	0.002425	0.047005	**Creatine**	**0.00423**	**0.094951**
Ornithine	0.003025	0.047005	Homovanillic.acid.HVA	0.004997	0.094951
Methionine	0.003186	0.047005			
Creatine	0.003711	0.047005			
Glutamine	0.007609	0.070316			
Alanine.Sarcosine	0.007725	0.070316			
Lysine	0.008327	0.070316			
Thymine	0.010028	0.070718			
alpha.Ketoglutarate	0.010563	0.070718			
Tryptophan	0.011166	0.070718			
Pyroglutamic.acid	0.012809	0.07322			
Leucine.Isoleucine	0.015048	0.07322			
Homoserine.Threonine	0.015187	0.07322			
Creatinine	0.016897	0.07322			
Asparagine	0.017542	0.07322			
Uridine	0.017883	0.07322			
sn.Glycerol.3.phosphate	0.018438	0.07322			
N.Acetyl.beta.alanine	0.019268	0.07322			
X2.Dehydro.D.gluconate	0.025205	0.085369			
Succinate.Methylmalonate	0.025711	0.085369			
Citrate.isocitrate	0.02619	0.085369			
D.Gluconate	0.026959	0.085369			
Glutamate	0.028675	0.087173			
X3.Hydroxyisovalerate	0.032298	0.094409			
Phenylalanine	0.034675	0.094478			
Valine.betaine	0.034808	0.094478			
