# Supplementary material for: Effects of Blueberry Supplementation on Depression and Anxiety Symptoms in a Rural Louisiana Population
Source: Nutrients. 2025 Nov 27;17(23):3720. doi: 10.3390/nu17233720 (PMC12694358; doi:10.3390/nu17233720)
Supplement: Supplementary file 1 [file nutrients-17-03720-s001.zip › SupplementaryFileS13.pdf]

DEMOGRAPHIC DATA AND CONCOMITANT PRESCRIPTIONS OF THOSE STARTING THE STUDY AT WEEK 1 (N=45)

**AGES (Years)**	**STAT**
Min	19
Max	66
Average	38
**SEX**	
Female	37 (82.2%)
Male	8 (17.8%)
**CLINIC LOCATION**	
Cottonport, LA	29 (64.4%)
Marksville, LA	16 (35.6%)
**CONCURRENT PRESCRIPTIONS (#)**	
None	13 (28.9%)
One	11 (24.4%)
Two	9 (20.0%)
Three	6 (13.3%)
Four	1 (2.2%)
No data	5 (11.1%)
**PRESCRIPTION CLASS**	
Antihistamine	1 (2.2%)
Atypical antipsychotic	1 (2.2%)
Benzodiazepine	12 (26.7%)
Beta blocker	5 (11.1%)
Buspirone	2 (4.4%)
Gabapentin	1 (2.2%)
NDRI	3 (6.7%)
SARI	2 (4.4%)
Sedative	1 (2.2%)
SNRI	1 (2.2%)
Sodium valproate	2 (4.4%)
SSNRI	4 (8.9%)
SSRI	9 (20.0%)
Stimulant	5 (11.1%)
TCS	2 (4.4%)
