# Supplementary material for: Effects of Blueberry Supplementation on Depression and Anxiety Symptoms in a Rural Louisiana Population
Source: Nutrients. 2025 Nov 27;17(23):3720. doi: 10.3390/nu17233720 (PMC12694358; doi:10.3390/nu17233720)
Supplement: Supplementary file 1 [file nutrients-17-03720-s001.zip › SupplementaryFileS14.pdf]

Generalized Anxiety Disorder 7-item (GAD-7) scale

Over the last 2 weeks, how often have you been bothered by the following problems?	Not at all sure	Several days	Over half the days	Nearly every day
1. Feeling nervous, anxious, or on edge	0	1	2	3
2. Not being able to stop or control worrying	0	1	2	3
3. Worrying too much about different things	0	1	2	3
4. Trouble relaxing	0	1	2	3
5. Being so restless that it's hard to sit still	0	1	2	3
6. Becoming easily annoyed or irritable	0	1	2	3
7. Feeling afraid as if something awful might happen	0	1	2	3
Add the score for each column	+	+	+	
Total Score *(add your column scores)* =				

If you checked off any problems, how difficult have these made it for you to do your work, take care of things at home, or get along with other people?

Not difficult at all __________
Somewhat difficult _________
Very difficult _____________
Extremely difficult _________

Source: Spitzer RL, Kroenke K, Williams JBW, Lowe B. A brief measure for assessing generalized anxiety disorder. *Arch Inern Med.* 2006;166:1092-1097.

Psychiatric Research Unit
Hillerød

Major Depression Inventory (MDI)

The following questions ask about how you have been feeling over the past two weeks. Please put a tick in the box which is closest to how you have been feeling.

	How much of the time ...	All the time	Most of the time	Slightly more than half the time	Slightly less than half the time	Some of the time	At no time
1	Have you felt low in spirits or sad?	5	4	3	2	1	0
2	Have you lost interest in your daily activities?	5	4	3	2	1	0
3	Have you felt lacking in energy and strength?	5	4	3	2	1	0
4	Have you felt less self-confident?	5	4	3	2	1	0
5	Have you had a bad conscience or feelings of guilt?	5	4	3	2	1	0
6	Have you felt that life wasn't worth living?	5	4	3	2	1	0
7	Have you had difficulty in concentrating, e.g. when reading the newspaper or watching television?	5	4	3	2	1	0
8a	Have you felt very restless?	5	4	3	2	1	0
8b	Have you felt subdued or slowed down?	5	4	3	2	1	0
9	Have you had trouble sleeping at night?	5	4	3	2	1	0
10a	Have you suffered from reduced appetite?	5	4	3	2	1	0
10b	Have you suffered from increased appetite?	5	4	3	2	1	0

Total score ☐☐

GAD-7 has 89% sensitivity / 82% selectivity for GAD (Kroenke et al. 2007)

Test	Sensitivity	Specificity	Positive Likelihood Ratio
Generalized Anxiety Disorder	89%	82%	5.1
Panic Disorder	74%	81%	3.9
Social Anxiety Disorder	72%	80%	3.6
Post-Traumatic Stress Disorder	66%	81%	3.5
Any anxiety disorder	68%	88%	5.5
