# Supplementary material for: Effects of Blueberry Supplementation on Depression and Anxiety Symptoms in a Rural Louisiana Population
Source: Nutrients. 2025 Nov 27;17(23):3720. doi: 10.3390/nu17233720 (PMC12694358; doi:10.3390/nu17233720)
Supplement: Supplementary file 1 [file nutrients-17-03720-s001.zip › SupplementaryFileS15.pdf]

		Blueberry						Placebo					
		BL		Post		Pooled		BL		Post		Pooled	
	Marker	r=	**p=**	r=	**p=**	r=	**p=**	r=	**p=**	r=	**p=**	r=	**p=**
Ham-D	TNF-a	0.31	**0.16**	0.27	**0.22**	0.15	**0.34**	-0.03	**0.89**	0.017	**0.94**	-0.06	**0.69**
	IL-10	-0.057	**0.8**	-0.046	**0.84**	-0.083	**0.59**	0.02	**0.93**	-0.0052	**0.98**	0.0018	**0.99**
	IL-6	0.075	**0.74**	0.51	**0.015**	0.18	**0.23**	0.29	**0.16**	0.04	**0.86**	0.12	**0.43**
	IL-1b	0.058	**0.8**	0.55	**0.0084**	0.35	**0.02**	0.23	**0.28**	-0.21	**0.35**	0.069	**0.65**
	IFN-y	0.32	**0.15**	0.34	**0.13**	0.23	**0.13**	0.14	**0.5**	-0.46	**0.03**	-0.076	**0.61**
GAD7	TNF-a	0.15	**0.49**	-0.16	**0.46**	-0.00076	**1**	-0.033	**0.88**	-0.16	**0.46**	-0.095	**0.44**
	IL-10	0.033	**0.88**	-0.22	**0.31**	-0.11	**0.38**	-0.25	**0.25**	-0.061	**0.78**	-0.14	**0.26**
	IL-6	0.034	**0.88**	0.1	**0.63**	0.097	**0.41**	0.077	**0.72**	-0.14	**0.45**	-0.071	**0.56**
	IL-1b	-0.22	**0.29**	-0.01	**0.96**	0.012	**0.92**	-0.03	**0.89**	-0.26	**0.22**	-0.13	**0.26**
	IFN-y	0.14	**0.51**	-0.044	**0.84**	0.028	**0.81**	0.12	**0.59**	-0.18	**0.39**	-0.02	**0.86**
MDI	TNF-a	0.24	**0.25**	0.13	**0.52**	0.099	**0.41**	0.044	**0.84**	-0.052	**0.81**	-0.094	**0.44**
	IL-10	0.056	**0.8**	0.02	**0.92**	-0.039	**0.75**	-0.099	**0.64**	-0.1	**0.63**	-0.086	**0.49**
	IL-6	0.084	**0.7**	0.35	**0.091**	0.1	**0.39**	0.19	**0.36**	-0.62	**0.78**	0.099	**0.41**
	IL-1b	0.17	**0.42**	0.35	**0.09**	0.19	**0.11**	0.36	**0.077**	-0.26	**0.22**	0.053	**0.66**
	IFN-y	0.26	**0.22**	0.19	**0.36**	0.076	**0.52**	0.18	**0.39**	-0.29	**0.18**	-0.13	**0.28**
