# Supplementary material for: Effects of Blueberry Supplementation on Depression and Anxiety Symptoms in a Rural Louisiana Population
Source: Nutrients. 2025 Nov 27;17(23):3720. doi: 10.3390/nu17233720 (PMC12694358; doi:10.3390/nu17233720)
Supplement: Supplementary file 1 [file nutrients-17-03720-s001.zip › SupplementaryFileS3.pdf]

Informed Consent for Clinical Trial

Dear Participant,

Please read this before you decide to participate in this survey study. **This document is to help you know your rights as a participant, what you will have to do, risks, and why we are doing this study**.

If you have any questions, concerns, or need to cancel participation at ANY time during this process or if you need someone to read or explain this form to you please contact the investigators:

Who are we? We are researchers from Louisiana State University working with Alec Jeansonne at Louisiana Healthcare Practitioners, LLC. Our names are Dr. Joseph Francis, Parker Kelley (PhD student), Katy Venable (PhD student), and Samia O'brien (PhD student).**The main contacts for this study are:**

Katy Venable → phone: (225) 290-2992; email: blueberrystudylsu@gmail.com
Parker Kelley → phone: (225) 303-8514; email: blueberrystudylsu@gmail.com
Dr. Joseph Francis → phone: (225) 578-9752; email: blueberrystudylsu@gmail.com

Hours: Anytime→ ****If we do not answer, please leave a message with your name and phone number--you will get a response within 24 hours.****

Title: Effect of whole blueberry powder consumption on depression: A randomized, double-blind, placebo-controlled study.

Purpose: We are conducting a clinical trial to test how freeze-dried blueberry powder effects symptoms and health markers of depression.

Location: This will depend on your clinic; the participating locations are:

Mansura Family Clinic: 6615 Saint Phillip Street, Mansura, LA 71350
Cottonport Family Clinic: 1007 Sycamore St, Suite B, Cottonport, LA 71327
Elizabeth Family Clinic: 4855 HWY 10 W, Suite C, LA 70638
Simmesport Family Clinic: 417 N Martin Luther King Dr, Simmesport, LA 71369
Marksville Family Clinic: 457 W Waddil St, Suite A, Marksville, LA 71351

Benefits: The information from this study might help to develop safer and better treatments for people with depression. It might help you feel better and decrease your inflammation, but this is not a promise—that is why we are doing this research.

Risks: Blueberries themselves pose no known physical risks except for those with certain conditions or on certain medications, but we have strict guidelines to protect you from these possibilities. You may have to be excluded for this reason. You will have to think and talk about your depression which might bring up uncomfortable feelings. If you do not want to do this, you can choose not to participate. With all clinical trials, there is a risk that your information might be exposed, but we are conducting the studies in your clinics which already follow strict rules to protect your privacy. We will also keep all documents with your information locked in a box, and we will keep all digital data safe on a password-protected, encrypted hard drive. Any analysis will be performed on data that is no longer connected to your personal information. No information about the study will be shared with your doctors and samples will only be used for the purposes in the study—to test inflammation and changes with your health.
Although this is a low risk study, there is a rare chance that an unforeseeable risk might occur. If this happens you must seek medical attention immediately and inform us as soon as you can. Our contact information is provided above. We will provide a list of emergency resources and the nearest emergency rooms for you.

Blood draws might be uncomfortable, bruise, or hurt for a little bit. We will take about 1 tsp 6 times during the study. There will be 4-6 weeks between all blood draws, and all will be performed by staff at the clinics that have plenty of experience.

Voluntary Participation and Right to Refuse: Your participation is completely voluntary. You may choose not to participate now by not signing this document. If you choose to participate, you are free to stop at any time for any reason, with no penalty. However, if you withdraw or refuse, you will not be eligible to receive any further compensation for appointments that you did not attend or the compensation for completing the study in full. If you wish to withdraw you can let us know anytime by phone or email.

What you will be asked to do in the study: This study will last about 10 months in total. For 12 weeks you will take either blueberry or placebo powder, then for 4 weeks in the middle you will not take anything, and then again for 12 weeks you will take a powder and drink mix. This is a placebo-controlled, crossover study which means: everyone will get both the real treatment and the placebo, one during the first portion of the study, and one during the second. We will not know the order that you get your treatments until after we finish analyzing the data from the study.
During this time, you will also need to come to a total of 7 appointments (each about 4-6 weeks apart) that will take about 15 minutes to an hour and a half long. The first appointment is this one. At all other appointments we will draw blood, take urine, and stool samples, and take vitals; at 4 appointments you will complete 4 short written surveys and 1 verbal interview, so that we can track your symptoms of depression. We will supply all materials for this study including the treatment powder, the mixing liquid, and the mixing bottles. We will also give you a calendar and a more visual representation of this information to clarify.

Time required and Compensation: You will be required to come to 7 appointments over about 10 months total. You will have to be actively taking a treatment for a total of 24 weeks. All appointments will be free of charge. You will get $25 for every completed appointment and compliance with treatment and $50 for completing the study in full. If you are unable or unwilling to complete the study, please contact an investigator and we will end your HIPPA agreement and remove all personally identifiable data from our records and compensate you for your participation. If a you are not able to complete the study, you will be compensated for the appointments that you did complete.

Removal of participants from the study: If you miss an appointment with no call/no show and no attempt to reschedule, provide false or misleading medical information, or else experience any significantly negative side effects, you will be removed from the study and compensated for your time.

Confidentiality/Privacy: This study is not anonymous, but all responses are confidential. All of your information will be kept in a locked filing cabinet and on a protected hard drive, only accessible to the researchers.

Results: You may get a copy of the finalized results and any reports once the data is analyzed completely. If you would like this information, please contact Katy Venable [phone-(225)290-2992; email-blueberrystudylsu@gmail.com].

Inclusion criteria:

- 60 patients with a stable diagnosis of Major Depressive Disorder (>1 year prior to enrollment)
- Males and females 21-65 years of age
- Subjects with sleep disruptions
- Subjects currently prescribed to a non-antipsychotic mono-pharmacotherapies

    - Ex: SSRI, SNRI, MAOI, Xanax, Valproic acid, Adderall (dextroamphetamine/racemic amphetamine salts or comparable)
- English speaking subjects only. (All evaluations are in English)
- Subjects with the following, inflammatory disorders that exhibit low to moderate symptoms:
    - Hypertension (mild=140/80-160/90; moderate= 160/90-180/100)
    - Asthma (requiring 2 or fewer inhalations of rescue inhaler per day)
    - Gastroesophageal reflux disease
    - Irritable bowel syndrome (controlled, <3 bowel movements a day)
    - Arthritis (controlled)
    - Chronic stomach ulcers (controlled)
    - Obesity BMI <40
    - Chronic pain
    - Fibromyalgia
    - Chronic Fatigue Syndrome
    - Type I or Type II diabetes (controlled)
- Subjects that are compliant with current treatment regimens and clinic appointments.
- Subjects taking intermittent or infrequent doses of acetaminophen or NSAIDs.
- Subjects who currently smoke or have history of smoking.

Exclusion criteria:

- Subjects with current diagnosis or history of the following conditions; or subjects currently on medication for any of the following conditions:
    - Severe Cardiovascular disease; Heart attack/pace maker
    - Cancer
    - Autoimmunity Disorders
    - **Crohn's Disease or Ulcerative Colitis**
    - **Alzheimer's Disease**
    - **Parkinson's Disease**
    - Multiple Sclerosis
    - Uncontrolled Diabetes: Type I or II
    - Severe irritable bowel disease (>3 stools per day)
    - Hypertension (severe >180/100)
    - Hypotension (<100/60)
    - Epilepsy
    - Autism Spectrum Disorder
    - Schizophrenia
    - Psychosis/Psychotic Symptoms
    - Uncontrolled Hypo/Hyper-thyroidism
- Women who are pregnant, nursing, lactating, or planning to become pregnant within timeline of study
- Subjects who are blind or deaf
- Subjects who are allergic to blueberries or other similar foods or drinks (ex wine), or subjects who are allergic to red or blue food dye agents.
- Subjects who do not like the taste of blueberries
- Subjects who do not want to disclose information related to their Major Depressive Disorder
- Subjects who do not want to be subjected to blood draws
- Subjects who consume >4 cups of blueberries per week or other foods/drinks with significant polyphenol content

- Subjects supplementing with elderberry syrup >4 times per week
- Subjects who have a planned surgery during the timeline of the study
- Subjects prescribed to antipsychotics.
- Subjects using acetaminophen or NSAIDS (drugs targeting pro-inflammatory paths) chronically or exceeding recommended daily doses
- Subjects chronically on Decadron, Dexamethasone, or Prednisone; or other oral steroids.
- Subjects on any augmenting agents (the following is not an inclusive list):
    - Abilify (aripiprazole), Risperdal (risperidone), Zyprexa (olanzapine), Seroquel (quetiapine), Cloazril (clozapine), Symbyax (olanzapine/fluoxetine), Geodon (ziprasidone)
- Subjects supplementing with devil's claw, fenugreek, guar gum, Panax ginseng, and Siberian ginseng
- Subjects who have a history of suicidal ideation or suicide attempt
- Subjects with a history or record of physical violence toward self or others
- Subjects who will jeopardize their job if they miss work for appointments
- Subjects with a history of addiction, except cigarettes

Thank you for reading this! If you do not wish to participate, please do not sign this.

The study has been discussed with me and all my questions have been answered. I may direct additional questions **regarding study specifics to the investigators. If I have questions about subjects' rights or other concerns, I can contact** Dennis Landin, Chairman, LSU Institutional Review Board, (225)578-8692, irb@lsu.edu, www.lsu.edu/irb. I agree to **Participate in the study described above and acknowledge the researchers' obligation to provide me with a copy of this** consent form if signed by me.

Signature of Participant: ______________________ **Date:** ______________

The study subject has indicated to me that he/she is unable to read. I certify that I have read this consent form to the subject and explained that by completing the signature line above, the subject has agreed to participate.

Signature of Reader: ______________________ **Date:** ______________
