# Supplementary material for: Effects of Blueberry Supplementation on Depression and Anxiety Symptoms in a Rural Louisiana Population
Source: Nutrients. 2025 Nov 27;17(23):3720. doi: 10.3390/nu17233720 (PMC12694358; doi:10.3390/nu17233720)
Supplement: Supplementary file 1 [file nutrients-17-03720-s001.zip › SupplementaryFileS4.pdf]

Can BLUEBERRIES help manage your symptoms of depression?

YOU could help us find out!

-AND-

✓ GET PAID up to $200

✓ DRINK FREE blueberry drink everyday

✓ GET FREE health screenings

BLUEBERRIES ARE SUPERFOODS!

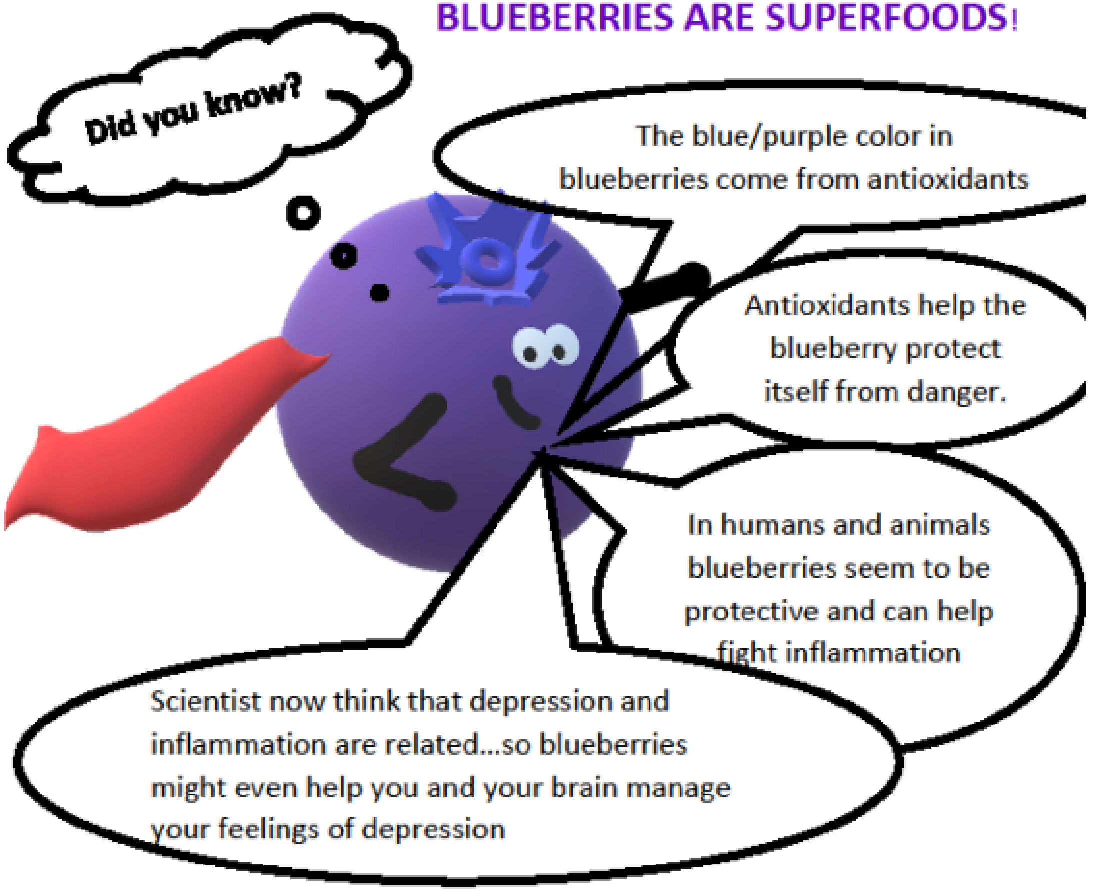

What will the study be like?

- We will provide FREE convenient, single-serve blueberry drinks—you just need to drink one a day.
- You do NOT have to make any other changes to your diet, medication, or lifestyle
- You will be required to come to 6 FREE appointments at your clinic: Each 30 days apart.
- At 4 of the 6 appointments, we will track changes in your symptoms with interviews with a health care professional
  - ***this will require you to answer specific questions about your symptoms, including your trauma; if this makes you uncomfortable, please indicate "NO" on this survey***
- We will also collect blood, urine, and saliva samples at the appointments to track changes to your inflammation.
- There are no physical risks. However, we will have to draw blood 6 times. Each will be 30 days apart.

Would you be interested in participating in this research study?

☐ **Yes,** I am interested in learning more about this study **and/or** participating in the future.

*****If you checked this box, please read and fill out the pages that follow*****

****Checking yes does not automatically enroll or obligate you to participate****

☐ **No,** I am not interested in learning more about this study or participating in the future.

Please indicate a reason you are not interested or cannot participate

____

____

____

*****If you checked no, you SHOULD skip the next pages, BUT PLEASE RETURN THIS PACKET This information will be helpful in designing future projects*****
