# Supplementary material for: Effects of Blueberry Supplementation on Depression and Anxiety Symptoms in a Rural Louisiana Population
Source: Nutrients. 2025 Nov 27;17(23):3720. doi: 10.3390/nu17233720 (PMC12694358; doi:10.3390/nu17233720)
Supplement: Supplementary file 1 [file nutrients-17-03720-s001.zip › SupplementaryFileS5.pdf]

Nutritional Intake and Lifestyle Survey

This survey is to help us get a general idea of your diet. Your honesty will help us do better research!

Circle your response. If you do not know or cannot remember exactly, it is okay. Your best guesses are fine.

QUESTIONS ABOUT GENERAL DIET:

1. About how often do you eat a home-cooked meal?

Never   Less than 1 time per month   1-3 times per month   1-2 times a week   Every day or more than 3 days a week

2. About how often do you eat fast food? (ex: Burger King, McDonalds, Wendy's, Popeyes, Church's, Subway, Sonic)

Never   Less than 1 time per month   1-3 times per month   1-2 times a week   Every day or more than 3 days a week

3. About how often do you eat or pick up food from sit-down restaurants?

Never   Less than 1 time per month   1-3 times per month   1-2 times a week   Every day or more than 3 days a week

4. About how often do you eat red meats (examples: cow beef, pork, lamb, goat, mutton)?

Never   Less than 1 time per month   1-3 times per month   1-2 times a week   Every day or more than 3 days a week

5. How often do you eat meat that you or someone you know hunted (examples: deer, hog)?

Never   Less than 1 time per month   1-3 times per month   1-2 times a week   Every day or more than 3 days a week

6. About how often do you eat chicken, duck, or turkey?

Never   Less than 1 time per month   1-3 times per month   1-2 times a week   Every day or more than 3 days a week

7. If you eat chicken, duck, or turkey, about how often is it fried?

Never   Less than 1 time per month   1-3 times per month   1-2 times a week   Every day or more than 3 days a week

8. About how often do you eat fish or seafood?

Never   Less than 1 time per month   1-3 times per month   1-2 times a week   Every day or more than 3 days a week

9. About how often do you eat canned, lunch meats or processed meats (ham, turkey, bacon, bologna, sausage, spam)?

Never   Less than 1 time per month   1-3 times per month   1-2 times a week   Every day or more than 3 days a week

10. About how often do you have fried food?

Never Less than 1 time per month 1-3 times per month 1-2 times a week Every day or more than 3 days a week

11. About how often do you eat raw or cooked vegetables?

Never Less than 1 time per month 1-3 times per month 1-2 times a week Every day or more than 3 days a week

12. About how often do you eat beans?

Never Less than 1 time per month 1-3 times per month 1-2 times a week Every day or more than 3 days a week

13. About how often do you eat rice?

Never Less than 1 time per month 1-3 times per month 1-2 times a week Every day or more than 3 days a week

14. About how often do you eat fruit?

Never Less than 1 time per month 1-3 times per month 1-2 times a week Every day or more than 3 days a week

15. About how often do you drink soda/soft drinks, cool aid, sweet tea, Gatorade or Powerade (not sugar free)?

Never Less than 1 time per month 1-3 times per month 1-2 times a week Every day or more than 3 days a week

16. About how often do you drink fruit juices (orange juice, grapefruit juice, pineapple juice)?

Never Less than 1 time per month 1-3 times per month 1-2 times a week Every day or more than 3 days a week

17. About how often do you drink vegetable juices?

Never Less than 1 time per month 1-3 times per month 1-2 times a week Every day or more than 3 days a week

18. About how often do you eat or drink dairy (cow or goat milk, yogurt, or cheese)?

Never Less than 1 time per month 1-3 times per month 1-2 times a week Every day or more than 3 days a week

19. About how often do you eat sweets or desserts?

Never Less than 1 time per month 1-3 times per month 1-2 times a week Every day or more than 3 days a week

QUESTIONS ABOUT OTHER FOODS AND DRINKS WITH ANTIOXIDANTS LIKE BLUEBERRIES:

20. About how often do you eat fresh, frozen, or cooked blueberries when they are in season?

Never Less than 1 time per month 1-3 times per month 1-2 times a week Every day or more than 3 days a week

21. About how often do you eat fresh, frozen, or cooked blueberries when they are not in season?

Never Less than 1 time per month 1-3 times per month 1-2 times a week Every day or more than 3 days a week

22. About how often do you eat fresh, frozen, or cooked strawberries when they are in season?

Never Less than 1 time per month 1-3 times per month 1-2 times a week Every day or more than 3 days a week

23. About how often do you eat fresh, frozen, or cooked strawberries when they are not in season?

Never Less than 1 time per month 1-3 times per month 1-2 times a week Every day or more than 3 days a week

24. About how often do you eat fresh, frozen, or cooked blackberries?

Never Less than 1 time per month 1-3 times per month 1-2 times a week Every day or more than 3 days a week

25. About how often do you eat fresh, frozen, or cooked dewberries?

Never Less than 1 time per month 1-3 times per month 1-2 times a week Every day or more than 3 days a week

26. About how often do you eat fresh, frozen, or cooked raspberries?

Never Less than 1 time per month 1-3 times per month 1-2 times a week Every day or more than 3 days a week

27. About how often do you eat fresh, frozen, or cooked huckleberries?

Never Less than 1 time per month 1-3 times per month 1-2 times a week Every day or more than 3 days a week

28. About how often do you eat fresh, frozen, or cooked lingonberries?

Never Less than 1 time per month 1-3 times per month 1-2 times a week Every day or more than 3 days a week

29. About how often do you drink red wine or red grape juice?

Never Less than 1 time per month 1-3 times per month 1-2 times a week Every day or more than 3 days a week

30. About how often do you eat fresh or dried herbs (ex: basil, oregano, thyme, parsley, cilantro)?

Never Less than 1 time per month 1-3 times per month 1-2 times a week Every day or more than 3 days a week

31. About how often do you take or drink elderberry syrup?

Never Less than 1 time per month 1-3 times per month 1-2 times a week Every day or more than 3 days a week

QUESTIONS ABOUT LIFESTYLE:

32. About how often do you exercise in general?

Never Less than 1 time per month 1-3 times per month 1-2 times a week Every day or more than 3 days a week

33. If you exercise, how would you describe it?

I don't exercise Intense Medium **Light**

34. About how often do you resistance or strength training (ex-lifting free weights, weight machines, push-ups/pull ups, power yoga)?

Never Less than 1 time per month 1-3 times per month 1-2 times a week Every day or more than 3 days a week

35. About how often do you do aerobic exercise (ex- running, biking, swimming, jump roping)?

Never Less than 1 time per month 1-3 times per month 1-2 times a week Every day or more than 3 days a week
