# Supplementary material for: Effects of Blueberry Supplementation on Depression and Anxiety Symptoms in a Rural Louisiana Population
Source: Nutrients. 2025 Nov 27;17(23):3720. doi: 10.3390/nu17233720 (PMC12694358; doi:10.3390/nu17233720)
Supplement: Supplementary file 1 [file nutrients-17-03720-s001.zip › SupplementaryFileS6.pdf]

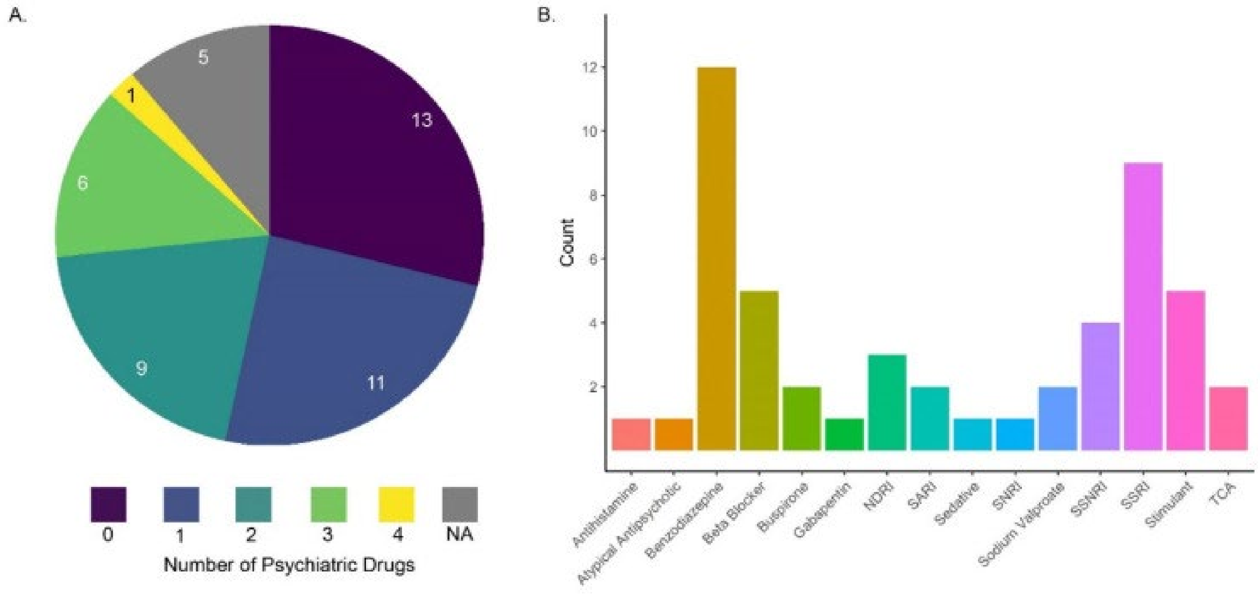

Supplementary Figure 4: Figure repurposed from **Figure 1** of our prior publication: Venable, K. E., Lee, C. C., & Francis, J. (2024). Addressing Mental Health in Rural Settings: A Narrative Review of Blueberry Supplementation as a Natural Intervention. *Nutrients*, *16*(20), 3539.
