# Supplementary material for: Effects of Blueberry Supplementation on Depression and Anxiety Symptoms in a Rural Louisiana Population
Source: Nutrients. 2025 Nov 27;17(23):3720. doi: 10.3390/nu17233720 (PMC12694358; doi:10.3390/nu17233720)
Supplement: Supplementary file 1 [file nutrients-17-03720-s001.zip › SupplementaryFileS7.pdf]

PRODUCT SPECIFICATON
USHBC Blueberry Placebo Formula #114
2016 Pack

PRODUCT CHARACTERISTICS

**Description:**	Purple colored, free-flowing powder with blueberry aromatics
**Organic:**	No
**Kosher:**	No

**PRODUCT PROFILE**	**METHOD**	**SPECIFICATION**
Moisture	CM4012	REPORT ONLY
Color	Visual	Characteristic

MICROBIOLOGICAL PROFILE	***METHOD***	***SPECIFICATION***
Total Aerobic Plate Count	FDA BAM 8th Ed.	REPORT ONLY
Coliform MPN Method	FDA BAM 8th Ed.	REPORT ONLY
E. coli – MPN Method	AOAC RI 050501	REPORT ONLY
Fecal Coliforms – MPN Method	FDA BAM 8th Ed.	REPORT ONLY
Salmonella	AOAC 2009.03	REPORT ONLY
Staphylococcus aureus	AOAC 2003.07<br>AOAC 2003.08	REPORT ONLY
Yeast	FDA BAM 7th Ed.	REPORT ONLY
Mold	FDA BAM 7th Ed.	REPORT ONLY

INGREDIENT STATEMENT

Dextrose, maltodextrin, fructose, citric acid, malic acid, natural and artificial flavor, xanthan gum, silicon dioxide, FD&C Red 40 and Blue 2 lakes.

PACKAGING AND STORAGE

Pack size: 25 lb. plastic bag in a cardboard box.
Packaging: Bulk: 3 mil poly bags with cable tie in metal drums.
Storage: Cool, Dry.
Expected shelf-life: Formal shelf-life has not been conducted on this product, however, expected shelf-life is 18 – 24 months from date of manufacture in unopened containers, based on experience with products containing similar ingredients.

Issue Date: 10/03/16

COVANCE.
SOLUTIONS MADE REAL®

365 North Canyons Parkway, Suite 201, Livermore CA 94551 Tech Center: 2441 Constitution Drive, Livermore CA 94551
925.828.1440 925.243.0117 fax

U.S. HIGHBUSH BLUEBERRY COUNCIL
1847 Iron Point Road, Suite 100 Folsom, California 95630
Phone (916) 983-0111 Fax (916) 983-9022 Web Site: www.blueberry.org

Freeze Dried Blueberry Powder – 71717
Tifblue/Rubel 50/50 Blend

PRODUCT CHARACTERISTICS

Botanical Name	*Vaccinium virgatum (ashei)/Vaccinium corymbosum*
Plant Part	Berry
Mesh Size	20
Fresh Weight Replacement	1 to 6 (freeze dried – fresh)
Packing	Packed in cans with O_2 absorber
ORAC (µmole TE/g)	515
Phenolics (mg/g)	31
Anthocyanins (mg/g)	10.6

NUTRITIONAL INFORMATION (per 100 grams)

Calories (kcal)	394
Protein (g)	2.93
Carbohydrates (g)	91.5
Fat (g)	1.84
Saturated Fat (g)	0.23
Trans Fatty Acids (g)	0
Total Sugars (g)	70.4
Fructose (g)	35.9
Glucose (g)	34.1
Sucrose (g)	<0.1
Maltose (g)	0.435
Lactose (g)	<0.1
Dietary Fiber (g)	22.6
Insoluble Fiber (g)	17.7
Soluble Fiber (g)	4.9
Cholesterol (mg)	0
Total Beta Carotene (ug RAE)	6.04
Vitamin C (mg)	17.0
Calcium (mg)	42.6
Iron (mg)	<1.0
Potassium (mg)	478
Sodium (mg)	<3.0

*Keep any unused powder in a tightly sealed container, away from light and air.
