# Supplementary material for: Effects of Blueberry Supplementation on Depression and Anxiety Symptoms in a Rural Louisiana Population
Source: Nutrients. 2025 Nov 27;17(23):3720. doi: 10.3390/nu17233720 (PMC12694358; doi:10.3390/nu17233720)
Supplement: Supplementary file 1 [file nutrients-17-03720-s001.zip › SupplementaryFileS8.pdf]

HDRS MEMRM Formula, Output, and Evaluation

Formula: HDRS_num ~ tx + arm + tx : appt_cat + bb_first + appt_cat + (1 | de_id)

REML criterion at convergene: 728.8521

Number of Observations: 117

Groups: de_id, 45

Random Effects

Groups	Name	Variance	Std.Dev.
de_id	(Intercept)	27.17	5.213
Residual		20.35	4.512

Fixed Effects

	Estimate	Std. Error	Df	t value	Pr(>|t|)
(Intercept)	22.3307	1.568	69.8573	14.242	< 2e-16 ***
txplacebo	-2.958	1.1893	78.8283	-2.487	0.0150 *
arm2	-3.7301	0.9048	74.5153	-4.122	9.63e-05 ***
bb_first1	-0.6979	1.867	41.2483	-0.374	0.7105
appt_catpost	-9.1643	1.3048	76.5196	-7.024	7.70e-10 ***
txplacebo:appt_catpost	3.6863	1.7911	74.4646	2.058	0.0431 *

Confidence Intervals

	2.50%	97.50%
.sig01	3.720047	6.8211311
.sigma	3.756577	5.2249223
(Intercept)	19.3076	25.382692
txplacebo	-5.249446	-0.654256
arm2	-5.489181	-1.987549
bb_first1	-4.35856	2.9366266
appt_catpost	-11.68534	-6.645207
txplacebo:appt_catpost	0.209713	7.1370563

GAD-7 MERM Formula, Output, and Evaluation

Formula: GAD-7_num ~ tx + arm + tx : appt_cat + bb_first + appt_cat + (1 | de_id)

REML criterion at convergence: 1088.878

Number of obs: 186, groups: de_id, 45

Groups: de_id, 45

Random effects

Groups	Name	Variance	Std.Dev.
de_id	(Intercept)	16.58	4.072
Residual		15.55	3.944

Fixed effects

	Estimate	Std. Error	df	t value	Pr(>|t|)
(Intercept)	14.8533	1.2052	80.3263	12.324	< 2e-16 ***
txplacebo	-2.352	0.9832	150.1047	-2.392	0.017986 *
arm2	-2.118	0.6161	146.09	-3.438	0.000764 ***
bb_first1	-1.1477	1.408	42.9288	-0.815	0.41949
appt_catmid	-3.6776	0.9732	143.5951	-3.779	0.000230 ***
appt_catpost	-5.3579	1.0651	145.7807	-5.03	1.42e-06 ***
txplacebo:appt_catmid	2.3363	1.4058	142.8232	1.662	0.098710 .
txplacebo:appt_catpost	3.1336	1.4762	144.6842	2.123	0.035477 *

Confidence Intervals

	2.50%	97.50%
.sig01	3.0324074	5.2158437
.sigma	3.4532686	4.3567403
(Intercept)	12.5152924	17.182565
txplacebo	-4.2542973	-0.4563242
arm2	-3.3085073	-0.9290167
bb_first1	-3.89098	1.6090538
appt_catmid	-5.5719947	-1.8021369
appt_catpost	-7.4264097	-3.3053762
txplacebo:appt_catmid	-0.3738379	5.0603749
txplacebo:appt_catpost	0.288617	5.997829

MDI MEMRM Formula, Output, and Evaluation

Formula: mdi_num ~ tx + arm + tx : appt_cat + bb_first + appt_cat + (1 | de_id)

REML criterion at convergence: 1270.898

Number of obs: 185

Groups: de_id, 45

Random effects

Groups	Name	Variance	Std.Dev.
de_id	(Intercept)	75.13	8.668
Residual		40.33	6.351

Fixed effects

	Estimate	Std. Error	df	t value	Pr(>|t|)
(Intercept)	26.481	2.316	70.346	11.436	< 2e-16 ***
txplacebo	-1.82	1.601	145.936	-1.137	0.257496
arm2	-4.071	1.011	142.769	-4.028	9.09e-05 ***
bb_first1	-2.493	2.847	44.345	-0.876	0.385993
appt_catmid	-6.114	1.584	141.045	-3.86	0.000172 ***
appt_catpost	-6.716	1.711	142.294	-3.926	0.000134 ***
txplacebo:appt_catmid	2.31	2.295	140.305	1.006	0.316011
txplacebo:appt_catpost	0.564	2.365	141.22	0.238	0.811876

Confidence Intervals

	2.50%	97.50%
.sig01	6.681685	10.88172
.sigma	5.556403	7.016633
(Intercept)	21.99081	30.97364
txplacebo	-4.91375	1.268403
arm2	-6.03335	-2.12369
bb_first1	-8.05821	3.064729
appt_catmid	-9.18187	-3.06096
appt_catpost	-10.0383	-3.41996
txplacebo:appt_catmid	-2.12018	6.74447
txplacebo:appt_catpost	-3.99507	5.147035

IL-6 MEMRM Formula, Output, and Evaluation

Formula: log10(il6_2) ~ tx + arm + tx : appt_cat + bb_first + appt_cat + (1 | de_id)

REML criterion at convergence: -6.8012

Number of obs: 146, **Groups:** de_id, 2

Random effects

Groups	Name	Variance	Std.Dev.
**de_id**	(Intercept)	0.05756	0.2399
**Residual**		0.02972	0.1724

Fixed effects

	Estimate	Std. Error	df	t value	Pr(>|t|)
**(Intercept)**	3.538706	0.077583	37.79366	45.612	<2e-16 ***
**txplacebo**	-0.06436	0.048684	113.6086	-1.322	0.1888
**arm2**	-0.00592	0.029249	114.6134	-0.202	0.8401
**bb_first1**	-0.01631	0.096907	25.07084	-0.168	0.8677
**appt_catmid**	-0.03845	0.049018	113.3152	-0.784	0.4344
**appt_catpost**	-0.08719	0.049	113.321	-1.779	0.0779 .
**txplacebo:appt_catmid**	0.047137	0.069609	113.3006	0.677	0.4997
**txplacebo:appt_catpost**	0.062918	0.070367	113.5987	0.894	0.3731

Confidence Intervals

	2.50%	97.50%
**.sig01**	0.17441	0.315658
**.sigma**	0.148742	0.191842
**(Intercept)**	3.388549	3.688977
**txplacebo**	-0.15803	0.029454
**arm2**	-0.06216	0.050467
**bb_first1**	-0.2058	0.17295
**appt_catmid**	-0.13276	0.056024
**appt_catpost**	-0.18135	0.007402
**txplacebo:appt_catmid**	-0.08699	0.181082
**txplacebo:appt_catpost**	-0.07305	0.198061

IL-1β MEMRM Formula, Output, and Evaluation

Formula: log10(il1b_2) ~ tx + arm + tx : appt_cat + bb_first + appt_cat + (1 | de_id)

REML criterion at convergence: 12.8008

Number of obs: 126, **Groups:** de_id, 27

Random effects

**Groups**	**Name**	**Variance**	**Std.Dev.**
**de_id**	(Intercept)	0.04323	0.2079
**Residual**		0.03595	0.1896

Fixed effects

	**Estimate**	**Std. Error**	**df**	**t value**	**Pr(>|t|)**
**(Intercept)**	2.1467873	0.074807	39.916327	28.698	<2e-16 ***
**txplacebo**	0.0619685	0.0569604	94.1869305	1.088	0.2794
**arm2**	0.0027183	0.0348809	93.3995104	0.078	0.938
**bb_first1**	-0.000788	0.0884121	21.281811	-0.009	0.993
**appt_catmid**	-0.01219	0.0586374	92.3176968	-0.208	0.8358
**appt_catpost**	-0.130743	0.0595336	92.4305909	-2.196	0.0306 *
**txplacebo:appt_catmid**	-0.053644	0.0829669	92.980787	-0.647	0.5195
**txplacebo:appt_catpost**	-0.000652	0.0844709	92.2307348	-0.008	0.9939

Confidence Intervals

	**2.50%**	**97.50%**
**.sig01**	0.13926413	0.28549455
**.sigma**	0.16061089	0.21354792
**(Intercept)**	2.00375334	2.29343444
**txplacebo**	-0.04683391	0.17268558
**arm2**	-0.06435695	0.06957604
**bb_first1**	-0.17661377	0.1704008
**appt_catmid**	-0.12507562	0.10015285
**appt_catpost**	-0.24521493	-0.016592
**txplacebo:appt_catmid**	-0.21338218	0.10527292
**txplacebo:appt_catpost**	-0.16451203	0.16075602

TNF-α MEMRM Formula, Output, and Evaluation

Formula: log10(tnfa_2) ~ tx + arm + tx : appt_cat + bb_first + appt_cat + (1 | de_id)

REML criterion at convergence: -252.1181

Number of obs: 146, **Groups:** de_id, 27

Random effects

Groups	Name	Variance	Std.Dev.
de_id	(Intercept)	0.11084	0.11084
Residual		0.004795	0.06925

Fixed effects

	Estimate	Std. Error	df	t value	Pr(>|t|)
(Intercept)	3.01	0.03	35.02	86.887	<2e-16 ***
txplacebo	0.00	0.02	113.70	0.107	0.915
arm2	0.00	0.01	114.50	-0.407	0.685
bb_first1	0.01	0.04	25.26	0.26	0.797
appt_catmid	-0.02	0.02	113.40	-1.206	0.23
appt_catpost	-0.02	0.02	113.40	-0.961	0.339
txplacebo:appt_catmid	0.00	0.03	113.40	-0.017	0.986
txplacebo:appt_catpost	-0.02	0.03	113.70	-0.583	0.561

Confidence Intervals

	2.50%	97.50%
.sig01	0.081444	0.14500774
.sigma	0.059746	0.07704192
(Intercept)	2.945204	3.07966918
txplacebo	-0.03554	0.03977831
arm2	-0.02737	0.01791293
bb_first1	-0.07507	0.09796313
appt_catmid	-0.06166	0.01417065
appt_catpost	-0.05679	0.01900727
txplacebo:appt_catmid	-0.0543	0.05337437
txplacebo:appt_catpost	-0.0709	0.03796437

IFN-γ MEMRM Formula, Output, and Evaluation

Formula: log10(ifny_2) ~ tx + arm + tx : appt_cat + bb_first + appt_cat + (1 | de_id)

REML criterion at convergence: 150.3028

Number of obs: 146, Groups: de_id, 27

Random Effects

Groups	Name	Variance	Std.Dev.
de_id	(Intercept)	0.02191	0.148
Residual		0.12783	0.3575

Fixed Effects

	Estimate	Std. Error	df	t value	Pr(>|t|)
**(Intercept)**	2.827996	0.093169	100.800	30.353	<2e-16 ***
**txplacebo**	0.038915	0.101653	115.919	0.383	0.703
**arm2**	0.049208	0.059973	119.391	0.821	0.414
**bb_first1**	-0.064754	0.082709	25.193	-0.783	0.441
**appt_catmid**	0.003724	0.102582	115.226	0.036	0.971
**appt_catpost**	-0.108295	0.101312	114.711	-1.069	0.287
**txplacebo:appt_catmid**	-0.025965	0.14488	114.817	-0.179	0.858
**txplacebo:appt_catpost**	0.023357	0.144982	115.274	0.161	0.872

Confidence Intervals

	2.50%	97.50%
**.sig01**	0.04543	0.230818
**.sigma**	0.308624	0.397786
**(Intercept)**	2.649480	3.006771
**txplacebo**	-0.156022	0.236052
**arm2**	-0.066276	0.164568
**bb_first1**	-0.22685	0.095964
**appt_catmid**	-0.194023	0.20106
**appt_catpost**	-0.303025	0.087272
**txplacebo:appt_catmid**	-0.305637	0.252498
**txplacebo:appt_catpost**	-0.25755	0.3014995

IL-10 MEMRM Formula, Output, and Evaluation

Formula: log10(il10_2) ~ tx + arm + tx : appt_cat + bb_first + appt_cat + (1 | de_id)

REML criterion at convergence: -5.2104

Number of obs: 140, **Groups:** de_id, 26

Random effects

Groups	Name	Variance	Std.Dev.
de_id	(Intercept)	0.03319	0.1822
Residual		0.03311	0.182

Fixed effects

	Estimate	Std. Error	df	t value	Pr(>|t|)
(Intercept)	2.724684	0.067903	46.30027	40.126	<2e-16 ***
txplacebo	0.005806	0.052427	109.6456	0.111	0.912
arm2	-0.0092	0.031499	111.2264	-0.292	0.771
bb_first1	0.060479	0.078262	24.73465	0.773	0.447
appt_catmid	-0.03365	0.052795	109.1873	-0.637	0.525
appt_catpost	0.034038	0.052775	109.1971	0.645	0.52
txplacebo:appt_catmid	0.022788	0.074988	109.1592	0.304	0.762
txplacebo:appt_catpost	-0.05631	0.075804	109.6619	-0.743	0.459

Confidence Intervals

	2.50%	97.50%
.sig01	6.681685	10.88172
.sigma	5.556403	7.016633
(Intercept)	21.99081	30.97364
txplacebo	-4.91375	1.268403
arm2	-6.03335	-2.12369
bb_first1	-8.05821	3.064729
appt_catmid	-9.18187	-3.06096
appt_catpost	-10.0383	-3.41996
txplacebo:appt_catmid	-2.12018	6.74447
txplacebo:appt_catpost	-3.99507	5.147035
