# Supplementary material for: Effects of Blueberry Supplementation on Depression and Anxiety Symptoms in a Rural Louisiana Population
Source: Nutrients. 2025 Nov 27;17(23):3720. doi: 10.3390/nu17233720 (PMC12694358; doi:10.3390/nu17233720)
Supplement: Supplementary file 1 [file nutrients-17-03720-s001.zip › SupplementaryFileS9.pdf]

behavior scripts

2024-01-02

R Markdown

This is an R Markdown document. Markdown is a simple formatting syntax for authoring HTML, PDF, and MS Word documents. For more details on using R Markdown see http://rmarkdown.rstudio.com.

Packages

```
library(tidyverse)

## ── Attaching core tidyverse packages ──────────────────────── tidyv
erse 2.0.0 ──
## ✔ dplyr     1.1.1     ✔ readr     2.1.4
## ✔ forcats   1.0.0     ✔ stringr   1.5.0
## ✔ ggplot2   3.4.1     ✔ tibble    3.2.1
## ✔ lubridate 1.9.2     ✔ tidyr     1.3.0
## ✔ purrr     1.0.1
## ── Conflicts ────────────────────────────────────────── tidyverse_c
onflicts() ──
## ✖ dplyr::filter() masks stats::filter()
## ✖ dplyr::lag()    masks stats::lag()
## ℹ Use the conflicted package (<http://conflicted.r-lib.org/>) to fo
rce all conflicts to become errors

library(readxl)
library(rstatix)

##
## Attaching package: 'rstatix'
##
## The following object is masked from 'package:stats':
##
##     filter

library(lme4)

## Loading required package: Matrix
##
## Attaching package: 'Matrix'
##
## The following objects are masked from 'package:tidyr':
```

```
##
##     expand, pack, unpack
```

```
library(lmerTest)
```

```
##
## Attaching package: 'lmerTest'
##
## The following object is masked from 'package:lme4':
##
##     lmer
##
## The following object is masked from 'package:stats':
##
##     step
```

```
library(lsmeans)
```

```
## Loading required package: emmeans
## The 'lsmeans' package is now basically a front end for 'emmeans'.
## Users are encouraged to switch the rest of the way.
## See help('transition') for more information, including how to
## convert old 'lsmeans' objects and scripts to work with 'emmeans'.
```

Uploading Data

```
bbd_polished <- read_excel("data/polished_bbd.xlsx", sheet = "polished
_bbd",
                           na = "NA")


bbd_polished_factors <- bbd_polished |>
                          mutate(tx = as.factor(tx)) |>
                          mutate(arm = as.factor(arm)) |>
                          mutate(appt_cat = as.factor(appt_cat)) |>
                          mutate(bb_first = as.factor(bb_first))

bbd_mid_filter <- bbd_polished_factors |>
                    filter(appt_cat != "mid" & HDRS_num != "NA")
```

HDRS Modeling and Evaluation

```
raw_HDRS_mem_int <- lmer(HDRS_num ~ tx +
                           arm +
                           tx : appt_cat +
                           bb_first +
                           appt_cat +
                           (1 | de_id),
```

```
                                 data = bbd_mid_filter)
print(raw_HDRS_mem_int)

## Linear mixed model fit by REML ['lmerModLmerTest']
## Formula: HDRS_num ~ tx + arm + tx:appt_cat + bb_first + appt_cat +
(1 |
##      de_id)
##     Data: bbd_mid_filter
## REML criterion at convergence: 728.8521
## Random effects:
##  Groups    Name        Std.Dev.
##  de_id     (Intercept) 5.213
##  Residual              4.512
## Number of obs: 117, groups:  de_id, 45
## Fixed Effects:
##             (Intercept)                txplacebo                   a
rm2
##                 22.3307                  -2.9580                 -3.7
301
##               bb_first1             appt_catpost  txplacebo:appt_catp
ost
##                 -0.6979                  -9.1643                  3.6
863

plot(raw_HDRS_mem_int)
```

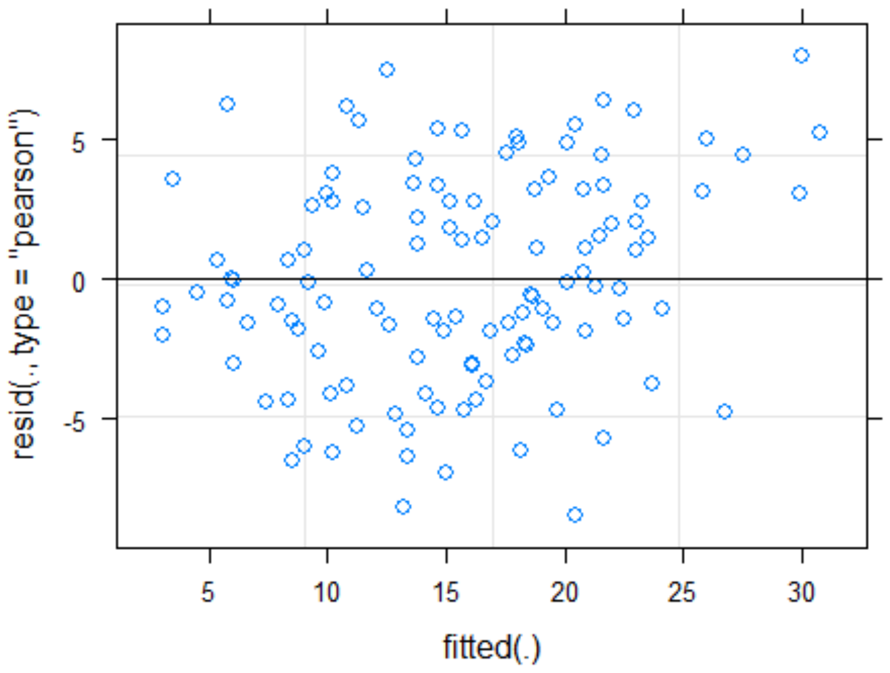

```
summary(raw_HDRS_mem_int)

## Linear mixed model fit by REML. t-tests use Satterthwaite's method
[
## lmerModLmerTest]
## Formula: HDRS_num ~ tx + arm + tx:appt_cat + bb_first + appt_cat +
(1 |
##     de_id)
##    Data: bbd_mid_filter
##
## REML criterion at convergence: 728.9
##
## Scaled residuals:
##      Min       1Q   Median       3Q      Max
## -1.87783 -0.58143 -0.03584  0.67659  1.76823
##
## Random effects:
##  Groups   Name        Variance Std.Dev.
##  de_id    (Intercept) 27.17    5.213
##  Residual             20.35    4.512
## Number of obs: 117, groups:  de_id, 45
##
## Fixed effects:
##                          Estimate Std. Error      df t value Pr(>|t|)
## (Intercept)               22.3307     1.5680 69.8573  14.242  < 2e-16
***
## txplacebo                 -2.9580     1.1893 78.8283  -2.487   0.0150
*
## arm2                      -3.7301     0.9048 74.5153  -4.122 9.63e-05
***
## bb_first1                 -0.6979     1.8670 41.2483  -0.374   0.7105
## appt_catpost              -9.1643     1.3048 76.5196  -7.024 7.70e-10
***
## txplacebo:appt_catpost     3.6863     1.7911 74.4646   2.058   0.0431
*
## ---
## Signif. codes:  0 '***' 0.001 '**' 0.01 '*' 0.05 '.' 0.1 ' ' 1
##
## Correlation of Fixed Effects:
##             (Intr) txplcb arm2   bb_fr1 appt_c
## txplacebo   -0.497
## arm2        -0.173 -0.005
## bb_first1   -0.674  0.198 -0.016
## appt_catpst -0.338  0.448  0.070  0.040
## txplcb:ppt_  0.315 -0.651 -0.068 -0.088 -0.725
```

```r
ls_means(raw_HDRS_mem_int)

## Least Squares Means table:
##
##                             Estimate Std. Error    df t value    lower
upper
## txblueberry                 15.53463    1.06116  64.1 14.6393 13.41478
17.65448
## txplacebo                   14.41979    1.03438  59.0 13.9405 12.35002
16.48956
## arm1                        16.84224    0.96638  50.2 17.4282 14.90141
18.78307
## arm2                        13.11218    1.12077  70.4 11.6993 10.87713
15.34724
## bb_first0                   15.32617    1.34553  42.5 11.3904 12.61171
18.04063
## bb_first1                   14.62825    1.30880  41.1 11.1768 11.98522
17.27128
## appt_catbl                  18.63776    0.97323  51.1 19.1503 16.68403
20.59148
## appt_catpost                11.31667    1.11311  69.3 10.1667  9.09621
13.53712
## txblueberry:appt_catbl      20.11676    1.14126  80.7 17.6268 17.84586
22.38765
## txplacebo:appt_catbl        17.15875    1.13979  80.5 15.0544 14.89073
19.42677
## txblueberry:appt_catpost 10.95250    1.34195 101.3  8.1616  8.29053
13.61447
## txplacebo:appt_catpost      11.68083    1.26598  95.0  9.2267  9.16756
14.19411
##                              Pr(>|t|)
## txblueberry                 < 2.2e-16 ***
## txplacebo                   < 2.2e-16 ***
## arm1                        < 2.2e-16 ***
## arm2                        < 2.2e-16 ***
## bb_first0                   1.699e-14 ***
## bb_first1                   4.950e-14 ***
## appt_catbl                  < 2.2e-16 ***
## appt_catpost                2.254e-15 ***
## txblueberry:appt_catbl      < 2.2e-16 ***
## txplacebo:appt_catbl        < 2.2e-16 ***
## txblueberry:appt_catpost 9.462e-13 ***
## txplacebo:appt_catpost      7.403e-15 ***
## ---
## Signif. codes:  0 '***' 0.001 '**' 0.01 '*' 0.05 '.' 0.1 ' ' 1
##
```

```
##   Confidence level: 95%
##   Degrees of freedom method: Satterthwaite
```

```r
ls_means(raw_HDRS_mem_int, pairwise = TRUE)
```

```
## Least Squares Means table:
##
##                                                     Estimate Std. Err
or   df
## txblueberry - txplacebo                              1.11484    0.911
45 72.9
## arm1 - arm2                                          3.73005    0.904
84 74.5
## bb_first0 - bb_first1                                0.69792    1.866
97 41.2
## appt_catbl - appt_catpost                            7.32109    0.900
64 75.0
## txblueberry:appt_catbl - txplacebo:appt_catbl        2.95801    1.189
31 78.8
## txblueberry:appt_catbl - txblueberry:appt_catpost  9.16426    1.304
77 76.5
## txblueberry:appt_catbl - txplacebo:appt_catpost    8.43593    1.247
33 73.8
## txplacebo:appt_catbl - txblueberry:appt_catpost    6.20625    1.314
51 74.1
## txplacebo:appt_catbl - txplacebo:appt_catpost      5.47792    1.234
45 72.8
## txblueberry:appt_catpost - txplacebo:appt_catpost -0.72833    1.360
52 69.8
##                                                    t value    lower
upper
## txblueberry - txplacebo                             1.2231 -0.70172
2.93139
## arm1 - arm2                                         4.1223  1.92732
5.53279
## bb_first0 - bb_first1                               0.3738 -3.07181
4.46764
## appt_catbl - appt_catpost                           8.1288  5.52694
9.11524
## txblueberry:appt_catbl - txplacebo:appt_catbl       2.4872  0.59067
5.32534
## txblueberry:appt_catbl - txblueberry:appt_catpost  7.0237  6.56588
11.76264
## txblueberry:appt_catbl - txplacebo:appt_catpost    6.7632  5.95044
10.92142
## txplacebo:appt_catbl - txblueberry:appt_catpost    4.7213  3.58710
```

```
8.82541
## txplacebo:appt_catbl - txplacebo:appt_catpost      4.4375  3.01755
7.93829
## txblueberry:appt_catpost - txplacebo:appt_catpost -0.5353 -3.44191
1.98524
##                                                     Pr(>|t|)
## txblueberry - txplacebo                              0.22521
## arm1 - arm2                                         9.634e-05 ***
## bb_first0 - bb_first1                                0.71045
## appt_catbl - appt_catpost                           6.769e-12 ***
## txblueberry:appt_catbl - txplacebo:appt_catbl        0.01499 *
## txblueberry:appt_catbl - txblueberry:appt_catpost 7.698e-10 ***
## txblueberry:appt_catbl - txplacebo:appt_catpost   2.740e-09 ***
## txplacebo:appt_catbl - txblueberry:appt_catpost   1.082e-05 ***
## txplacebo:appt_catbl - txplacebo:appt_catpost     3.172e-05 ***
## txblueberry:appt_catpost - txplacebo:appt_catpost   0.59412
## ---
## Signif. codes:  0 '***' 0.001 '**' 0.01 '*' 0.05 '.' 0.1 ' ' 1
##
##   Confidence level: 95%
##   Degrees of freedom method: Satterthwaite
```

```r
confint(raw_HDRS_mem_int)
```

```
## Computing profile confidence intervals ...
```

```
##                              2.5 %     97.5 %
## .sig01                   3.7200470  6.8211311
## .sigma                   3.7565772  5.2249223
## (Intercept)             19.3075999 25.3826924
## txplacebo               -5.2494463 -0.6542561
## arm2                    -5.4891810 -1.9875489
## bb_first1               -4.3585600  2.9366266
## appt_catpost           -11.6853440 -6.6452070
## txplacebo:appt_catpost   0.2097127  7.1370563
```

```r
HDRS_confint <- confint(raw_HDRS_mem_int)
```

```
## Computing profile confidence intervals ...
```

```r
coef(raw_HDRS_mem_int)$de_id
```

```
##    (Intercept) txplacebo      arm2  bb_first1 appt_catpost
## 1     17.83144 -2.958007 -3.730054 -0.6979158    -9.164259
## 2     23.69669 -2.958007 -3.730054 -0.6979158    -9.164259
## 3     17.42354 -2.958007 -3.730054 -0.6979158    -9.164259
## 4     19.48535 -2.958007 -3.730054 -0.6979158    -9.164259
## 5     23.10886 -2.958007 -3.730054 -0.6979158    -9.164259
```

```
## 6     15.90570 -2.958007 -3.730054 -0.6979158    -9.164259
## 7     18.36490 -2.958007 -3.730054 -0.6979158    -9.164259
## 8     23.06499 -2.958007 -3.730054 -0.6979158    -9.164259
## 9     16.37071 -2.958007 -3.730054 -0.6979158    -9.164259
## 10    24.58283 -2.958007 -3.730054 -0.6979158    -9.164259
## 11    18.22194 -2.958007 -3.730054 -0.6979158    -9.164259
## 12    18.85364 -2.958007 -3.730054 -0.6979158    -9.164259
## 13    31.48768 -2.958007 -3.730054 -0.6979158    -9.164259
## 14    27.48690 -2.958007 -3.730054 -0.6979158    -9.164259
## 15    15.90570 -2.958007 -3.730054 -0.6979158    -9.164259
## 16    23.83283 -2.958007 -3.730054 -0.6979158    -9.164259
## 17    32.98051 -2.958007 -3.730054 -0.6979158    -9.164259
## 18    30.64541 -2.958007 -3.730054 -0.6979158    -9.164259
## 19    18.68695 -2.958007 -3.730054 -0.6979158    -9.164259
## 20    17.25052 -2.958007 -3.730054 -0.6979158    -9.164259
## 21    24.49975 -2.958007 -3.730054 -0.6979158    -9.164259
## 22    18.59583 -2.958007 -3.730054 -0.6979158    -9.164259
## 23    23.43169 -2.958007 -3.730054 -0.6979158    -9.164259
## 24    21.83242 -2.958007 -3.730054 -0.6979158    -9.164259
## 25    26.68850 -2.958007 -3.730054 -0.6979158    -9.164259
## 26    21.16988 -2.958007 -3.730054 -0.6979158    -9.164259
## 27    22.05602 -2.958007 -3.730054 -0.6979158    -9.164259
## 28    26.06496 -2.958007 -3.730054 -0.6979158    -9.164259
## 29    18.64308 -2.958007 -3.730054 -0.6979158    -9.164259
## 30    25.42510 -2.958007 -3.730054 -0.6979158    -9.164259
## 31    24.97629 -2.958007 -3.730054 -0.6979158    -9.164259
## 32    21.54591 -2.958007 -3.730054 -0.6979158    -9.164259
## 33    21.96894 -2.958007 -3.730054 -0.6979158    -9.164259
## 34    24.25586 -2.958007 -3.730054 -0.6979158    -9.164259
## 35    20.25375 -2.958007 -3.730054 -0.6979158    -9.164259
## 36    28.25797 -2.958007 -3.730054 -0.6979158    -9.164259
## 37    24.40456 -2.958007 -3.730054 -0.6979158    -9.164259
## 38    28.97840 -2.958007 -3.730054 -0.6979158    -9.164259
## 39    24.25586 -2.958007 -3.730054 -0.6979158    -9.164259
## 40    17.39509 -2.958007 -3.730054 -0.6979158    -9.164259
## 41    15.82861 -2.958007 -3.730054 -0.6979158    -9.164259
## 42    26.54278 -2.958007 -3.730054 -0.6979158    -9.164259
## 43    19.11029 -2.958007 -3.730054 -0.6979158    -9.164259
## 44    19.83072 -2.958007 -3.730054 -0.6979158    -9.164259
## 45    23.68413 -2.958007 -3.730054 -0.6979158    -9.164259
##    txplacebo:appt_catpost
## 1                3.686339
## 2                3.686339
## 3                3.686339
## 4                3.686339
## 5                3.686339
```

```
## 6                    3.686339
## 7                    3.686339
## 8                    3.686339
## 9                    3.686339
## 10                   3.686339
## 11                   3.686339
## 12                   3.686339
## 13                   3.686339
## 14                   3.686339
## 15                   3.686339
## 16                   3.686339
## 17                   3.686339
## 18                   3.686339
## 19                   3.686339
## 20                   3.686339
## 21                   3.686339
## 22                   3.686339
## 23                   3.686339
## 24                   3.686339
## 25                   3.686339
## 26                   3.686339
## 27                   3.686339
## 28                   3.686339
## 29                   3.686339
## 30                   3.686339
## 31                   3.686339
## 32                   3.686339
## 33                   3.686339
## 34                   3.686339
## 35                   3.686339
## 36                   3.686339
## 37                   3.686339
## 38                   3.686339
## 39                   3.686339
## 40                   3.686339
## 41                   3.686339
## 42                   3.686339
## 43                   3.686339
## 44                   3.686339
## 45                   3.686339
```

GAD-7 Modeling and Evaluation

```
raw_GAD-7_mem_int <- lmer(GAD-7_num ~ tx +
                           arm +
                           tx : appt_cat +
                           bb_first +
```

```
                                  appt_cat +
                                  (1 | de_id),
                                data = bbd_polished_factors)
print(raw_GAD-7_mem_int)

## Linear mixed model fit by REML ['lmerModLmerTest']
## Formula: GAD-7_num ~ tx + arm + tx:appt_cat + bb_first + appt_cat +
(1 |
##     de_id)
##    Data: bbd_polished_factors
## REML criterion at convergence: 1088.878
## Random effects:
##  Groups   Name        Std.Dev.
##  de_id    (Intercept) 4.072
##  Residual             3.944
## Number of obs: 186, groups:  de_id, 45
## Fixed Effects:
##            (Intercept)               txplacebo                    a
rm2
##                 14.853                  -2.352                  -2.
118
##              bb_first1             appt_catmid            appt_catp
ost
##                 -1.148                  -3.678                  -5.
358
##  txplacebo:appt_catmid  txplacebo:appt_catpost
##                  2.336                   3.134

summary(raw_GAD-7_mem_int)

## Linear mixed model fit by REML. t-tests use Satterthwaite's method
[
## lmerModLmerTest]
## Formula: GAD-7_num ~ tx + arm + tx:appt_cat + bb_first + appt_cat +
(1 |
##     de_id)
##    Data: bbd_polished_factors
##
## REML criterion at convergence: 1088.9
##
## Scaled residuals:
##      Min       1Q   Median       3Q      Max
## -2.02631 -0.62665 -0.01669  0.57355  2.40590
##
## Random effects:
##  Groups   Name        Variance Std.Dev.
```

```
##  de_id    (Intercept) 16.58    4.072
##  Residual             15.55    3.944
## Number of obs: 186, groups:  de_id, 45
##
## Fixed effects:
##                          Estimate Std. Error       df t value Pr(>|t|
)
## (Intercept)               14.8533     1.2052  80.3263  12.324  < 2e-1
6 ***
## txplacebo                 -2.3520     0.9832 150.1047  -2.392 0.01798
6 *
## arm2                      -2.1180     0.6161 146.0900  -3.438 0.00076
4 ***
## bb_first1                 -1.1477     1.4080  42.9288  -0.815 0.41949
0
## appt_catmid               -3.6776     0.9732 143.5951  -3.779 0.00023
0 ***
## appt_catpost              -5.3579     1.0651 145.7807  -5.030 1.42e-0
6 ***
## txplacebo:appt_catmid      2.3363     1.4058 142.8232   1.662 0.09871
0 .
## txplacebo:appt_catpost     3.1336     1.4762 144.6842   2.123 0.03547
7 *
## ---
## Signif. codes:  0 '***' 0.001 '**' 0.01 '*' 0.05 '.' 0.1 ' ' 1
##
## Correlation of Fixed Effects:
##                 (Intr) txplcb arm2   bb_fr1 appt_ctm appt_ctp txplcb
:ppt_ctm
## txplacebo      -0.463
## arm2           -0.218  0.048
## bb_first1      -0.645  0.137  0.030
## appt_catmid    -0.367  0.450  0.024  0.029
## appt_catpst    -0.355  0.438  0.054  0.044  0.430
## txplcb:ppt_ctm  0.284 -0.668 -0.014 -0.046 -0.689   -0.297
## txplcb:ppt_ctp  0.288 -0.660 -0.033 -0.058 -0.310   -0.723    0.453
```

```
ls_means(raw_GAD-7_mem_int)
```

```
## Least Squares Means table:
##
##                            Estimate Std. Error    df t value    lower
upper
## txblueberry                10.20854    0.76848  59.6 13.2841  8.67115
11.74594
## txplacebo                   9.67982    0.77816  61.0 12.4394  8.12380
```

```
11.23585
## arm1                     11.00319    0.72951  51.3 15.0830  9.53882
12.46756
## arm2                      8.88518    0.81379  68.4 10.9182  7.26147
10.50889
## bb_first0                10.51805    0.99688  41.8 10.5509  8.50600
12.53011
## bb_first1                 9.37032    1.00108  44.8  9.3602  7.35378
11.38685
## appt_catbl               12.04438    0.77586  65.5 15.5239 10.49508
13.59367
## appt_catmid               9.53492    0.83138  76.1 11.4687  7.87911
11.19072
## appt_catpost              8.25326    0.86612  84.7  9.5290  6.53109
9.97542
## txblueberry:appt_catbl   13.22039    0.89395 101.1 14.7888 11.44705
14.99373
## txplacebo:appt_catbl     10.86836    0.94241 113.1 11.5325  9.00130
12.73543
## txblueberry:appt_catmid   9.54276    0.96916 118.4  9.8464  7.62363
11.46189
## txplacebo:appt_catmid     9.52707    0.99529 124.2  9.5722  7.55715
11.49700
## txblueberry:appt_catpost  7.86248    1.06001 139.0  7.4174  5.76667
9.95829
## txplacebo:appt_catpost    8.64404    0.99578 124.1  8.6807  6.67313
10.61494
##                           Pr(>|t|)
## txblueberry              < 2.2e-16 ***
## txplacebo                < 2.2e-16 ***
## arm1                     < 2.2e-16 ***
## arm2                     < 2.2e-16 ***
## bb_first0                2.333e-13 ***
## bb_first1                4.141e-12 ***
## appt_catbl               < 2.2e-16 ***
## appt_catmid              < 2.2e-16 ***
## appt_catpost             4.730e-15 ***
## txblueberry:appt_catbl   < 2.2e-16 ***
## txplacebo:appt_catbl     < 2.2e-16 ***
## txblueberry:appt_catmid  < 2.2e-16 ***
## txplacebo:appt_catmid    < 2.2e-16 ***
## txblueberry:appt_catpost 1.071e-11 ***
## txplacebo:appt_catpost   1.872e-14 ***
## ---
## Signif. codes:  0 '***' 0.001 '**' 0.01 '*' 0.05 '.' 0.1 ' ' 1
##
```

```
##   Confidence level: 95%
##   Degrees of freedom method: Satterthwaite
```

```
GAD-7_lsmeans <- ls_means(raw_GAD-7_mem_int)

ls_means(raw_GAD-7_mem_int, pairwise = TRUE)
```

```
## Least Squares Means table:
##
##                                                         Estimate Std. E
rror    df
## txblueberry - txplacebo                                 0.528720   0.61
8737 144.3
## arm1 - arm2                                             2.118010   0.61
6070 146.1
## bb_first0 - bb_first1                                   1.147737   1.40
8019  42.9
## appt_catbl - appt_catmid                                2.509457   0.70
6017 143.5
## appt_catbl - appt_catpost                               3.791116   0.73
6353 144.3
## appt_catmid - appt_catpost                              1.281659   0.75
3841 139.0
## txblueberry:appt_catbl - txplacebo:appt_catbl           2.352026   0.98
3214 150.1
## txblueberry:appt_catbl - txblueberry:appt_catmid        3.677626   0.97
3248 143.6
## txblueberry:appt_catbl - txplacebo:appt_catmid          3.693315   1.00
8883 145.4
## txblueberry:appt_catbl - txblueberry:appt_catpost       5.357907   1.06
5121 145.8
## txblueberry:appt_catbl - txplacebo:appt_catpost         4.576352   1.00
4158 144.5
## txplacebo:appt_catbl - txblueberry:appt_catmid          1.325599   1.02
5988 145.7
## txplacebo:appt_catbl - txplacebo:appt_catmid            1.341289   1.01
8716 142.8
## txplacebo:appt_catbl - txblueberry:appt_catpost         3.005880   1.08
7638 143.0
## txplacebo:appt_catbl - txplacebo:appt_catpost          2.224325   1.01
9563 143.1
## txblueberry:appt_catmid - txplacebo:appt_catmid         0.015689   1.04
6394 140.9
## txblueberry:appt_catmid - txblueberry:appt_catpost      1.680281   1.09
0576 140.1
## txblueberry:appt_catmid - txplacebo:appt_catpost        0.898726   1.04
```

```
5374 140.6
## txplacebo:appt_catmid - txblueberry:appt_catpost    1.664592   1.10
9842 139.1
## txplacebo:appt_catmid - txplacebo:appt_catpost      0.883037   1.04
1078 137.9
## txblueberry:appt_catpost - txplacebo:appt_catpost  -0.781555   1.10
8922 138.8
##                                                    t value     lowe
r     upper
## txblueberry - txplacebo                             0.8545 -0.69424
0  1.751680
## arm1 - arm2                                         3.4379  0.90044
9  3.335572
## bb_first0 - bb_first1                               0.8151 -1.69194
1  3.987415
## appt_catbl - appt_catmid                            3.5544  1.11392
3  3.904991
## appt_catbl - appt_catpost                           5.1485  2.33568
6  5.246546
## appt_catmid - appt_catpost                          1.7002 -0.20881
6  2.772134
## txblueberry:appt_catbl - txplacebo:appt_catbl       2.3922  0.40929
9  4.294753
## txblueberry:appt_catbl - txblueberry:appt_catmid    3.7787  1.75388
3  5.601369
## txblueberry:appt_catbl - txplacebo:appt_catmid      3.6608  1.69934
9  5.687281
## txblueberry:appt_catbl - txblueberry:appt_catpost   5.0303  3.25283
3  7.462980
## txblueberry:appt_catbl - txplacebo:appt_catpost     4.5574  2.59162
1  6.561083
## txplacebo:appt_catbl - txblueberry:appt_catmid      1.2920 -0.70213
9  3.353337
## txplacebo:appt_catbl - txplacebo:appt_catmid        1.3166 -0.67242
4  3.355002
## txplacebo:appt_catbl - txblueberry:appt_catpost     2.7637  0.85594
9  5.155812
## txplacebo:appt_catbl - txplacebo:appt_catpost       2.1816  0.20897
5  4.239676
## txblueberry:appt_catmid - txplacebo:appt_catmid     0.0150 -2.05297
6  2.084355
## txblueberry:appt_catmid - txblueberry:appt_catpost  1.5407 -0.47584
0  3.836402
## txblueberry:appt_catmid - txplacebo:appt_catpost    0.8597 -1.16796
1  2.965413
## txplacebo:appt_catmid - txblueberry:appt_catpost    1.4998 -0.52974
```

```
9  3.858932
## txplacebo:appt_catmid - txplacebo:appt_catpost      0.8482 -1.17549
9  2.941573
## txblueberry:appt_catpost - txplacebo:appt_catpost  -0.7048 -2.97411
2  1.411002
##                                                     Pr(>|t|)
## txblueberry - txplacebo                            0.3942360
## arm1 - arm2                                        0.0007639 ***
## bb_first0 - bb_first1                              0.4194902
## appt_catbl - appt_catmid                           0.0005134 ***
## appt_catbl - appt_catpost                          8.452e-07 ***
## appt_catmid - appt_catpost                         0.0913347 .
## txblueberry:appt_catbl - txplacebo:appt_catbl      0.0179858 *
## txblueberry:appt_catbl - txblueberry:appt_catmid   0.0002304 ***
## txblueberry:appt_catbl - txplacebo:appt_catmid     0.0003511 ***
## txblueberry:appt_catbl - txblueberry:appt_catpost  1.419e-06 ***
## txblueberry:appt_catbl - txplacebo:appt_catpost    1.094e-05 ***
## txplacebo:appt_catbl - txblueberry:appt_catmid     0.1983946
## txplacebo:appt_catbl - txplacebo:appt_catmid       0.1900666
## txplacebo:appt_catbl - txblueberry:appt_catpost    0.0064682 **
## txplacebo:appt_catbl - txplacebo:appt_catpost      0.0307682 *
## txblueberry:appt_catmid - txplacebo:appt_catmid    0.9880585
## txblueberry:appt_catmid - txblueberry:appt_catpost 0.1256394
## txblueberry:appt_catmid - txplacebo:appt_catpost   0.3914087
## txplacebo:appt_catmid - txblueberry:appt_catpost   0.1359205
## txplacebo:appt_catmid - txplacebo:appt_catpost     0.3977992
## txblueberry:appt_catpost - txplacebo:appt_catpost  0.4821226
## ---
## Signif. codes:  0 '***' 0.001 '**' 0.01 '*' 0.05 '.' 0.1 ' ' 1
##
##   Confidence level: 95%
##   Degrees of freedom method: Satterthwaite
```

```
confint(raw_GAD-7_mem_int)
```

```
## Computing profile confidence intervals ...
```

```
##                             2.5 %     97.5 %
## .sig01                  3.0324074  5.2158437
## .sigma                  3.4532686  4.3567403
## (Intercept)            12.5152924 17.1825652
## txplacebo              -4.2542973 -0.4563242
## arm2                   -3.3085073 -0.9290167
## bb_first1              -3.8909800  1.6090538
## appt_catmid            -5.5719947 -1.8021369
## appt_catpost           -7.4264097 -3.3053762
```

```
## txplacebo:appt_catmid  -0.3738379  5.0603749
## txplacebo:appt_catpost  0.2886170  5.9978290
```

```
coef(raw_GAD-7_mem_int)$de_id
```

```
##    (Intercept) txplacebo     arm2 bb_first1 appt_catmid appt_catpos
t
## 1    11.364164 -2.352026 -2.11801 -1.147737   -3.677626    -5.35790
7
## 2    12.515194 -2.352026 -2.11801 -1.147737   -3.677626    -5.35790
7
## 3    13.108086 -2.352026 -2.11801 -1.147737   -3.677626    -5.35790
7
## 4    11.362151 -2.352026 -2.11801 -1.147737   -3.677626    -5.35790
7
## 5    15.558302 -2.352026 -2.11801 -1.147737   -3.677626    -5.35790
7
## 6    12.226934 -2.352026 -2.11801 -1.147737   -3.677626    -5.35790
7
## 7    11.927599 -2.352026 -2.11801 -1.147737   -3.677626    -5.35790
7
## 8    16.118453 -2.352026 -2.11801 -1.147737   -3.677626    -5.35790
7
## 9    11.234391 -2.352026 -2.11801 -1.147737   -3.677626    -5.35790
7
## 10   15.125911 -2.352026 -2.11801 -1.147737   -3.677626    -5.35790
7
## 11   11.650412 -2.352026 -2.11801 -1.147737   -3.677626    -5.35790
7
## 12   18.712800 -2.352026 -2.11801 -1.147737   -3.677626    -5.35790
7
## 13   22.316059 -2.352026 -2.11801 -1.147737   -3.677626    -5.35790
7
## 14   16.406714 -2.352026 -2.11801 -1.147737   -3.677626    -5.35790
7
## 15   12.082803 -2.352026 -2.11801 -1.147737   -3.677626    -5.35790
7
## 16   21.900038 -2.352026 -2.11801 -1.147737   -3.677626    -5.35790
7
## 17   21.832811 -2.352026 -2.11801 -1.147737   -3.677626    -5.35790
7
## 18   14.388889 -2.352026 -2.11801 -1.147737   -3.677626    -5.35790
7
## 19    9.937218 -2.352026 -2.11801 -1.147737   -3.677626    -5.35790
7
## 20    7.701507 -2.352026 -2.11801 -1.147737   -3.677626    -5.35790
```

```
7
## 21   15.729429 -2.352026 -2.11801 -1.147737   -3.677626    -5.35790
7
## 22   12.540936 -2.352026 -2.11801 -1.147737   -3.677626    -5.35790
7
## 23   14.109393 -2.352026 -2.11801 -1.147737   -3.677626    -5.35790
7
## 24   19.577013 -2.352026 -2.11801 -1.147737   -3.677626    -5.35790
7
## 25   19.445428 -2.352026 -2.11801 -1.147737   -3.677626    -5.35790
7
## 26   20.874756 -2.352026 -2.11801 -1.147737   -3.677626    -5.35790
7
## 27   12.531565 -2.352026 -2.11801 -1.147737   -3.677626    -5.35790
7
## 28   14.832683 -2.352026 -2.11801 -1.147737   -3.677626    -5.35790
7
## 29   12.659325 -2.352026 -2.11801 -1.147737   -3.677626    -5.35790
7
## 30   12.675695 -2.352026 -2.11801 -1.147737   -3.677626    -5.35790
7
## 31   15.990693 -2.352026 -2.11801 -1.147737   -3.677626    -5.35790
7
## 32   17.174411 -2.352026 -2.11801 -1.147737   -3.677626    -5.35790
7
## 33   18.100912 -2.352026 -2.11801 -1.147737   -3.677626    -5.35790
7
## 34   15.521150 -2.352026 -2.11801 -1.147737   -3.677626    -5.35790
7
## 35   14.603642 -2.352026 -2.11801 -1.147737   -3.677626    -5.35790
7
## 36   18.347478 -2.352026 -2.11801 -1.147737   -3.677626    -5.35790
7
## 37   14.078696 -2.352026 -2.11801 -1.147737   -3.677626    -5.35790
7
## 38   18.722268 -2.352026 -2.11801 -1.147737   -3.677626    -5.35790
7
## 39   10.519458 -2.352026 -2.11801 -1.147737   -3.677626    -5.35790
7
## 40   16.037102 -2.352026 -2.11801 -1.147737   -3.677626    -5.35790
7
## 41   11.224743 -2.352026 -2.11801 -1.147737   -3.677626    -5.35790
7
## 42   17.069007 -2.352026 -2.11801 -1.147737   -3.677626    -5.35790
7
## 43   11.540504 -2.352026 -2.11801 -1.147737   -3.677626    -5.35790
```

```
7
## 44   10.982981 -2.352026 -2.11801 -1.147737   -3.677626    -5.35790
7
## 45   16.037102 -2.352026 -2.11801 -1.147737   -3.677626    -5.35790
7
##    txplacebo:appt_catmid txplacebo:appt_catpost
## 1               2.336337               3.133581
## 2               2.336337               3.133581
## 3               2.336337               3.133581
## 4               2.336337               3.133581
## 5               2.336337               3.133581
## 6               2.336337               3.133581
## 7               2.336337               3.133581
## 8               2.336337               3.133581
## 9               2.336337               3.133581
## 10              2.336337               3.133581
## 11              2.336337               3.133581
## 12              2.336337               3.133581
## 13              2.336337               3.133581
## 14              2.336337               3.133581
## 15              2.336337               3.133581
## 16              2.336337               3.133581
## 17              2.336337               3.133581
## 18              2.336337               3.133581
## 19              2.336337               3.133581
## 20              2.336337               3.133581
## 21              2.336337               3.133581
## 22              2.336337               3.133581
## 23              2.336337               3.133581
## 24              2.336337               3.133581
## 25              2.336337               3.133581
## 26              2.336337               3.133581
## 27              2.336337               3.133581
## 28              2.336337               3.133581
## 29              2.336337               3.133581
## 30              2.336337               3.133581
## 31              2.336337               3.133581
## 32              2.336337               3.133581
## 33              2.336337               3.133581
## 34              2.336337               3.133581
## 35              2.336337               3.133581
## 36              2.336337               3.133581
## 37              2.336337               3.133581
## 38              2.336337               3.133581
## 39              2.336337               3.133581
## 40              2.336337               3.133581
```

```
## 41                2.336337                3.133581
## 42                2.336337                3.133581
## 43                2.336337                3.133581
## 44                2.336337                3.133581
## 45                2.336337                3.133581
```

```r
plot(raw_GAD-7_mem_int)
```

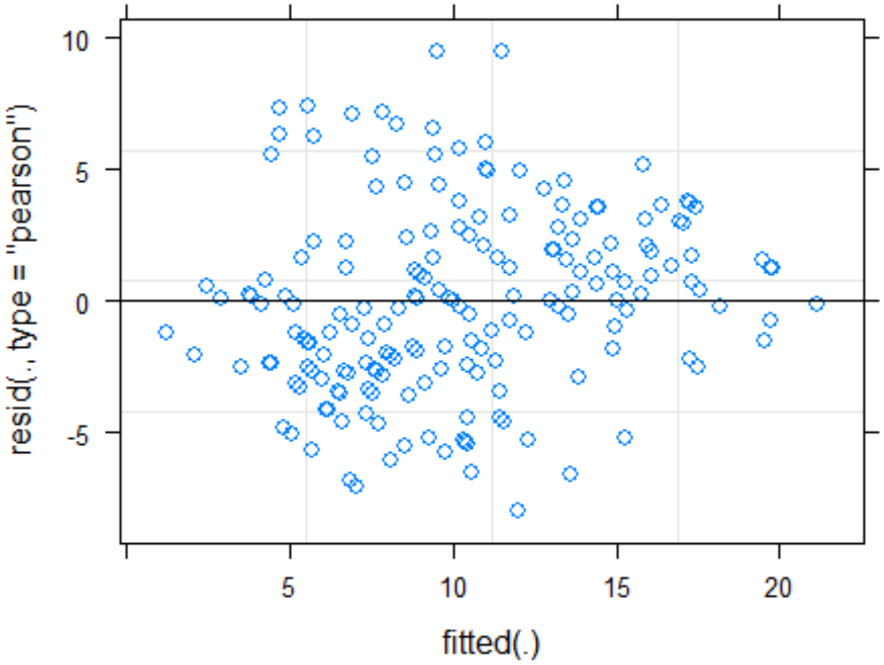

MDI Modeling and Evaluation

```r
raw_mdi_mem_int <- lmer(mdi_num ~ tx +
                           arm +
                           tx : appt_cat +
                           bb_first +
                           appt_cat +
                           (1 | de_id),
                         data = bbd_polished_factors)
print(raw_mdi_mem_int)
```

```
## Linear mixed model fit by REML ['lmerModLmerTest']
## Formula: mdi_num ~ tx + arm + tx:appt_cat + bb_first + appt_cat + (
1 |
##     de_id)
##    Data: bbd_polished_factors
## REML criterion at convergence: 1270.898
## Random effects:
```

```
##  Groups   Name        Std.Dev.
##  de_id    (Intercept) 8.668
##  Residual             6.351
## Number of obs: 185, groups:  de_id, 45
## Fixed Effects:
##            (Intercept)               txplacebo                    a
rm2
##                 26.481                  -1.820                  -4.
071
##              bb_first1             appt_catmid            appt_catp
ost
##                 -2.493                  -6.113                  -6.
716
##  txplacebo:appt_catmid  txplacebo:appt_catpost
##                  2.310                   0.564
```

```
summary(raw_mdi_mem_int)
```

```
## Linear mixed model fit by REML. t-tests use Satterthwaite's method
[
## lmerModLmerTest]
## Formula: mdi_num ~ tx + arm + tx:appt_cat + bb_first + appt_cat + (
1 |
##     de_id)
##    Data: bbd_polished_factors
##
## REML criterion at convergence: 1270.9
##
## Scaled residuals:
##     Min      1Q  Median      3Q     Max
## -2.0675 -0.6135 -0.0132  0.5507  3.9333
##
## Random effects:
##  Groups   Name        Variance Std.Dev.
##  de_id    (Intercept) 75.13    8.668
##  Residual             40.33    6.351
## Number of obs: 185, groups:  de_id, 45
##
## Fixed effects:
##                        Estimate Std. Error      df t value Pr(>|t|)
## (Intercept)              26.481      2.316  70.346  11.436  < 2e-16
***
## txplacebo                -1.820      1.601 145.936  -1.137 0.257496
## arm2                     -4.071      1.011 142.769  -4.028 9.09e-05
***
## bb_first1                -2.493      2.847  44.345  -0.876 0.385993
```

```
## appt_catmid              -6.114      1.584 141.045  -3.860 0.000172
***
## appt_catpost             -6.716      1.711 142.294  -3.926 0.000134
***
## txplacebo:appt_catmid     2.310      2.295 140.305   1.006 0.316011
## txplacebo:appt_catpost    0.564      2.365 141.220   0.238 0.811876
## ---
## Signif. codes:  0 '***' 0.001 '**' 0.01 '*' 0.05 '.' 0.1 ' ' 1
##
## Correlation of Fixed Effects:
##                (Intr) txplcb arm2   bb_fr1 appt_ctm appt_ctp txplcb
:ppt_ctm
## txplacebo      -0.415
## arm2           -0.193  0.069
## bb_first1      -0.667  0.127  0.031
## appt_catmid    -0.320  0.464  0.040  0.029
## appt_catpst    -0.316  0.458  0.060  0.047  0.447
## txplcb:ppt_ctm  0.243 -0.657 -0.006 -0.039 -0.686   -0.304
## txplcb:ppt_ctp  0.261 -0.666 -0.040 -0.061 -0.323   -0.723    0.452
```

```
ls_means(raw_mdi_mem_int)
```

```
## Least Squares Means table:
##
##                            Estimate Std. Error    df t value   lower
upper
## txblueberry                 18.9227     1.5055  54.8 12.5691 15.9054
21.9400
## txplacebo                   18.0605     1.5291  57.4 11.8116 14.9991
21.1219
## arm1                        20.5271     1.4511  48.7 14.1458 17.6106
23.4436
## arm2                        16.4560     1.5802  63.2 10.4142 13.2986
19.6135
## bb_first0                   19.7380     2.0189  43.1  9.7767 15.6669
23.8091
## bb_first1                   17.2451     2.0173  46.2  8.5486 13.1850
21.3052
## appt_catbl                  22.2892     1.5105  57.3 14.7558 19.2647
25.3136
## appt_catmid                 17.3305     1.6067  68.2 10.7864 14.1246
20.5365
## appt_catpost                15.8550     1.6374  72.1  9.6830 12.5910
19.1191
## txblueberry:appt_catbl      23.1992     1.6842  82.6 13.7747 19.8492
26.5492
```

```
## txplacebo:appt_catbl      21.3791     1.7346  89.5 12.3253 17.9328
24.8254
## txblueberry:appt_catmid   17.0858     1.7858  96.1  9.5676 13.5410
20.6305
## txplacebo:appt_catmid     17.5753     1.8641 107.2  9.4281 13.8800
21.2707
## txblueberry:appt_catpost  16.4831     1.8947 111.5  8.6994 12.7287
20.2374
## txplacebo:appt_catpost    15.2270     1.8249 101.4  8.3440 11.6070
18.8469
##                           Pr(>|t|)
## txblueberry              < 2.2e-16 ***
## txplacebo                < 2.2e-16 ***
## arm1                     < 2.2e-16 ***
## arm2                     2.396e-15 ***
## bb_first0                1.648e-12 ***
## bb_first1                4.483e-11 ***
## appt_catbl               < 2.2e-16 ***
## appt_catmid              < 2.2e-16 ***
## appt_catpost             1.121e-14 ***
## txblueberry:appt_catbl   < 2.2e-16 ***
## txplacebo:appt_catbl     < 2.2e-16 ***
## txblueberry:appt_catmid  1.254e-15 ***
## txplacebo:appt_catmid    9.943e-16 ***
## txblueberry:appt_catpost 3.348e-14 ***
## txplacebo:appt_catpost   3.784e-13 ***
## ---
## Signif. codes:  0 '***' 0.001 '**' 0.01 '*' 0.05 '.' 0.1 ' ' 1
##
##   Confidence level: 95%
##   Degrees of freedom method: Satterthwaite
```

```
plot(raw_mdi_mem_int)
```

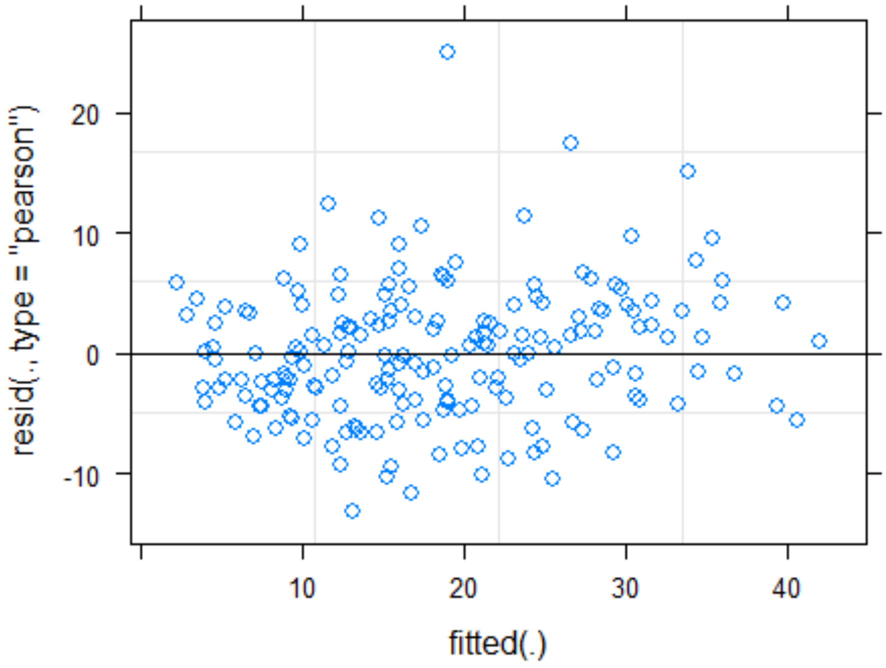


```
mdi_lsmeans <- ls_means(raw_mdi_mem_int)
ls_means(raw_mdi_mem_int, pairwise = TRUE)

## Least Squares Means table:
##
##                                                          Estimate Std. Er
ror    df
## txblueberry - txplacebo                                   0.86221    1.01
247 141.4
## arm1 - arm2                                               4.07108    1.01
060 142.8
## bb_first0 - bb_first1                                     2.49292    2.84
726  44.3
## appt_catbl - appt_catmid                                  4.95862    1.15
370 140.8
## appt_catbl - appt_catpost                                 6.43415    1.18
296 141.2
## appt_catmid - appt_catpost                                1.47553    1.22
015 137.1
## txblueberry:appt_catbl - txplacebo:appt_catbl             1.82011    1.60
112 145.9
## txblueberry:appt_catbl - txblueberry:appt_catmid          6.11346    1.58
366 141.0
## txblueberry:appt_catbl - txplacebo:appt_catmid            5.62388    1.69
536 142.6
```

```
## txblueberry:appt_catbl - txblueberry:appt_catpost   6.71616    1.71
056 142.3
## txblueberry:appt_catbl - txplacebo:appt_catpost     7.97225    1.64
271 142.1
## txplacebo:appt_catbl - txblueberry:appt_catmid      4.29336    1.64
907 141.8
## txplacebo:appt_catbl - txplacebo:appt_catmid        3.80378    1.66
979 140.1
## txplacebo:appt_catbl - txblueberry:appt_catpost     4.89605    1.72
707 140.1
## txplacebo:appt_catbl - txplacebo:appt_catpost       6.15215    1.63
405 140.1
## txblueberry:appt_catmid - txplacebo:appt_catmid    -0.48958    1.73
267 138.6
## txblueberry:appt_catmid - txblueberry:appt_catpost  0.60269    1.73
619 137.6
## txblueberry:appt_catmid - txplacebo:appt_catpost    1.85879    1.68
909 138.2
## txplacebo:appt_catmid - txblueberry:appt_catpost    1.09228    1.80
674 137.2
## txplacebo:appt_catmid - txplacebo:appt_catpost      2.34837    1.71
516 136.6
## txblueberry:appt_catpost - txplacebo:appt_catpost   1.25610    1.76
529 136.9
##                                                    t value    lower
upper
## txblueberry - txplacebo                             0.8516 -1.13933
2.86374
## arm1 - arm2                                         4.0284  2.07341
6.06874
## bb_first0 - bb_first1                               0.8755 -3.24410
8.22993
## appt_catbl - appt_catmid                            4.2980  2.67781
7.23943
## appt_catbl - appt_catpost                           5.4390  4.09555
8.77276
## appt_catmid - appt_catpost                          1.2093 -0.93721
3.88827
## txblueberry:appt_catbl - txplacebo:appt_catbl       1.1368 -1.34426
4.98448
## txblueberry:appt_catbl - txblueberry:appt_catmid    3.8603  2.98269
9.24424
## txblueberry:appt_catbl - txplacebo:appt_catmid      3.3172  2.27261
8.97516
## txblueberry:appt_catbl - txblueberry:appt_catpost   3.9263  3.33476
10.09756
```

```
## txblueberry:appt_catbl - txplacebo:appt_catpost     4.8531  4.72494
11.21957
## txplacebo:appt_catbl - txblueberry:appt_catmid      2.6035  1.03342
7.55330
## txplacebo:appt_catbl - txplacebo:appt_catmid        2.2780  0.50254
7.10501
## txplacebo:appt_catbl - txblueberry:appt_catpost     2.8349  1.48154
8.31056
## txplacebo:appt_catbl - txplacebo:appt_catpost       3.7650  2.92156
9.38273
## txblueberry:appt_catmid - txplacebo:appt_catmid    -0.2826 -3.91548
2.93631
## txblueberry:appt_catmid - txblueberry:appt_catpost  0.3471 -2.83036
4.03575
## txblueberry:appt_catmid - txplacebo:appt_catpost    1.1005 -1.48100
5.19858
## txplacebo:appt_catmid - txblueberry:appt_catpost    0.6046 -2.48037
4.66492
## txplacebo:appt_catmid - txplacebo:appt_catpost      1.3692 -1.04332
5.74006
## txblueberry:appt_catpost - txplacebo:appt_catpost   0.7116 -2.23466
4.74686
##                                                     Pr(>|t|)
## txblueberry - txplacebo                            0.3958820
## arm1 - arm2                                        9.090e-05 ***
## bb_first0 - bb_first1                              0.3859927
## appt_catbl - appt_catmid                           3.197e-05 ***
## appt_catbl - appt_catpost                          2.297e-07 ***
## appt_catmid - appt_catpost                         0.2286261
## txblueberry:appt_catbl - txplacebo:appt_catbl      0.2574960
## txblueberry:appt_catbl - txblueberry:appt_catmid   0.0001718 ***
## txblueberry:appt_catbl - txplacebo:appt_catmid     0.0011535 **
## txblueberry:appt_catbl - txblueberry:appt_catpost  0.0001339 ***
## txblueberry:appt_catbl - txplacebo:appt_catpost    3.160e-06 ***
## txplacebo:appt_catbl - txblueberry:appt_catmid     0.0102095 *
## txplacebo:appt_catbl - txplacebo:appt_catmid       0.0242396 *
## txplacebo:appt_catbl - txblueberry:appt_catpost    0.0052632 **
## txplacebo:appt_catbl - txplacebo:appt_catpost      0.0002444 ***
## txblueberry:appt_catmid - txplacebo:appt_catmid    0.7779373
## txblueberry:appt_catmid - txblueberry:appt_catpost 0.7290191
## txblueberry:appt_catmid - txplacebo:appt_catpost   0.2730398
## txplacebo:appt_catmid - txblueberry:appt_catpost   0.5464721
## txplacebo:appt_catmid - txplacebo:appt_catpost     0.1731873
## txblueberry:appt_catpost - txplacebo:appt_catpost  0.4779520
## ---
## Signif. codes:  0 '***' 0.001 '**' 0.01 '*' 0.05 '.' 0.1 ' ' 1
```

```
##
##   Confidence level: 95%
##   Degrees of freedom method: Satterthwaite
```

```r
confint(raw_mdi_mem_int)
```

```
## Computing profile confidence intervals ...
```

```
##                                 2.5 %    97.5 %
## .sig01                       6.681685 10.881718
## .sigma                       5.556403  7.016633
## (Intercept)                 21.990806 30.973643
## txplacebo                   -4.913746  1.268403
## arm2                        -6.033352 -2.123686
## bb_first1                   -8.058206  3.064729
## appt_catmid                 -9.181874 -3.060955
## appt_catpost               -10.038344 -3.419962
## txplacebo:appt_catmid       -2.120182  6.744470
## txplacebo:appt_catpost      -3.995069  5.147035
```

```r
raw_GAD-7_mem_int
```

```
## Linear mixed model fit by REML ['lmerModLmerTest']
## Formula: GAD-7_num ~ tx + arm + tx:appt_cat + bb_first + appt_cat +
(1 |
##     de_id)
##    Data: bbd_polished_factors
## REML criterion at convergence: 1088.878
## Random effects:
##  Groups   Name        Std.Dev.
##  de_id    (Intercept) 4.072
##  Residual             3.944
## Number of obs: 186, groups:  de_id, 45
## Fixed Effects:
##            (Intercept)               txplacebo                    a
rm2
##                 14.853                  -2.352                  -2.
118
##              bb_first1             appt_catmid             appt_catp
ost
##                 -1.148                  -3.678                  -5.
358
##  txplacebo:appt_catmid  txplacebo:appt_catpost
##                  2.336                   3.134
```

```r
coef(raw_mdi_mem_int)$de_id
```

```
##    (Intercept) txplacebo      arm2 bb_first1 appt_catmid appt_catpo
st
## 1     24.90374 -1.820107 -4.071077 -2.492918   -6.113463    -6.7161
58
## 2     17.99533 -1.820107 -4.071077 -2.492918   -6.113463    -6.7161
58
## 3     15.40118 -1.820107 -4.071077 -2.492918   -6.113463    -6.7161
58
## 4     21.93396 -1.820107 -4.071077 -2.492918   -6.113463    -6.7161
58
## 5     25.34485 -1.820107 -4.071077 -2.492918   -6.113463    -6.7161
58
## 6     18.45427 -1.820107 -4.071077 -2.492918   -6.113463    -6.7161
58
## 7     20.75546 -1.820107 -4.071077 -2.492918   -6.113463    -6.7161
58
## 8     24.42047 -1.820107 -4.071077 -2.492918   -6.113463    -6.7161
58
## 9     17.23693 -1.820107 -4.071077 -2.492918   -6.113463    -6.7161
58
## 10    25.49783 -1.820107 -4.071077 -2.492918   -6.113463    -6.7161
58
## 11    16.69629 -1.820107 -4.071077 -2.492918   -6.113463    -6.7161
58
## 12    17.38341 -1.820107 -4.071077 -2.492918   -6.113463    -6.7161
58
## 13    39.25949 -1.820107 -4.071077 -2.492918   -6.113463    -6.7161
58
## 14    41.86015 -1.820107 -4.071077 -2.492918   -6.113463    -6.7161
58
## 15    21.05492 -1.820107 -4.071077 -2.492918   -6.113463    -6.7161
58
## 16    32.38191 -1.820107 -4.071077 -2.492918   -6.113463    -6.7161
58
## 17    43.84704 -1.820107 -4.071077 -2.492918   -6.113463    -6.7161
58
## 18    43.08398 -1.820107 -4.071077 -2.492918   -6.113463    -6.7161
58
## 19    14.63628 -1.820107 -4.071077 -2.492918   -6.113463    -6.7161
58
## 20    13.20487 -1.820107 -4.071077 -2.492918   -6.113463    -6.7161
58
## 21    37.21758 -1.820107 -4.071077 -2.492918   -6.113463    -6.7161
58
## 22    23.09764 -1.820107 -4.071077 -2.492918   -6.113463    -6.7161
58
```

```
## 23    28.36854 -1.820107 -4.071077 -2.492918   -6.113463    -6.7161
58
## 24    33.43817 -1.820107 -4.071077 -2.492918   -6.113463    -6.7161
58
## 25    26.10975 -1.820107 -4.071077 -2.492918   -6.113463    -6.7161
58
## 26    24.58800 -1.820107 -4.071077 -2.492918   -6.113463    -6.7161
58
## 27    22.59122 -1.820107 -4.071077 -2.492918   -6.113463    -6.7161
58
## 28    27.29119 -1.820107 -4.071077 -2.492918   -6.113463    -6.7161
58
## 29    21.51386 -1.820107 -4.071077 -2.492918   -6.113463    -6.7161
58
## 30    29.16934 -1.820107 -4.071077 -2.492918   -6.113463    -6.7161
58
## 31    26.72167 -1.820107 -4.071077 -2.492918   -6.113463    -6.7161
58
## 32    27.35242 -1.820107 -4.071077 -2.492918   -6.113463    -6.7161
58
## 33    26.48883 -1.820107 -4.071077 -2.492918   -6.113463    -6.7161
58
## 34    29.09161 -1.820107 -4.071077 -2.492918   -6.113463    -6.7161
58
## 35    32.84228 -1.820107 -4.071077 -2.492918   -6.113463    -6.7161
58
## 36    36.78422 -1.820107 -4.071077 -2.492918   -6.113463    -6.7161
58
## 37    29.95520 -1.820107 -4.071077 -2.492918   -6.113463    -6.7161
58
## 38    37.76353 -1.820107 -4.071077 -2.492918   -6.113463    -6.7161
58
## 39    19.43967 -1.820107 -4.071077 -2.492918   -6.113463    -6.7161
58
## 40    21.28327 -1.820107 -4.071077 -2.492918   -6.113463    -6.7161
58
## 41    17.99877 -1.820107 -4.071077 -2.492918   -6.113463    -6.7161
58
## 42    32.99577 -1.820107 -4.071077 -2.492918   -6.113463    -6.7161
58
## 43    31.65969 -1.820107 -4.071077 -2.492918   -6.113463    -6.7161
58
## 44    22.79756 -1.820107 -4.071077 -2.492918   -6.113463    -6.7161
58
## 45    29.74230 -1.820107 -4.071077 -2.492918   -6.113463    -6.7161
58
```

```
##    txplacebo:appt_catmid txplacebo:appt_catpost
## 1               2.309687               0.5640104
## 2               2.309687               0.5640104
## 3               2.309687               0.5640104
## 4               2.309687               0.5640104
## 5               2.309687               0.5640104
## 6               2.309687               0.5640104
## 7               2.309687               0.5640104
## 8               2.309687               0.5640104
## 9               2.309687               0.5640104
## 10              2.309687               0.5640104
## 11              2.309687               0.5640104
## 12              2.309687               0.5640104
## 13              2.309687               0.5640104
## 14              2.309687               0.5640104
## 15              2.309687               0.5640104
## 16              2.309687               0.5640104
## 17              2.309687               0.5640104
## 18              2.309687               0.5640104
## 19              2.309687               0.5640104
## 20              2.309687               0.5640104
## 21              2.309687               0.5640104
## 22              2.309687               0.5640104
## 23              2.309687               0.5640104
## 24              2.309687               0.5640104
## 25              2.309687               0.5640104
## 26              2.309687               0.5640104
## 27              2.309687               0.5640104
## 28              2.309687               0.5640104
## 29              2.309687               0.5640104
## 30              2.309687               0.5640104
## 31              2.309687               0.5640104
## 32              2.309687               0.5640104
## 33              2.309687               0.5640104
## 34              2.309687               0.5640104
## 35              2.309687               0.5640104
## 36              2.309687               0.5640104
## 37              2.309687               0.5640104
## 38              2.309687               0.5640104
## 39              2.309687               0.5640104
## 40              2.309687               0.5640104
## 41              2.309687               0.5640104
## 42              2.309687               0.5640104
## 43              2.309687               0.5640104
## 44              2.309687               0.5640104
## 45              2.309687               0.5640104
```
